# Supplementary material for: Condensation of Ede1 promotes the initiation of endocytosis
Source: eLife. 2022 Apr 12;11:e72865. doi: 10.7554/eLife.72865 (PMC9064294; doi:10.7554/eLife.72865)
Supplement: Figure 3—source data 1. [file elife-72865-fig3-data1.zip › fig3_data1.html]

Ede1 levels in 3∆ and OE strains


Code 

- Download Rmd

# Ede1 levels in 3∆ and OE strains

#### Last compiled on March 25, 2022

# 

## Experiment

### Premise

The aim of the experiment is to visualize the changes in total and cytosolic concentrations of EGFP-tagged Ede1 in the three strains used throughout the paper: wild-type background, 3∆, and overexpression driven by the ADH1 promoter.

### Imaging

All strains within one dataset were imaged on the same day and in the same conditions on the Nikon Ti microscope equipped with a Yokogawa CSW1 spinning disk and a Photometrics Prime 95B sCMOS camera. The Cellular and Cytosolic acquisitions were taken on different days so they are not directly comparable; the only meaningful comparison is between different strains within one localization / dataset combination.

Two different series of exposures were taken: high exposure for cytosolic quantification (saturated condensates) and low exposure for total quantification (avoiding any saturation). The cytosolic quantification was done on cells co-expressing Abp1-mCherry, and the total on cells co-expressing Rvs167-mCherry (explanation below).

#### Strain list

| strain | nickname | mCherry |
| --- | --- | --- |
| MKY0381 | wt | Abp1 |
| MKY0583 | 3Δ | Abp1 |
| MKY3448 | oe | Abp1 |
| MKY4450 | wt | Rvs167 |
| MKY3487 | 3Δ | Rvs167 |
| MKY3488 | oe | Rvs167 |

#### Cytosolic intensity

Single planes were acquired at the equatorial plane using 488 nm laser at 100% power, with 500 ms exposure, and camera gain level 2.

The three strains used were the ones co-expressing Abp1-mCherry.

#### Total cellular intensity

Two-color stacks were acquired with 0.2 μm spacing and 5 μm range around the equatorial plane.

- Ede1-EGFP: 488 nm laser at 15% power, with 100 ms exposure, and camera gain level 2.
- Rvs167-mCherry: 561 nm laser at 100% power, with 500 ms exposure, and camera gain level 3.

Initially both the Cytosolic and Cellular datasets were taken during the same acquisition, using the Abp1-mCherry strains. However, Abp1 turned out suboptimal for masking entire cells (too much contrast between sites and cytosol), and I repeated the experiment with the Rvs167 strains. The diffuse signal of Rvs167-mCherry makes it a perfect protein to create cellular masks. The results were ultimately very similar on average, but I choose to show Ede1-GFP/Rvs167-mCherry because the quantification is more precise.

### Processing

All images were background-subtracted using the ImageJ rolling ball algorithm with a 50px radius.

#### Cytosolic intensity

5x5 px square regions away from the condensates and vacuoles were manually selected in ImageJ and mean pixel intensity was saved to file.

#### Total cellular intensity

Individual cells were cropped in ImageJ and the function `batch_mask` from my personal Python package `mkimage` was used to quantify intensity. Briefly, the RFP channel was median-filtered with a disk brush of 10 px radius, and thresholded using Li’s method followed by one round of morphological opening to create a mask. The mask was applied to GFP images and summary statistics of the masked regions (mean, median, sd) were saved.

#### Data clean-up

The mean pixel values of cytosolic regions and entire cells were loaded from text files using a separate R notebook. That notebook was used only to gather all observations in the tidy data structures accessible here, without any further processing.

## Data summary

The table below summarizes intensity measurements from each dataset, as well as normalized intensities. The normalization was performed for each dataset by dividing each observation by the mean wild-type intensity for that dataset.

| localization | strain | dataset | n | intensity\_mean | intensity\_sd | intensity\_median | intensity\_se | normalized\_mean | normalized\_sd | normalized\_median | normalized\_se |
| --- | --- | --- | --- | --- | --- | --- | --- | --- | --- | --- | --- |
| Cellular | wt | 1 | 120 | 4.464492 | 0.9466484 | 4.2845 | 0.0864168 | 1.0000000 | 0.2120395 | 0.9596837 | 0.0193565 |
| Cellular | wt | 2 | 120 | 4.341617 | 0.8611170 | 4.2470 | 0.0786089 | 1.0000000 | 0.1983402 | 0.9782070 | 0.0181059 |
| Cellular | wt | 3 | 120 | 4.428617 | 0.9096351 | 4.3150 | 0.0830379 | 1.0000000 | 0.2053994 | 0.9743449 | 0.0187503 |
| Cellular | 3Δ | 1 | 120 | 5.262183 | 1.2097965 | 5.2855 | 0.1104388 | 1.1786747 | 0.2709819 | 1.1838974 | 0.0247372 |
| Cellular | 3Δ | 2 | 120 | 5.507100 | 1.2369592 | 5.4540 | 0.1129184 | 1.2684446 | 0.2849075 | 1.2562141 | 0.0260084 |
| Cellular | 3Δ | 3 | 117 | 5.567872 | 1.2579994 | 5.4290 | 0.1163021 | 1.2572485 | 0.2840615 | 1.2258907 | 0.0262615 |
| Cellular | oe | 1 | 120 | 13.907208 | 5.2141612 | 13.4065 | 0.4759856 | 3.1150710 | 1.1679182 | 3.0029175 | 0.1066159 |
| Cellular | oe | 2 | 119 | 14.113403 | 5.8849060 | 13.5730 | 0.5394684 | 3.2507254 | 1.3554642 | 3.1262548 | 0.1242552 |
| Cellular | oe | 3 | 120 | 17.306900 | 6.4476950 | 17.1480 | 0.5885913 | 3.9079698 | 1.4559163 | 3.8720895 | 0.1329064 |
| Cytosolic | wt | 1 | 141 | 13.337979 | 3.3300969 | 12.9200 | 0.2804448 | 1.0000000 | 0.2496703 | 0.9686625 | 0.0210260 |
| Cytosolic | wt | 2 | 140 | 14.197786 | 3.1509078 | 13.6800 | 0.2663003 | 1.0000000 | 0.2219295 | 0.9635305 | 0.0187565 |
| Cytosolic | wt | 3 | 142 | 10.774648 | 2.1687790 | 10.7000 | 0.1819999 | 1.0000000 | 0.2012854 | 0.9930719 | 0.0168915 |
| Cytosolic | 3Δ | 1 | 136 | 14.372368 | 4.1426283 | 13.8600 | 0.3552274 | 1.0775521 | 0.3105889 | 1.0391380 | 0.0266328 |
| Cytosolic | 3Δ | 2 | 140 | 13.182186 | 3.9816074 | 12.9400 | 0.3365072 | 0.9284677 | 0.2804386 | 0.9114097 | 0.0237014 |
| Cytosolic | 3Δ | 3 | 140 | 10.770571 | 2.5775639 | 10.4600 | 0.2178439 | 0.9996217 | 0.2392249 | 0.9707974 | 0.0202182 |
| Cytosolic | oe | 1 | 135 | 13.519489 | 3.7559074 | 12.8400 | 0.3232570 | 1.0136085 | 0.2815949 | 0.9626646 | 0.0242358 |
| Cytosolic | oe | 2 | 140 | 14.344321 | 3.0766393 | 14.0000 | 0.2600235 | 1.0103210 | 0.2166985 | 0.9860693 | 0.0183144 |
| Cytosolic | oe | 3 | 140 | 11.012286 | 2.7679976 | 10.7000 | 0.2339385 | 1.0220553 | 0.2568991 | 0.9930719 | 0.0217119 |

### Aggregated normalized means

Quick summary of `normalized mean` from above expressed as average values of repeat-level means with 95% confidence intervals:

| localization | strain | mean | lower | upper |
| --- | --- | --- | --- | --- |
| Cellular | wt | 1.00 | 1.00 | 1.00 |
| Cellular | 3Δ | 1.23 | 1.11 | 1.36 |
| Cellular | oe | 3.42 | 2.37 | 4.48 |
| Cytosolic | wt | 1.00 | 1.00 | 1.00 |
| Cytosolic | 3Δ | 1.00 | 0.82 | 1.19 |
| Cytosolic | oe | 1.02 | 1.00 | 1.03 |

This hints that the true mean of cytosolic fluorescence is unchanged in different strains, and that 3Δ and overexpression strains feature some (sgnificant?) levels of overexpression. More accurate estimates and p-values can be found in the ‘Modeling’ tab.

## Plots

I have chosen to show this data using the SuperPlot style. Each point shows mean intensity of a small region in the cytoplasm of an individual cell, or one entire cell (separated into facets).

Big colour points show mean measurements from three independent repeats.

Range is mean +/- SD, calculated based on the three independent repeats.

### Without normalization

These plots show raw intensity measures. The dataset effect is not so bad in the Cellular data, which were imaged 3 days in a row. It is quite pronounced in the Cytosolic data, where the facility realigned the laser fiber in the microscope between datasets 2 and 3, affecting the absolute values.

Also, if it seems weird that the cytosolic values are higher than the cellular ones, that is not accidental. The exposure of the cellular data had to be kept low in order to avoid saturating the condensates. In the cytosolic data, the exposures were high in order to maximize the cytosolic signal, allowing for the condensates to saturate.

### Normalized

All observations have been scaled to the mean value of the wild-type strain of each dataset. This looks good, but has the unfortunate side-effect of zeroing the variance of the dataset means in the wild-type strain.

This breaks all the assumptions of ANOVA if we wanted to run it on repeat-level means. It will be better to show the raw data, and make a linear model which accounts for batch effects to get estimated means, effect sizes and p-values.

## Modeling

We want to find out how the mean intensity values of cytoplasm or entire cells depend on the strain used (null hypothesis is that they do not).

In other experiments I followed the recommendations of several papers and performed hypothesis tests on repeat-level means.

This approach takes between-experiment variability into account, and avoids p-value inflation, where tiny differences might end up ‘highly significant’ due to testing a large number of observations which are not necessarily independent.

But it’s also *very* conservative because using a small N of repeats means losing power when compared to the hundreds of cell-level observations.

### Linear mixed model

Because we have a complete block design (all strains are represented in all batches), we can include the dataset as a fixed or random effect. Strain is obviously the fixed effect we want to analyze. Because we do not care about estimating the effect of any particular batch, a mixed model with the dataset as a random effect will be appropriate.

#### Cellular

Fitting a linear mixed model using the `lmerTest` package (`lmer` comes from `lme4`, `lmerTest` just ads an interface for comparing models):

```
## Linear mixed model fit by REML. t-tests use Satterthwaite's method [
## lmerModLmerTest]
## Formula: intensity ~ strain + (1 | dataset)
##    Data: cell_data
## 
## REML criterion at convergence: 5798.7
## 
## Scaled residuals: 
##     Min      1Q  Median      3Q     Max 
## -3.2952 -0.3397 -0.0084  0.3135  7.2404 
## 
## Random effects:
##  Groups   Name        Variance Std.Dev.
##  dataset  (Intercept)  0.4261  0.6528  
##  Residual             12.7450  3.5700  
## Number of obs: 1076, groups:  dataset, 3
## 
## Fixed effects:
##              Estimate Std. Error        df t value Pr(>|t|)    
## (Intercept)    4.4116     0.4212    2.6582  10.473 0.003101 ** 
## strain3Δ       1.0392     0.2667 1071.0032   3.897 0.000103 ***
## strainoe      10.6995     0.2663 1070.9998  40.182  < 2e-16 ***
## ---
## Signif. codes:  0 '***' 0.001 '**' 0.01 '*' 0.05 '.' 0.1 ' ' 1
## 
## Correlation of Fixed Effects:
##          (Intr) strn3Δ
## strain3Δ -0.315       
## strainoe -0.316  0.499
```

We have significant effect of `strain` on intensity according to an F-test:

```
## Type III Analysis of Variance Table with Satterthwaite's method
##        Sum Sq Mean Sq NumDF DenDF F value    Pr(>F)    
## strain  24995   12497     2  1071  980.57 < 2.2e-16 ***
## ---
## Signif. codes:  0 '***' 0.001 '**' 0.01 '*' 0.05 '.' 0.1 ' ' 1
```

`dataset` does not seem to explain much variance, but it definitely *could* due to the nature of measuring absolute fluorescence values, as we see with the Cytosolic data. It makes sense to keep it in the model.

In the plots we saw that maybe the data is not really normal, as is typical for fluorescence values. Model residuals are also not normal:

This is a *big* departure from normality, with a strong skew, although it looks better on the histogram More importantly, the variance in the data was also not homogeneous (bigger spread in OE intensities). We can see this reflected in the residuals:

This is really not good, but then again the conclusions of the experiment are obvious and do not hang on the quality of the modeling exercise. Probably log-transform or a GLM could help; lets stick to transforming the data.

We specify the LMM with `log(intensity) ~ strain + (1|dataset)`:

```
## Linear mixed model fit by REML. t-tests use Satterthwaite's method [
## lmerModLmerTest]
## Formula: log(intensity) ~ strain + (1 | dataset)
##    Data: cell_data
## 
## REML criterion at convergence: 479
## 
## Scaled residuals: 
##     Min      1Q  Median      3Q     Max 
## -4.2742 -0.6000  0.0491  0.6506  3.6589 
## 
## Random effects:
##  Groups   Name        Variance Std.Dev.
##  dataset  (Intercept) 0.002219 0.04711 
##  Residual             0.089617 0.29936 
## Number of obs: 1076, groups:  dataset, 3
## 
## Fixed effects:
##              Estimate Std. Error        df t value Pr(>|t|)    
## (Intercept) 1.464e+00  3.144e-02 2.885e+00  46.546 3.07e-05 ***
## strain3Δ    2.056e-01  2.236e-02 1.071e+03   9.195  < 2e-16 ***
## strainoe    1.168e+00  2.233e-02 1.071e+03  52.310  < 2e-16 ***
## ---
## Signif. codes:  0 '***' 0.001 '**' 0.01 '*' 0.05 '.' 0.1 ' ' 1
## 
## Correlation of Fixed Effects:
##          (Intr) strn3Δ
## strain3Δ -0.354       
## strainoe -0.355  0.499
```

We again have strong effect of `strain` and a random effect with very little explanatory power for `dataset`. Let’s look at Q-Q plot and histogram of model residuals:

The residual distribution is still fat-tailed and skews slightly left now, but the transformation helped. More importantly, the differences in spread also decreased, although they are still there:

We could try a gamma GLM, but again, the absolute quality of the model is not critical to the conclusions here so let’s not over complicate.

I say we stop here and evaluate the group contrasts. The `emmeans` package has a wonderful interface for pairwise contrasts, *and* it computes group means with confidence intervals.

The means and CIs are computed in log-space and back-transformed, giving the absolute fluorescence values:

| strain | response | std.error | df | conf.low | conf.high |
| --- | --- | --- | --- | --- | --- |
| wt | 4.321147 | 0.1358662 | 2.885930 | 3.900804 | 4.786785 |
| 3Δ | 5.307548 | 0.1670583 | 2.898204 | 4.791964 | 5.878605 |
| oe | 13.895157 | 0.4370472 | 2.889984 | 12.544115 | 15.391711 |

We can also compute differences between groups along with their CIs and p-values for pairwise comparisons. Because the differences are computed in log-space, we get ratios after back-transforming. This is actually very useful because the ratio to wild-type is what we wanted to know in the first place.

| contrast | ratio | SE | df | lower.CL | upper.CL | null | t.ratio | p.value |
| --- | --- | --- | --- | --- | --- | --- | --- | --- |
| 3Δ / wt | 1.228273 | 0.0274644 | 1071.005 | 1.165477 | 1.294452 | 1 | 9.195339 | 0 |
| oe / wt | 3.215618 | 0.0718002 | 1071.001 | 3.051444 | 3.388625 | 1 | 52.310471 | 0 |
| oe / 3Δ | 2.617999 | 0.0585798 | 1071.007 | 2.484062 | 2.759158 | 1 | 43.011254 | 0 |

Finally, the `emmeans` interface allows us to plot the modeled means along with confidence intervals (bars) and confidence intervals for the group differences (arrows).

We can conclude that all groups are significantly different, with extremely low p-values (not that it matters). 3∆ background increases Ede1 levels by a modest ~20%, and ADH1-driven overexpression by ~200%.

#### Cytosolic

After looking at the plots, we go into this exercise already suspecting that `strain` has no effect. But let’s build a model as before:

```
## Linear mixed model fit by REML. t-tests use Satterthwaite's method [
## lmerModLmerTest]
## Formula: intensity ~ strain + (1 | dataset)
##    Data: cyto_data
## 
## REML criterion at convergence: 6555.7
## 
## Scaled residuals: 
##     Min      1Q  Median      3Q     Max 
## -2.4170 -0.6684 -0.1093  0.5928  3.5146 
## 
## Random effects:
##  Groups   Name        Variance Std.Dev.
##  dataset  (Intercept)  2.925   1.710   
##  Residual             10.810   3.288   
## Number of obs: 1254, groups:  dataset, 3
## 
## Fixed effects:
##               Estimate Std. Error         df t value Pr(>|t|)   
## (Intercept)  1.277e+01  1.000e+00  2.070e+00  12.766  0.00533 **
## strain3Δ    -8.837e-04  2.270e-01  1.249e+03  -0.004  0.99689   
## strainoe     1.936e-01  2.272e-01  1.249e+03   0.852  0.39434   
## ---
## Signif. codes:  0 '***' 0.001 '**' 0.01 '*' 0.05 '.' 0.1 ' ' 1
## 
## Correlation of Fixed Effects:
##          (Intr) strn3Δ
## strain3Δ -0.113       
## strainoe -0.112  0.496
```

After modeling the fixed effect of `strain`, dataset explains ~25% of the variance. `strain` itself is not a significant predictor:

```
## Type III Analysis of Variance Table with Satterthwaite's method
##        Sum Sq Mean Sq NumDF DenDF F value Pr(>F)
## strain  10.45  5.2249     2  1249  0.4833 0.6168
```

We really could stop here, since `strain` does not explain anything, and that’s as clear from the model as from the plots. But let’s check model residuals:

This is not so bad actually but we have some fat tails, and a small difference in spread for larger values. Log-transform helped before so let’s see what it does here:

```
## Linear mixed model fit by REML. t-tests use Satterthwaite's method [
## lmerModLmerTest]
## Formula: log(intensity) ~ strain + (1 | dataset)
##    Data: cyto_data
## 
## REML criterion at convergence: 146.3
## 
## Scaled residuals: 
##     Min      1Q  Median      3Q     Max 
## -4.0764 -0.6464  0.0103  0.6726  2.9964 
## 
## Random effects:
##  Groups   Name        Variance Std.Dev.
##  dataset  (Intercept) 0.01834  0.1354  
##  Residual             0.06437  0.2537  
## Number of obs: 1254, groups:  dataset, 3
## 
## Fixed effects:
##               Estimate Std. Error         df t value Pr(>|t|)    
## (Intercept)  2.515e+00  7.916e-02  2.066e+00  31.770 0.000822 ***
## strain3Δ    -1.259e-02  1.752e-02  1.249e+03  -0.719 0.472512    
## strainoe     9.595e-03  1.753e-02  1.249e+03   0.547 0.584248    
## ---
## Signif. codes:  0 '***' 0.001 '**' 0.01 '*' 0.05 '.' 0.1 ' ' 1
## 
## Correlation of Fixed Effects:
##          (Intr) strn3Δ
## strain3Δ -0.110       
## strainoe -0.110  0.496
```

This is better, error-wise:

But `strain` is still not significant:

```
## Type III Analysis of Variance Table with Satterthwaite's method
##         Sum Sq  Mean Sq NumDF DenDF F value Pr(>F)
## strain 0.10288 0.051442     2  1249  0.7991 0.4499
```

And if we use `emmeans` to get contrasts, we find no significant differences between groups:

| contrast | ratio | SE | df | lower.CL | upper.CL | null | t.ratio | p.value |
| --- | --- | --- | --- | --- | --- | --- | --- | --- |
| 3Δ / wt | 0.9874892 | 0.0173002 | 1249.001 | 0.9477174 | 1.028930 | 1 | -0.7186153 | 0.7525132 |
| oe / wt | 1.0096410 | 0.0176992 | 1249.001 | 0.9689525 | 1.052038 | 1 | 0.5473328 | 0.8478621 |
| oe / 3Δ | 1.0224324 | 0.0179974 | 1249.000 | 0.9810616 | 1.065548 | 1 | 1.2602988 | 0.4180250 |

Since fluorescence intensity values are not meaningful, I am skipping the estimated group means here.

## Conclusions

1. We cannot detect any differences in cytosolic Ede1 intensities across different strains.
2. We see significant differences in mean total cellular levels of Ede1, namely:

   - ~120% of wild-type Ede1 levels in 3∆ strains, and
   - ~320% of wild-type Ede1 levels in ADH1-driven overexpression.

A table summarising all group contrasts:

| contrast | ratio | lower.CL | upper.CL | localization |
| --- | --- | --- | --- | --- |
| 3Δ / wt | 0.99 | 0.95 | 1.03 | Cytosolic |
| oe / wt | 1.01 | 0.97 | 1.05 | Cytosolic |
| oe / 3Δ | 1.02 | 0.98 | 1.07 | Cytosolic |
| 3Δ / wt | 1.23 | 1.17 | 1.29 | Cellular |
| oe / wt | 3.22 | 3.05 | 3.39 | Cellular |
| oe / 3Δ | 2.62 | 2.48 | 2.76 | Cellular |

## Source data

### .csv

Download intensities.csv

### .RData

Download data\_clean.RData

## Session info

R session used to generate this document.

```
## R version 4.1.0 (2021-05-18)
## Platform: x86_64-apple-darwin17.0 (64-bit)
## Running under: macOS Big Sur 10.16
## 
## Matrix products: default
## BLAS:   /Library/Frameworks/R.framework/Versions/4.1/Resources/lib/libRblas.dylib
## LAPACK: /Library/Frameworks/R.framework/Versions/4.1/Resources/lib/libRlapack.dylib
## 
## locale:
## [1] en_US.UTF-8/en_US.UTF-8/en_US.UTF-8/C/en_US.UTF-8/en_US.UTF-8
## 
## attached base packages:
## [1] stats     graphics  grDevices utils     datasets  methods   base     
## 
## other attached packages:
##  [1] emmeans_1.7.2    lmerTest_3.1-3   lme4_1.1-27.1    Matrix_1.3-3    
##  [5] ggsignif_0.6.2   ggbeeswarm_0.6.0 broom_0.7.9      knitr_1.36      
##  [9] forcats_0.5.1    stringr_1.4.0    dplyr_1.0.7      purrr_0.3.4     
## [13] readr_2.0.1      tidyr_1.1.3      tibble_3.1.3     ggplot2_3.3.5   
## [17] tidyverse_1.3.1 
## 
## loaded via a namespace (and not attached):
##  [1] TH.data_1.1-0       minqa_1.2.4         colorspace_2.0-2   
##  [4] ellipsis_0.3.2      estimability_1.3    htmlTable_2.2.1    
##  [7] base64enc_0.1-3     fs_1.5.0            rstudioapi_0.13    
## [10] farver_2.1.0        fansi_0.5.0         mvtnorm_1.1-3      
## [13] lubridate_1.7.10    xml2_1.3.2          codetools_0.2-18   
## [16] splines_4.1.0       Formula_1.2-4       jsonlite_1.7.2     
## [19] nloptr_1.2.2.2      pbkrtest_0.5.1      cluster_2.1.2      
## [22] dbplyr_2.1.1        png_0.1-7           compiler_4.1.0     
## [25] httr_1.4.2          backports_1.2.1     assertthat_0.2.1   
## [28] cli_3.1.0           htmltools_0.5.1.1   tools_4.1.0        
## [31] gtable_0.3.0        glue_1.4.2          Rcpp_1.0.7         
## [34] cellranger_1.1.0    jquerylib_0.1.4     vctrs_0.3.8        
## [37] nlme_3.1-152        xfun_0.25           rvest_1.0.1        
## [40] mime_0.11           lifecycle_1.0.0     MASS_7.3-54        
## [43] zoo_1.8-9           scales_1.1.1        hms_1.1.0          
## [46] parallel_4.1.0      sandwich_3.0-1      RColorBrewer_1.1-2 
## [49] yaml_2.2.1          gridExtra_2.3       rpart_4.1-15       
## [52] latticeExtra_0.6-29 stringi_1.7.3       highr_0.9          
## [55] checkmate_2.0.0     boot_1.3-28         rlang_0.4.11       
## [58] pkgconfig_2.0.3     evaluate_0.14       lattice_0.20-44    
## [61] htmlwidgets_1.5.3   labeling_0.4.2      tidyselect_1.1.1   
## [64] magrittr_2.0.1      R6_2.5.1            generics_0.1.0     
## [67] Hmisc_4.5-0         multcomp_1.4-18     DBI_1.1.1          
## [70] pillar_1.6.2        haven_2.4.3         foreign_0.8-81     
## [73] withr_2.4.2         survival_3.2-11     nnet_7.3-16        
## [76] modelr_0.1.8        crayon_1.4.1        utf8_1.2.2         
## [79] tzdb_0.1.2          rmarkdown_2.11      jpeg_0.1-9         
## [82] grid_4.1.0          readxl_1.3.1        data.table_1.14.0  
## [85] reprex_2.0.1        digest_0.6.27       xtable_1.8-4       
## [88] numDeriv_2016.8-1.1 munsell_0.5.0       beeswarm_0.4.0     
## [91] vipor_0.4.5
```

LS0tCnRpdGxlOiAiRWRlMSBsZXZlbHMgaW4gM+KIhiBhbmQgT0Ugc3RyYWlucyIKZGF0ZTogIkxhc3QgY29tcGlsZWQgb24gYHIgZm9ybWF0KFN5cy50aW1lKCksICclQiAlZCwgJVknKWAiCm91dHB1dDoKICBodG1sX2RvY3VtZW50OgogICAgY29kZV9kb3dubG9hZDogVFJVRQotLS0KCmBgYHtyIHNldHVwLCBpbmNsdWRlPUZBTFNFfQprbml0cjo6b3B0c19jaHVuayRzZXQoZWNobyA9IEZBTFNFLCBtZXNzYWdlPUZBTFNFLCB3YXJuaW5nPUZBTFNFLAogICAgICAgICAgICAgICAgICAgICAgZHBpID0gOTYsIGZpZy53aWR0aCA9NiwgZmlnLmhlaWdodCA9IDQpCmBgYAoKYGBge3IgbGlic30KbGlicmFyeSh0aWR5dmVyc2UpCmxpYnJhcnkoa25pdHIpCmxpYnJhcnkoYnJvb20pCgojIGdncGxvdDIgZXh0ZW5zaW9ucwpsaWJyYXJ5KGdnYmVlc3dhcm0pCmxpYnJhcnkoZ2dzaWduaWYpCgojIGZvciB0aGUgbGluZWFyIG1peGVkIG1vZGVscwpsaWJyYXJ5KGxtZXJUZXN0KQpsaWJyYXJ5KGVtbWVhbnMpCmBgYAoKYGBge3IgbG9hZH0KIyBMb2FkIGRhdGEgZ2VuZXJhdGVkIGJ5IGNsZWFudXAgbm90ZWJvb2sKcm0obGlzdCA9IGxzKCkpCmxvYWQoJ2RhdGEvZGF0YV9jbGVhbi5SRGF0YScpCmBgYAoKYGBge3IgdGhlbWV9CiMgQ3VzdG9tIGdncGxvdDIgdGhlbWUKIyAtLS0tLS0tLS0tLS0tLS0tLS0tLQoKIyBtaW5pbWFsIHRoZW1lIHdpdGggYm9yZGVyCiMgYmFzZWQgb24gdGhlbWVfbGluZWRyYXcgd2l0aG91dCB0aGUgZ3JpZCBsaW5lcwojIGFsc28gdHJ5aW5nIHRvIHJlbW92ZSBhbGwgYmFja2dyb3VuZHMgYW5kIG1hcmdpbnMKIyB0aGUgYWltIGlzIHRvIG1ha2UgaXQgYXMgZWFzeSBhcyBwb3NzaWJsZSB0byBlZGl0IGluIGlsbHVzdHJhdG9yCgp0aGVtZV9jbGVhbiA8LSBmdW5jdGlvbihiYXNlX3NpemUgPSAxMSwgYmFzZV9mYW1pbHkgPSAiIiwKICAgICAgICAgICAgICAgICAgICAgICAgYmFzZV9saW5lX3NpemUgPSBiYXNlX3NpemUgLyAyMiwKICAgICAgICAgICAgICAgICAgICAgICAgYmFzZV9yZWN0X3NpemUgPSBiYXNlX3NpemUgLyAyMikgewogIHRoZW1lX2xpbmVkcmF3KAogICAgYmFzZV9zaXplID0gYmFzZV9zaXplLAogICAgYmFzZV9mYW1pbHkgPSBiYXNlX2ZhbWlseSwKICAgIGJhc2VfbGluZV9zaXplID0gYmFzZV9saW5lX3NpemUsCiAgICBiYXNlX3JlY3Rfc2l6ZSA9IGJhc2VfcmVjdF9zaXplCiAgKSAlK3JlcGxhY2UlCiAgICB0aGVtZSgKICAgICAgIyBubyBncmlkIGFuZCBubyBiYWNrZ3JvdW5kcyBpZiBJIGNhbiBoZWxwIGl0CiAgICAgIGxlZ2VuZC5iYWNrZ3JvdW5kID0gIGVsZW1lbnRfYmxhbmsoKSwKICAgICAgcGFuZWwuYmFja2dyb3VuZCA9IGVsZW1lbnRfYmxhbmsoKSwKICAgICAgcGFuZWwuZ3JpZCA9IGVsZW1lbnRfYmxhbmsoKSwKICAgICAgcGxvdC5iYWNrZ3JvdW5kID0gZWxlbWVudF9ibGFuaygpLAogICAgICBwbG90Lm1hcmdpbiA9IG1hcmdpbigwLCAwLCAwLCAwKSwKICAgICAgY29tcGxldGUgPSBUUlVFCiAgICApCn0KCiMgU2V0IGRlZmF1bHQgdGhlbWUKIyAtLS0tLS0tLS0tLS0tLS0tLQp0aGVtZV9zZXQodGhlbWVfY2xlYW4oYmFzZV9zaXplID0gMTQsIGJhc2VfZmFtaWx5ID0gIk15cmlhZCBQcm8iKSkKCiMgQ3JlYXRlIGEgZ2dzYXZlIHdyYXBwZXIKIyAtLS0tLS0tLS0tLS0tLS0tLS0tLS0tLQoKIyBUaGlzIHdheSB3ZSBjYW4gc2V0IGEgZGVmYXVsdCBzaXplIGFuZCBkZXZpY2UgZm9yIGFsbCBwbG90cwpteV9nZ3NhdmUgPC0gZnVuY3Rpb24oZmlsZW5hbWUsIHBsb3QgPSBsYXN0X3Bsb3QoKSwKICAgICAgICAgICAgICAgICAgICAgIGRldmljZSA9IGNhaXJvX3BkZiwgdW5pdHMgPSAibW0iLAogICAgICAgICAgICAgICAgICAgICAgd2lkdGggPSAxMDAsIGhlaWdodCA9IDgwLCAuLi4pewogIGdnc2F2ZShmaWxlbmFtZSA9IGZpbGVuYW1lLCBwbG90ID0gcGxvdCwKICAgICAgICAgZGV2aWNlID0gZGV2aWNlLCB1bml0cyA9IHVuaXRzLAogICAgICAgICBoZWlnaHQgPSBoZWlnaHQsIHdpZHRoID0gd2lkdGgsICAuLi4pCiAgfQpgYGAKCiMgey50YWJzZXQgLnRhYnNldC1waWxsc30KCiMjIEV4cGVyaW1lbnQKCiMjIyBQcmVtaXNlCgpUaGUgYWltIG9mIHRoZSBleHBlcmltZW50IGlzIHRvIHZpc3VhbGl6ZSB0aGUgY2hhbmdlcwppbiB0b3RhbCBhbmQgY3l0b3NvbGljIGNvbmNlbnRyYXRpb25zIG9mIEVHRlAtdGFnZ2VkIEVkZTEgCmluIHRoZSB0aHJlZSBzdHJhaW5zIHVzZWQgdGhyb3VnaG91dCB0aGUgcGFwZXI6IAogIHdpbGQtdHlwZSBiYWNrZ3JvdW5kLCAKICAz4oiGLAogIGFuZCBvdmVyZXhwcmVzc2lvbiBkcml2ZW4gYnkgdGhlIEFESDEgcHJvbW90ZXIuCgojIyMgSW1hZ2luZwoKQWxsIHN0cmFpbnMgd2l0aGluIG9uZSBkYXRhc2V0IAp3ZXJlIGltYWdlZCBvbiB0aGUgc2FtZSBkYXkgYW5kIGluIHRoZSBzYW1lIGNvbmRpdGlvbnMgCm9uIHRoZSBOaWtvbiBUaSBtaWNyb3Njb3BlCmVxdWlwcGVkIHdpdGggYSBZb2tvZ2F3YSBDU1cxIHNwaW5uaW5nIGRpc2sKYW5kIGEgUGhvdG9tZXRyaWNzIFByaW1lIDk1QiBzQ01PUyBjYW1lcmEuIApUaGUgQ2VsbHVsYXIgYW5kIEN5dG9zb2xpYyBhY3F1aXNpdGlvbnMgCndlcmUgdGFrZW4gb24gZGlmZmVyZW50IGRheXMKc28gdGhleSBhcmUgbm90IGRpcmVjdGx5IGNvbXBhcmFibGU7IAp0aGUgb25seSBtZWFuaW5nZnVsIGNvbXBhcmlzb24gCmlzIGJldHdlZW4gZGlmZmVyZW50IHN0cmFpbnMgd2l0aGluIG9uZSBsb2NhbGl6YXRpb24gLyBkYXRhc2V0IGNvbWJpbmF0aW9uLgoKVHdvIGRpZmZlcmVudCBzZXJpZXMgb2YgZXhwb3N1cmVzIHdlcmUgdGFrZW46IApoaWdoIGV4cG9zdXJlIGZvciBjeXRvc29saWMgcXVhbnRpZmljYXRpb24gKHNhdHVyYXRlZCBjb25kZW5zYXRlcykKYW5kIGxvdyBleHBvc3VyZSBmb3IgdG90YWwgcXVhbnRpZmljYXRpb24gKGF2b2lkaW5nIGFueSBzYXR1cmF0aW9uKS4KVGhlIGN5dG9zb2xpYyBxdWFudGlmaWNhdGlvbiB3YXMgZG9uZSBvbiBjZWxscyBjby1leHByZXNzaW5nIEFicDEtbUNoZXJyeSwKYW5kIHRoZSB0b3RhbCBvbiBjZWxscyBjby1leHByZXNzaW5nIFJ2czE2Ny1tQ2hlcnJ5IChleHBsYW5hdGlvbiBiZWxvdykuCgojIyMjIFN0cmFpbiBsaXN0CgpgYGB7ciBzdHJhaW5zfQprYWJsZShzdHJhaW5zKQpgYGAKCiMjIyMgQ3l0b3NvbGljIGludGVuc2l0eQoKU2luZ2xlIHBsYW5lcyB3ZXJlIGFjcXVpcmVkIGF0IHRoZSBlcXVhdG9yaWFsIHBsYW5lIHVzaW5nCiAgNDg4IG5tIGxhc2VyIGF0IDEwMCUgcG93ZXIsIAogIHdpdGggNTAwIG1zIGV4cG9zdXJlLAogIGFuZCBjYW1lcmEgZ2FpbiBsZXZlbCAyLgoKVGhlIHRocmVlIHN0cmFpbnMgdXNlZCB3ZXJlIHRoZSBvbmVzIGNvLWV4cHJlc3NpbmcgQWJwMS1tQ2hlcnJ5LgoKIyMjIyBUb3RhbCBjZWxsdWxhciBpbnRlbnNpdHkKClR3by1jb2xvciBzdGFja3Mgd2VyZSBhY3F1aXJlZCB3aXRoIAogIDAuMiDOvG0gc3BhY2luZwogIGFuZCA1IM68bSByYW5nZQogIGFyb3VuZCB0aGUgZXF1YXRvcmlhbCBwbGFuZS4gCgoqIEVkZTEtRUdGUDogNDg4IG5tIGxhc2VyIGF0IAogIDE1JSBwb3dlciwgCiAgd2l0aCAxMDAgbXMgZXhwb3N1cmUsCiAgYW5kIGNhbWVyYSBnYWluIGxldmVsIDIuCiogUnZzMTY3LW1DaGVycnk6IDU2MSBubSBsYXNlciBhdCAKICAxMDAlIHBvd2VyLCB3aXRoIAogIDUwMCBtcyBleHBvc3VyZSwKICBhbmQgY2FtZXJhIGdhaW4gbGV2ZWwgMy4KCkluaXRpYWxseSBib3RoIHRoZSBDeXRvc29saWMgYW5kIENlbGx1bGFyIGRhdGFzZXRzCndlcmUgdGFrZW4gZHVyaW5nIHRoZSBzYW1lIGFjcXVpc2l0aW9uLCAKdXNpbmcgdGhlIEFicDEtbUNoZXJyeSBzdHJhaW5zLgpIb3dldmVyLCBBYnAxIHR1cm5lZCBvdXQgc3Vib3B0aW1hbCBmb3IgbWFza2luZyBlbnRpcmUgY2VsbHMgCih0b28gbXVjaCBjb250cmFzdCBiZXR3ZWVuIHNpdGVzIGFuZCBjeXRvc29sKSwKYW5kIEkgcmVwZWF0ZWQgdGhlIGV4cGVyaW1lbnQgd2l0aCB0aGUgUnZzMTY3IHN0cmFpbnMuIApUaGUgZGlmZnVzZSBzaWduYWwgb2YgUnZzMTY3LW1DaGVycnkgCm1ha2VzIGl0IGEgcGVyZmVjdCBwcm90ZWluIHRvIGNyZWF0ZSBjZWxsdWxhciBtYXNrcy4gClRoZSByZXN1bHRzIHdlcmUgdWx0aW1hdGVseSB2ZXJ5IHNpbWlsYXIgb24gYXZlcmFnZSwgCmJ1dCBJIGNob29zZSB0byBzaG93IEVkZTEtR0ZQL1J2czE2Ny1tQ2hlcnJ5IApiZWNhdXNlIHRoZSBxdWFudGlmaWNhdGlvbiBpcyBtb3JlIHByZWNpc2UuCgojIyMgUHJvY2Vzc2luZwoKQWxsIGltYWdlcyB3ZXJlIGJhY2tncm91bmQtc3VidHJhY3RlZCB1c2luZyB0aGUgSW1hZ2VKIHJvbGxpbmcgYmFsbCBhbGdvcml0aG0gCndpdGggYSA1MHB4IHJhZGl1cy4KCiMjIyMgQ3l0b3NvbGljIGludGVuc2l0eQogCjV4NSBweCBzcXVhcmUgcmVnaW9ucyBhd2F5IGZyb20gdGhlIGNvbmRlbnNhdGVzIGFuZCB2YWN1b2xlcwp3ZXJlIG1hbnVhbGx5IHNlbGVjdGVkIGluIEltYWdlSiBhbmQgbWVhbiBwaXhlbCBpbnRlbnNpdHkgd2FzIHNhdmVkIHRvIGZpbGUuCgojIyMjIFRvdGFsIGNlbGx1bGFyIGludGVuc2l0eQoKSW5kaXZpZHVhbCBjZWxscyB3ZXJlIGNyb3BwZWQgaW4gSW1hZ2VKCmFuZCB0aGUgZnVuY3Rpb24gYGJhdGNoX21hc2tgIGZyb20gbXkgcGVyc29uYWwgUHl0aG9uIHBhY2thZ2UgYG1raW1hZ2VgCndhcyB1c2VkIHRvIHF1YW50aWZ5IGludGVuc2l0eS4gCkJyaWVmbHksIHRoZSBSRlAgY2hhbm5lbCB3YXMgCiAgbWVkaWFuLWZpbHRlcmVkIHdpdGggYSBkaXNrIGJydXNoIG9mIDEwIHB4IHJhZGl1cywgCiAgYW5kIHRocmVzaG9sZGVkIHVzaW5nIExpJ3MgbWV0aG9kIAogIGZvbGxvd2VkIGJ5IG9uZSByb3VuZCBvZiBtb3JwaG9sb2dpY2FsIG9wZW5pbmcKdG8gY3JlYXRlIGEgbWFzay4KVGhlIG1hc2sgd2FzIGFwcGxpZWQgdG8gR0ZQIGltYWdlcyBhbmQgc3VtbWFyeSBzdGF0aXN0aWNzIApvZiB0aGUgbWFza2VkIHJlZ2lvbnMgKG1lYW4sIG1lZGlhbiwgc2QpIHdlcmUgc2F2ZWQuCgojIyMjIERhdGEgY2xlYW4tdXAKClRoZSBtZWFuIHBpeGVsIHZhbHVlcyBvZiBjeXRvc29saWMgcmVnaW9ucyBhbmQgZW50aXJlIGNlbGxzIAp3ZXJlIGxvYWRlZCBmcm9tIHRleHQgZmlsZXMgdXNpbmcgYSBzZXBhcmF0ZSBSIG5vdGVib29rLiAKVGhhdCBub3RlYm9vayB3YXMgdXNlZCBvbmx5IHRvIGdhdGhlciBhbGwgb2JzZXJ2YXRpb25zIAppbiB0aGUgdGlkeSBkYXRhIHN0cnVjdHVyZXMgYWNjZXNzaWJsZSBoZXJlLCB3aXRob3V0IGFueSBmdXJ0aGVyIHByb2Nlc3NpbmcuCgojIyBEYXRhIHN1bW1hcnkKCmBgYHtyIHNjYWxlfQojIGdlbmVyYXRlIG5vcm1hbGl6ZWQgaW50ZW5zaXRpZXMgKHdpdGhpbiBlYWNoIGRhdGFzZXQpCgojIHB1bGwgb3V0IHNjYWxpbmcgZmFjdG9yCnd0X21lYW5zIDwtIGludGVuc2l0aWVzICU+JQogIGZpbHRlcihzdHJhaW4gPT0gJ3d0JykgJT4lCiAgZ3JvdXBfYnkoZGF0YXNldCwgbG9jYWxpemF0aW9uKSAlPiUKICBzdW1tYXJpc2Uod3RfbWVhbiA9IG1lYW4oaW50ZW5zaXR5KSwgLmdyb3VwcyA9ICdkcm9wJykKCiMgam9pbiB3aXRoIHRoZSBkYXRhZnJhbWUsIGdlbmVyYXRlIG5ldyB2YXJpYWJsZQppbnRlbnNpdGllcyA8LSBsZWZ0X2pvaW4oaW50ZW5zaXRpZXMsIHd0X21lYW5zLAogICAgICAgICAgICAgICAgICAgICAgICAgYnkgPSBjKCdkYXRhc2V0JywgJ2xvY2FsaXphdGlvbicpKSAlPiUKICBtdXRhdGUobm9ybWFsaXplZCA9IGludGVuc2l0eSAvIHd0X21lYW4pCmBgYAoKVGhlIHRhYmxlIGJlbG93IHN1bW1hcml6ZXMgaW50ZW5zaXR5IG1lYXN1cmVtZW50cyBmcm9tIGVhY2ggZGF0YXNldCwKYXMgd2VsbCBhcyBub3JtYWxpemVkIGludGVuc2l0aWVzLiBUaGUgbm9ybWFsaXphdGlvbiB3YXMgcGVyZm9ybWVkIGZvciBlYWNoCmRhdGFzZXQgYnkgZGl2aWRpbmcgZWFjaCBvYnNlcnZhdGlvbiBieSB0aGUgbWVhbiB3aWxkLXR5cGUgaW50ZW5zaXR5CmZvciB0aGF0IGRhdGFzZXQuCgpgYGB7ciBzdW1tYXJpemVfbm9ybWFsfQojIGdlbmVyYXRlIHN1bW1hcnkgc3RhdGlzdGljcwppbnRlbnNpdHlfc3RhdHMgPC0gaW50ZW5zaXRpZXMgJT4lCiAgZ3JvdXBfYnkobG9jYWxpemF0aW9uLCBzdHJhaW4sIGRhdGFzZXQpICU+JQogIHN1bW1hcmlzZShuID0gbigpLAogICAgICAgICAgICBhY3Jvc3MoYyhpbnRlbnNpdHksIG5vcm1hbGl6ZWQpLAogICAgICAgICAgICAgICAgICAgbGlzdChtZWFuID0gbWVhbiwgc2QgPSBzZCwgbWVkaWFuID0gbWVkaWFuLAogICAgICAgICAgICAgICAgICAgICAgICBzZSA9IH4gc2QoLngpIC8gc3FydChuKCkpCiAgICAgICAgICAgICAgICAgICAgICAgICkpLCAuZ3JvdXBzID0gJ2Ryb3AnKQoKaW50ZW5zaXR5X3N0YXRzICU+JQogIGthYmxlKCkKYGBgCgojIyMgQWdncmVnYXRlZCBub3JtYWxpemVkIG1lYW5zCgpRdWljayBzdW1tYXJ5IG9mIGBub3JtYWxpemVkIG1lYW5gIGZyb20gYWJvdmUKZXhwcmVzc2VkIGFzIGF2ZXJhZ2UgdmFsdWVzIG9mIHJlcGVhdC1sZXZlbCBtZWFucwp3aXRoIDk1JSBjb25maWRlbmNlIGludGVydmFsczoKCmBgYHtyfQptZWFuX2NpIDwtIGludGVuc2l0eV9zdGF0cyAlPiUKICBncm91cF9ieShsb2NhbGl6YXRpb24sIHN0cmFpbiklPiUKICBzdW1tYXJpc2Uocm91bmQobWVhbl9jbF9ub3JtYWwobm9ybWFsaXplZF9tZWFuKSwgMikpICU+JQogIHJlbmFtZShtZWFuID0geSwgbG93ZXIgPSB5bWluLCB1cHBlciA9IHltYXgpCgprYWJsZShtZWFuX2NpKQpgYGAKClRoaXMgaGludHMgdGhhdCB0aGUgdHJ1ZSBtZWFuIG9mIGN5dG9zb2xpYyBmbHVvcmVzY2VuY2UgCmlzIHVuY2hhbmdlZCBpbiBkaWZmZXJlbnQgc3RyYWlucywKYW5kIHRoYXQgM86UIGFuZCBvdmVyZXhwcmVzc2lvbiBzdHJhaW5zCmZlYXR1cmUgc29tZSAoc2duaWZpY2FudD8pIGxldmVscyBvZiBvdmVyZXhwcmVzc2lvbi4KTW9yZSBhY2N1cmF0ZSBlc3RpbWF0ZXMgYW5kIHAtdmFsdWVzIGNhbiBiZSBmb3VuZCBpbiB0aGUgJ01vZGVsaW5nJyB0YWIuCgojIyBQbG90cyAKCkkgaGF2ZSBjaG9zZW4gdG8gc2hvdyB0aGlzIGRhdGEgdXNpbmcgdGhlIApbU3VwZXJQbG90XShodHRwczovL2RvaS5vcmcvMTAuMTA4My9qY2IuMjAyMDAxMDY0KSBzdHlsZS4KRWFjaCBwb2ludCBzaG93cyBtZWFuIGludGVuc2l0eSBvZiBhIHNtYWxsIHJlZ2lvbiBpbiB0aGUgY3l0b3BsYXNtCm9mIGFuIGluZGl2aWR1YWwgY2VsbCwgb3Igb25lIGVudGlyZSBjZWxsIChzZXBhcmF0ZWQgaW50byBmYWNldHMpLgoKQmlnIGNvbG91ciBwb2ludHMgc2hvdyBtZWFuIG1lYXN1cmVtZW50cyBmcm9tIHRocmVlIGluZGVwZW5kZW50IHJlcGVhdHMuCgpSYW5nZSBpcyBtZWFuICsvLSBTRCwgY2FsY3VsYXRlZCBiYXNlZCBvbiB0aGUgdGhyZWUgaW5kZXBlbmRlbnQgcmVwZWF0cy4KCiMjIyBXaXRob3V0IG5vcm1hbGl6YXRpb24KClRoZXNlIHBsb3RzIHNob3cgcmF3IGludGVuc2l0eSBtZWFzdXJlcy4gVGhlIGRhdGFzZXQgZWZmZWN0IGlzIG5vdCBzbyBiYWQKaW4gdGhlIENlbGx1bGFyIGRhdGEsIHdoaWNoIHdlcmUgaW1hZ2VkIDMgZGF5cyBpbiBhIHJvdy4gSXQgaXMgcXVpdGUgcHJvbm91bmNlZAppbiB0aGUgQ3l0b3NvbGljIGRhdGEsIHdoZXJlIHRoZSBmYWNpbGl0eSByZWFsaWduZWQgdGhlIGxhc2VyIGZpYmVyIGluIHRoZSBtaWNyb3Njb3BlIApiZXR3ZWVuIGRhdGFzZXRzIDIgYW5kIDMsIGFmZmVjdGluZyB0aGUgYWJzb2x1dGUgdmFsdWVzLgoKQWxzbywgaWYgaXQgc2VlbXMgd2VpcmQgdGhhdCB0aGUgY3l0b3NvbGljIHZhbHVlcyBhcmUgaGlnaGVyCnRoYW4gdGhlIGNlbGx1bGFyIG9uZXMsIHRoYXQgaXMgbm90IGFjY2lkZW50YWwuIFRoZSBleHBvc3VyZSBvZiB0aGUgY2VsbHVsYXIKZGF0YSBoYWQgdG8gYmUga2VwdCBsb3cgaW4gb3JkZXIgdG8gYXZvaWQgc2F0dXJhdGluZyB0aGUgY29uZGVuc2F0ZXMuCkluIHRoZSBjeXRvc29saWMgZGF0YSwgdGhlIGV4cG9zdXJlcyB3ZXJlIGhpZ2ggaW4gb3JkZXIgdG8gbWF4aW1pemUKdGhlIGN5dG9zb2xpYyBzaWduYWwsIGFsbG93aW5nIGZvciB0aGUgY29uZGVuc2F0ZXMgdG8gc2F0dXJhdGUuCgpgYGB7cn0KcGxvdF9iYXNlIDwtIGdncGxvdChpbnRlbnNpdHlfc3RhdHMsCiAgICAgICAgICAgICAgICAgICBhZXMoeCA9IHN0cmFpbiwgeSA9IGludGVuc2l0eV9tZWFuKSkgKwogIGxhYnModGl0bGUgPSBOVUxMLCB4ID0gJ1N0cmFpbicsIHkgPSAnSW50ZW5zaXR5IChhLiB1LiknKSsKICBzY2FsZV95X2NvbnRpbnVvdXMoYnJlYWtzID0gc2NhbGVzOjpicmVha3NfZXh0ZW5kZWQoNikpKwogIHNjYWxlX3NoYXBlX21hbnVhbCh2YWx1ZXMgPSBjKDIxOjI1KSkgKwogIHNjYWxlX2NvbG9yX2JyZXdlcihwYWxldHRlID0gJ1NldDInKSArCiAgc2NhbGVfZmlsbF9icmV3ZXIocGFsZXR0ZSA9ICdTZXQyJykgKwogIGZhY2V0X3dyYXAoLiB+bG9jYWxpemF0aW9uLAogICAgICAgICAgICAgc2NhbGVzID0gJ2ZyZWUnKQoKCnBsb3Rfc2NhdHRlciA8LSBwbG90X2Jhc2UgKyAKICBnZW9tX3F1YXNpcmFuZG9tKGluaGVyaXQuYWVzID0gRiwgZGF0YSA9IGludGVuc2l0aWVzLAogICAgICAgICAgICAgICAgICAgYWVzKHggPSBzdHJhaW4sIHkgPSBpbnRlbnNpdHksCiAgICAgICAgICAgICAgICAgICAgICAgc2hhcGUgPSBkYXRhc2V0KSwKICAgICAgICAgICAgICAgICAgIGNvbG91ciA9ICdncmF5NzUnLCAjd2lkdGggPSAwLjMsCiAgICAgICAgICAgICAgICAgICBzaXplID0gMC44LCAjdmFyd2lkdGggPSBUUlVFLAogICAgICAgICAgICAgICAgICAgc2hvdy5sZWdlbmQgPSBGCiAgICAgICAgICAgICAgICAgICApCgpwbG90X3Zpb2xpbiA8LSBwbG90X2Jhc2UgKyAKICBnZW9tX3Zpb2xpbihpbmhlcml0LmFlcyA9IEYsIGRhdGEgPSBpbnRlbnNpdGllcywKICAgICAgICAgICAgICBhZXMoeCA9IHN0cmFpbiwgeSA9IGludGVuc2l0eSksCiAgICAgICAgICAgICAgc2NhbGUgPSAnd2lkdGgnLAogICAgICAgICAgICAgIGNvbG91ciA9ICdncmF5NzUnLCBmaWxsID0gJ3RyYW5zcGFyZW50JwogICAgICAgICAgICAgICkKYGBgCgpgYGB7cn0KcGxvdF9zdXBlciA8LSBwbG90X3NjYXR0ZXIgKwogIGdlb21fcXVhc2lyYW5kb20oYWVzKHNoYXBlID0gZGF0YXNldCwgZmlsbCA9IGRhdGFzZXQpLAogICAgICAgICAgICAgICAgICAgc2hvdy5sZWdlbmQgPSBGLCB3aWR0aCA9IDAuMywgc2l6ZSA9IDIpICsKICBzdGF0X3N1bW1hcnkoZnVuID0gbWVhbiwgZ2VvbSA9ICJjcm9zc2JhciIsIAogICAgICAgICAgICAgICB3aWR0aCA9IDAuNSwgZmF0dGVuID0gMSkgKwogIHN0YXRfc3VtbWFyeShmdW4uZGF0YSA9ICdtZWFuX3NkbCcsIGdlb20gPSAnZXJyb3JiYXInLAogICAgICAgICAgICAgICBmdW4uYXJncyA9IGxpc3QobXVsdCA9IDEpLCB3aWR0aCA9IDAuMikKcHJpbnQocGxvdF9zdXBlcikKbXlfZ2dzYXZlKCdmaWd1cmVzL3N1cGVyX3Jhdy5wZGYnKQpgYGAKCmBgYHtyfQojIFRoZXNlIHNpZ25pZmljYW5jZSBiYXJzIGFyZSBnZW5lcmF0ZWQgYnkgYGdnc2lnbmlmYCBmcm9tIHBhaXJ3aXNlIHQtdGVzdHMuCiMgVGhleSBhcmUgbm90IGVudGlyZWx5IGNvcnJlY3QgYW5kIHNvIHRoZXkgYXJlIG5vdCBzaG93biBpbiB0aGUgbm90ZWJvb2suCiMgVGhlIGZpZ3VyZSBpcyBzYXZlZCBmb3IgbWUsIHRvIG1hbnVhbGx5IGNoYW5nZSB0aGUgYW5ub3RhdGlvbnMKIyBpbiBJbGx1c3RyYXRvciB0byB0aGUgb25lcyBmcm9tIG11bHRpcGxlIGNvbXBhcmlzb25zIHRlc3RzLgoKcGxvdF9zaWduaWYgPC0gcGxvdF9zdXBlciArIAogIGdlb21fc2lnbmlmKGNvbXBhcmlzb25zID0gbGlzdChjKCJ3dCIsICIzzpQiKSwKICAgICAgICAgICAgICAgICAgICAgICAgICAgICAgICAgYygiM86UIiwgIm9lIiksCiAgICAgICAgICAgICAgICAgICAgICAgICAgICAgICAgIGMoInd0IiwgIm9lIikpLAogICAgICAgICAgICAgIG1hcF9zaWduaWZfbGV2ZWwgPSBULCB0ZXN0ID0gJ3QudGVzdCcsCiAgICAgICAgICAgICAgc3RlcF9pbmNyZWFzZSA9IDAuMDMsIHRpcF9sZW5ndGggPSAwLjAxLCB2anVzdCA9IDAuOCkKCm15X2dnc2F2ZSgnZmlndXJlcy9zdXBlcl9yYXdfc2lnbmlmLnBkZicsIHBsb3QgPSBwbG90X3NpZ25pZikKYGBgCgoKYGBge3J9CnBsb3Rfc3VwZXJfdmlvbGluIDwtIHBsb3RfdmlvbGluICsKICBnZW9tX3F1YXNpcmFuZG9tKGFlcyhzaGFwZSA9IGRhdGFzZXQsIGZpbGwgPSBkYXRhc2V0KSwKICAgICAgICAgICAgICAgICAgIHNob3cubGVnZW5kID0gRiwgd2lkdGggPSAwLjMsIHNpemUgPSAyKSArCiAgc3RhdF9zdW1tYXJ5KGZ1biA9IG1lYW4sIGdlb20gPSAiY3Jvc3NiYXIiLCAKICAgICAgICAgICAgICAgd2lkdGggPSAwLjUsIGZhdHRlbiA9IDEpICsKICBzdGF0X3N1bW1hcnkoZnVuLmRhdGEgPSAnbWVhbl9zZGwnLCBnZW9tID0gJ2Vycm9yYmFyJywKICAgICAgICAgICAgICAgZnVuLmFyZ3MgPSBsaXN0KG11bHQgPSAxKSwgd2lkdGggPSAwLjIpCiNwcmludChwbG90X3N1cGVyX3Zpb2xpbikKbXlfZ2dzYXZlKCdmaWd1cmVzL3N1cGVyX3Jhd192aW9saW4ucGRmJykKYGBgCgojIyMgTm9ybWFsaXplZAoKQWxsIG9ic2VydmF0aW9ucyBoYXZlIGJlZW4gc2NhbGVkIAp0byB0aGUgbWVhbiB2YWx1ZSBvZiB0aGUgd2lsZC10eXBlIHN0cmFpbiBvZiBlYWNoIGRhdGFzZXQuClRoaXMgbG9va3MgZ29vZCwgYnV0IGhhcyB0aGUgdW5mb3J0dW5hdGUgc2lkZS1lZmZlY3QKb2YgemVyb2luZyB0aGUgdmFyaWFuY2Ugb2YgdGhlIGRhdGFzZXQgbWVhbnMKaW4gdGhlIHdpbGQtdHlwZSBzdHJhaW4uCgpUaGlzIGJyZWFrcyBhbGwgdGhlIGFzc3VtcHRpb25zIG9mIEFOT1ZBIAppZiB3ZSB3YW50ZWQgdG8gcnVuIGl0IG9uIHJlcGVhdC1sZXZlbCBtZWFucy4KSXQgd2lsbCBiZSBiZXR0ZXIgdG8gc2hvdyB0aGUgcmF3IGRhdGEsCmFuZCBtYWtlIGEgbGluZWFyIG1vZGVsIHdoaWNoIGFjY291bnRzIGZvciBiYXRjaCBlZmZlY3RzCnRvIGdldCBlc3RpbWF0ZWQgbWVhbnMsIGVmZmVjdCBzaXplcyBhbmQgcC12YWx1ZXMuCgpgYGB7cn0KcGxvdF9iYXNlIDwtIGdncGxvdChpbnRlbnNpdHlfc3RhdHMsCiAgICAgICAgICAgICAgICAgICBhZXMoeCA9IHN0cmFpbiwgeSA9IG5vcm1hbGl6ZWRfbWVhbikpICsKICBsYWJzKHRpdGxlID0gTlVMTCwgeCA9ICdTdHJhaW4nLCB5ID0gJ05vcm1hbGl6ZWQgaW50ZW5zaXR5IChhLiB1LiknKSsKICBzY2FsZV95X2NvbnRpbnVvdXMoYnJlYWtzID0gc2NhbGVzOjpicmVha3NfZXh0ZW5kZWQoNikpKwogIHNjYWxlX3NoYXBlX21hbnVhbCh2YWx1ZXMgPSBjKDIxOjI1KSkgKwogIHNjYWxlX2NvbG9yX2JyZXdlcihwYWxldHRlID0gJ1NldDInKSArCiAgc2NhbGVfZmlsbF9icmV3ZXIocGFsZXR0ZSA9ICdTZXQyJykgKwogIGZhY2V0X3dyYXAoLiB+bG9jYWxpemF0aW9uLAogICAgICAgICAgICAgc2NhbGVzID0gJ2ZyZWUnKQoKCnBsb3Rfc2NhdHRlciA8LSBwbG90X2Jhc2UgKyAKICBnZW9tX3F1YXNpcmFuZG9tKGluaGVyaXQuYWVzID0gRiwgZGF0YSA9IGludGVuc2l0aWVzLAogICAgICAgICAgICAgICAgICAgYWVzKHggPSBzdHJhaW4sIHkgPSBub3JtYWxpemVkLAogICAgICAgICAgICAgICAgICAgICAgIHNoYXBlID0gZGF0YXNldCksCiAgICAgICAgICAgICAgICAgICBjb2xvdXIgPSAnZ3JheTc1JywgI3dpZHRoID0gMC4zLAogICAgICAgICAgICAgICAgICAgc2l6ZSA9IDAuOCwgI3ZhcndpZHRoID0gVFJVRSwKICAgICAgICAgICAgICAgICAgIHNob3cubGVnZW5kID0gRgogICAgICAgICAgICAgICAgICAgKQoKCnBsb3RfdmlvbGluIDwtIHBsb3RfYmFzZSArIAogIGdlb21fdmlvbGluKGluaGVyaXQuYWVzID0gRiwgZGF0YSA9IGludGVuc2l0aWVzLAogICAgICAgICAgICAgIGFlcyh4ID0gc3RyYWluLCB5ID0gbm9ybWFsaXplZCksCiAgICAgICAgICAgICAgc2NhbGUgPSAnd2lkdGgnLAogICAgICAgICAgICAgIGNvbG91ciA9ICdncmF5NzUnLCBmaWxsID0gJ3RyYW5zcGFyZW50JwogICAgICAgICAgICAgICkKYGBgCgpgYGB7cn0KcGxvdF9zdXBlciA8LSBwbG90X3NjYXR0ZXIgKwogIGdlb21fcXVhc2lyYW5kb20oYWVzKHNoYXBlID0gZGF0YXNldCwgZmlsbCA9IGRhdGFzZXQpLAogICAgICAgICAgICAgICAgICAgc2hvdy5sZWdlbmQgPSBGLCB3aWR0aCA9IDAuMywgc2l6ZSA9IDIpICsKICBzdGF0X3N1bW1hcnkoZnVuID0gbWVhbiwgZ2VvbSA9ICJjcm9zc2JhciIsIAogICAgICAgICAgICAgICB3aWR0aCA9IDAuNSwgZmF0dGVuID0gMSkgKwogIHN0YXRfc3VtbWFyeShmdW4uZGF0YSA9ICdtZWFuX3NkbCcsIGdlb20gPSAnZXJyb3JiYXInLAogICAgICAgICAgICAgICBmdW4uYXJncyA9IGxpc3QobXVsdCA9IDEpLCB3aWR0aCA9IDAuMikKcHJpbnQocGxvdF9zdXBlcikKbXlfZ2dzYXZlKCdmaWd1cmVzL3N1cGVyX3NjYXR0ZXIucGRmJykKYGBgCgpgYGB7cn0KcGxvdF9zdXBlcl92aW9saW4gPC0gcGxvdF92aW9saW4gKwogIGdlb21fcXVhc2lyYW5kb20oYWVzKHNoYXBlID0gZGF0YXNldCwgZmlsbCA9IGRhdGFzZXQpLAogICAgICAgICAgICAgICAgICAgc2hvdy5sZWdlbmQgPSBGLCB3aWR0aCA9IDAuMywgc2l6ZSA9IDIpICsKICBzdGF0X3N1bW1hcnkoZnVuID0gbWVhbiwgZ2VvbSA9ICJjcm9zc2JhciIsIAogICAgICAgICAgICAgICB3aWR0aCA9IDAuNSwgZmF0dGVuID0gMSkgKwogIHN0YXRfc3VtbWFyeShmdW4uZGF0YSA9ICdtZWFuX3NkbCcsIGdlb20gPSAnZXJyb3JiYXInLAogICAgICAgICAgICAgICBmdW4uYXJncyA9IGxpc3QobXVsdCA9IDEpLCB3aWR0aCA9IDAuMikKI3ByaW50KHBsb3Rfc3VwZXJfdmlvbGluKQojbXlfZ2dzYXZlKCdmaWd1cmVzL3N1cGVyX3Zpb2xpbi5wZGYnKQpgYGAKCmBgYHtyfQojIFRoZXNlIHNpZ25pZmljYW5jZSBiYXJzIGFyZSBnZW5lcmF0ZWQgYnkgYGdnc2lnbmlmYCBmcm9tIHBhaXJ3aXNlIHQtdGVzdHMuCiMgVGhleSBhcmUgbm90IGVudGlyZWx5IGNvcnJlY3QgYW5kIHNvIHRoZXkgYXJlIG5vdCBzaG93biBpbiB0aGUgbm90ZWJvb2suCiMgVGhlIGZpZ3VyZSBpcyBzYXZlZCBmb3IgbWUsIHRvIG1hbnVhbGx5IGNoYW5nZSB0aGUgYW5ub3RhdGlvbnMKIyBpbiBJbGx1c3RyYXRvciB0byB0aGUgb25lcyBmcm9tIG11bHRpcGxlIGNvbXBhcmlzb25zIHRlc3RzLCBpZSBBTk9WQS4KCnBsb3Rfc2lnbmlmIDwtIHBsb3Rfc3VwZXIgKyAKICBnZW9tX3NpZ25pZihjb21wYXJpc29ucyA9IGxpc3QoYygid3QiLCAiM86UIiksCiAgICAgICAgICAgICAgICAgICAgICAgICAgICAgICAgIGMoIjPOlCIsICJvZSIpLAogICAgICAgICAgICAgICAgICAgICAgICAgICAgICAgICBjKCJ3dCIsICJvZSIpKSwKICAgICAgICAgICAgICBtYXBfc2lnbmlmX2xldmVsID0gVCwgdGVzdCA9ICd0LnRlc3QnLAogICAgICAgICAgICAgIHN0ZXBfaW5jcmVhc2UgPSAwLjAzLCB0aXBfbGVuZ3RoID0gMC4wMSwgdmp1c3QgPSAwLjgpCgpteV9nZ3NhdmUoJ2ZpZ3VyZXMvcGxvdF9zdXBlcl9zaWduaWYucGRmJywgcGxvdCA9IHBsb3Rfc2lnbmlmKQpgYGAKCiMjIE1vZGVsaW5nCgpXZSB3YW50IHRvIGZpbmQgb3V0IGhvdyB0aGUgbWVhbiBpbnRlbnNpdHkgdmFsdWVzIApvZiBjeXRvcGxhc20gb3IgZW50aXJlIGNlbGxzIApkZXBlbmQgb24gdGhlIHN0cmFpbiB1c2VkIAoobnVsbCBoeXBvdGhlc2lzIGlzIHRoYXQgdGhleSBkbyBub3QpLgoKSW4gb3RoZXIgZXhwZXJpbWVudHMgSSBmb2xsb3dlZCB0aGUgcmVjb21tZW5kYXRpb25zIG9mCltzZXZlcmFsXShodHRwczovL2RvaS5vcmcvMTAuMTA4My9qY2IuMjAyMDAxMDY0KSAKW3BhcGVyc10oaHR0cHM6Ly9kb2kub3JnLzEwLjEwOTEvbWJjLkUxNS0wMi0wMDc2KQphbmQgcGVyZm9ybWVkIGh5cG90aGVzaXMgdGVzdHMgb24gcmVwZWF0LWxldmVsIG1lYW5zLgoKVGhpcyBhcHByb2FjaCB0YWtlcyBiZXR3ZWVuLWV4cGVyaW1lbnQgdmFyaWFiaWxpdHkgaW50byBhY2NvdW50LAphbmQgYXZvaWRzIHAtdmFsdWUgaW5mbGF0aW9uLCB3aGVyZSB0aW55IGRpZmZlcmVuY2VzIAptaWdodCBlbmQgdXAgJ2hpZ2hseSBzaWduaWZpY2FudCcgZHVlIHRvIHRlc3RpbmcKYSBsYXJnZSBudW1iZXIgb2Ygb2JzZXJ2YXRpb25zIHdoaWNoIGFyZSBub3QgbmVjZXNzYXJpbHkgaW5kZXBlbmRlbnQuCgpCdXQgaXQncyBhbHNvICp2ZXJ5KiBjb25zZXJ2YXRpdmUgCmJlY2F1c2UgdXNpbmcgYSBzbWFsbCBOIG9mIHJlcGVhdHMgbWVhbnMgbG9zaW5nIHBvd2VyCndoZW4gY29tcGFyZWQgdG8gdGhlIGh1bmRyZWRzIG9mIGNlbGwtbGV2ZWwgb2JzZXJ2YXRpb25zLgoKIyMjIExpbmVhciBtaXhlZCBtb2RlbAoKQmVjYXVzZSB3ZSBoYXZlIGEgY29tcGxldGUgYmxvY2sgZGVzaWduIAooYWxsIHN0cmFpbnMgYXJlIHJlcHJlc2VudGVkIGluIGFsbCBiYXRjaGVzKSwKd2UgY2FuIGluY2x1ZGUgdGhlIGRhdGFzZXQgYXMgYSBmaXhlZCBvciByYW5kb20gZWZmZWN0LgpTdHJhaW4gaXMgb2J2aW91c2x5IHRoZSBmaXhlZCBlZmZlY3Qgd2Ugd2FudCB0byBhbmFseXplLgpCZWNhdXNlIHdlIGRvIG5vdCBjYXJlIGFib3V0IGVzdGltYXRpbmcgdGhlIGVmZmVjdApvZiBhbnkgcGFydGljdWxhciBiYXRjaCwgYSBtaXhlZCBtb2RlbCB3aXRoIHRoZSBkYXRhc2V0CmFzIGEgcmFuZG9tIGVmZmVjdCB3aWxsIGJlIGFwcHJvcHJpYXRlLgoKIyMjIyBDZWxsdWxhcgoKYGBge3J9CmNlbGxfZGF0YSA8LSBpbnRlbnNpdGllcyAlPiUKICBmaWx0ZXIobG9jYWxpemF0aW9uID09ICdDZWxsdWxhcicpCmBgYAoKRml0dGluZyBhIGxpbmVhciBtaXhlZCBtb2RlbCB1c2luZyB0aGUgYGxtZXJUZXN0YCBwYWNrYWdlCihgbG1lcmAgY29tZXMgZnJvbSBgbG1lNGAsIGBsbWVyVGVzdGAganVzdCBhZHMgYW4gaW50ZXJmYWNlIGZvciBjb21wYXJpbmcgbW9kZWxzKToKCmBgYHtyfQpjZWxsX2xtbSA8LSBsbWVyKGludGVuc2l0eSB+IHN0cmFpbiArICgxfGRhdGFzZXQpLCBkYXRhID0gY2VsbF9kYXRhKQpzdW1tYXJ5KGNlbGxfbG1tKQpgYGAKCldlIGhhdmUgc2lnbmlmaWNhbnQgZWZmZWN0IG9mIGBzdHJhaW5gIG9uIGludGVuc2l0eQphY2NvcmRpbmcgdG8gYW4gRi10ZXN0OgoKYGBge3J9CmFub3ZhKGNlbGxfbG1tKQpgYGAKCmBkYXRhc2V0YCBkb2VzIG5vdCBzZWVtIHRvIGV4cGxhaW4gbXVjaCB2YXJpYW5jZSwKYnV0IGl0IGRlZmluaXRlbHkgKmNvdWxkKiAKZHVlIHRvIHRoZSBuYXR1cmUgb2YgbWVhc3VyaW5nIGFic29sdXRlIGZsdW9yZXNjZW5jZSB2YWx1ZXMsCmFzIHdlIHNlZSB3aXRoIHRoZSBDeXRvc29saWMgZGF0YS4KSXQgbWFrZXMgc2Vuc2UgdG8ga2VlcCBpdCBpbiB0aGUgbW9kZWwuCgpJbiB0aGUgcGxvdHMgd2Ugc2F3IHRoYXQgbWF5YmUgdGhlIGRhdGEgaXMgbm90IHJlYWxseSBub3JtYWwsCmFzIGlzIHR5cGljYWwgZm9yIGZsdW9yZXNjZW5jZSB2YWx1ZXMuCk1vZGVsIHJlc2lkdWFscyBhcmUgYWxzbyBub3Qgbm9ybWFsOgoKYGBge3J9CnFxbm9ybShyZXNpZChjZWxsX2xtbSksIG1haW4gPSAnTE1NIHJlc2lkdWFscyBRLVEgcGxvdCcpCnFxbGluZShyZXNpZChjZWxsX2xtbSkpCmhpc3QocmVzaWQoY2VsbF9sbW0pLCBicmVha3MgPSAxMDAsIHlsYWIgPSAnRnJlcXVlbmN5JywgeGxhYiA9ICdSZXNpZHVhbHMnLCBtYWluID0gJ0xNTSByZXNpZHVhbHMgaGlzdG9ncmFtJykKYGBgCgpUaGlzIGlzIGEgKmJpZyogZGVwYXJ0dXJlIGZyb20gbm9ybWFsaXR5LCAKd2l0aCBhIHN0cm9uZyBza2V3LAphbHRob3VnaCBpdCBsb29rcyBiZXR0ZXIgb24gdGhlIGhpc3RvZ3JhbQpNb3JlIGltcG9ydGFudGx5LCB0aGUgdmFyaWFuY2UgaW4gdGhlIGRhdGEgd2FzIGFsc28gbm90IGhvbW9nZW5lb3VzCihiaWdnZXIgc3ByZWFkIGluIE9FIGludGVuc2l0aWVzKS4KV2UgY2FuIHNlZSB0aGlzIHJlZmxlY3RlZCBpbiB0aGUgcmVzaWR1YWxzOgoKYGBge3J9CnBsb3QoY2VsbF9sbW0sIHlsYWIgPSAnUmVzaWR1YWxzJywgeGxhYiA9ICdGaXR0ZWQgdmFsdWVzJykKYGBgCgpUaGlzIGlzIHJlYWxseSBub3QgZ29vZCwgYnV0IHRoZW4gYWdhaW4gCnRoZSBjb25jbHVzaW9ucyBvZiB0aGUgZXhwZXJpbWVudCBhcmUgb2J2aW91cwphbmQgZG8gbm90IGhhbmcgb24gdGhlIHF1YWxpdHkgb2YgdGhlIG1vZGVsaW5nIGV4ZXJjaXNlLgpQcm9iYWJseSBsb2ctdHJhbnNmb3JtIG9yIGEgR0xNIGNvdWxkIGhlbHA7CmxldHMgc3RpY2sgdG8gdHJhbnNmb3JtaW5nIHRoZSBkYXRhLgoKV2Ugc3BlY2lmeSB0aGUgTE1NIHdpdGggYGxvZyhpbnRlbnNpdHkpIH4gc3RyYWluICsgKDF8ZGF0YXNldClgOgoKYGBge3J9CmNlbGxfbG9nX2xtbSA8LSBsbWVyKGxvZyhpbnRlbnNpdHkpIH4gc3RyYWluICsgKDF8ZGF0YXNldCksIGRhdGEgPSBjZWxsX2RhdGEpCnN1bW1hcnkoY2VsbF9sb2dfbG1tKQpgYGAKCldlIGFnYWluIGhhdmUgc3Ryb25nIGVmZmVjdCBvZiBgc3RyYWluYCBhbmQgYSByYW5kb20gZWZmZWN0CndpdGggdmVyeSBsaXR0bGUgZXhwbGFuYXRvcnkgcG93ZXIgZm9yIGBkYXRhc2V0YC4KTGV0J3MgbG9vayBhdCBRLVEgcGxvdCBhbmQgaGlzdG9ncmFtIG9mIG1vZGVsIHJlc2lkdWFsczoKCmBgYHtyfQpxcW5vcm0ocmVzaWQoY2VsbF9sb2dfbG1tKSkKcXFsaW5lKHJlc2lkKGNlbGxfbG9nX2xtbSkpCmhpc3QocmVzaWQoY2VsbF9sb2dfbG1tKSwgYnJlYWtzID0gMTAwKQpgYGAKClRoZSByZXNpZHVhbCBkaXN0cmlidXRpb24gaXMgc3RpbGwgZmF0LXRhaWxlZCAKYW5kIHNrZXdzIHNsaWdodGx5IGxlZnQgbm93LApidXQgdGhlIHRyYW5zZm9ybWF0aW9uIGhlbHBlZC4KTW9yZSBpbXBvcnRhbnRseSwgdGhlIGRpZmZlcmVuY2VzIGluIHNwcmVhZCBhbHNvIGRlY3JlYXNlZCwKYWx0aG91Z2ggdGhleSBhcmUgc3RpbGwgdGhlcmU6CgpgYGB7cn0KcGxvdChjZWxsX2xvZ19sbW0sIHlsYWIgPSAnUmVzaWR1YWxzJywgeGxhYiA9ICdGaXR0ZWQgdmFsdWVzJykKYGBgCgpXZSBjb3VsZCB0cnkgYSBnYW1tYSBHTE0sIGJ1dCBhZ2FpbiwKdGhlIGFic29sdXRlIHF1YWxpdHkgb2YgdGhlIG1vZGVsCmlzIG5vdCBjcml0aWNhbCB0byB0aGUgY29uY2x1c2lvbnMgaGVyZQpzbyBsZXQncyBub3Qgb3ZlciBjb21wbGljYXRlLgoKSSBzYXkgd2Ugc3RvcCBoZXJlIGFuZCBldmFsdWF0ZSB0aGUgZ3JvdXAgY29udHJhc3RzLgpUaGUgYGVtbWVhbnNgIHBhY2thZ2UgaGFzIGEgd29uZGVyZnVsIGludGVyZmFjZSBmb3IgcGFpcndpc2UgY29udHJhc3RzLAoqYW5kKiBpdCBjb21wdXRlcyBncm91cCBtZWFucyB3aXRoIGNvbmZpZGVuY2UgaW50ZXJ2YWxzLgoKYGBge3J9CmNlbGxfbG9nX2VtbSA8LSBlbW1lYW5zKGNlbGxfbG9nX2xtbSwgInN0cmFpbiIpCmBgYAoKVGhlIG1lYW5zIGFuZCBDSXMgYXJlIGNvbXB1dGVkIGluIGxvZy1zcGFjZSBhbmQgYmFjay10cmFuc2Zvcm1lZCwKZ2l2aW5nIHRoZSBhYnNvbHV0ZSBmbHVvcmVzY2VuY2UgdmFsdWVzOgoKYGBge3J9CnN1bW1hcnkoY2VsbF9sb2dfZW1tLCB0eXBlID0gJ3Jlc3BvbnNlJykgJT4lCiAgdGlkeSgpICU+JQogIGthYmxlKCkKYGBgCgpXZSBjYW4gYWxzbyBjb21wdXRlIGRpZmZlcmVuY2VzIGJldHdlZW4gZ3JvdXBzIGFsb25nIHdpdGggdGhlaXIgQ0lzCmFuZCBwLXZhbHVlcyBmb3IgcGFpcndpc2UgY29tcGFyaXNvbnMuCkJlY2F1c2UgdGhlIGRpZmZlcmVuY2VzIGFyZSBjb21wdXRlZCBpbiBsb2ctc3BhY2UsCndlIGdldCByYXRpb3MgYWZ0ZXIgYmFjay10cmFuc2Zvcm1pbmcuClRoaXMgaXMgYWN0dWFsbHkgdmVyeSB1c2VmdWwKYmVjYXVzZSB0aGUgcmF0aW8gdG8gd2lsZC10eXBlCmlzIHdoYXQgd2Ugd2FudGVkIHRvIGtub3cgaW4gdGhlIGZpcnN0IHBsYWNlLgoKYGBge3J9CnN1bW1hcnkoY29udHJhc3QoY2VsbF9sb2dfZW1tLCB0eXBlID0gJ3Jlc3BvbnNlJywgbWV0aG9kID0gJ3JldnBhaXJ3aXNlJyksCiAgICAgICAgaW5mZXIgPSBUKSAlPiUKICBrYWJsZSgpCmBgYAoKRmluYWxseSwgdGhlIGBlbW1lYW5zYCBpbnRlcmZhY2UgYWxsb3dzIHVzIHRvIHBsb3QKdGhlIG1vZGVsZWQgbWVhbnMgYWxvbmcgd2l0aCBjb25maWRlbmNlIGludGVydmFscyAoYmFycykKYW5kIGNvbmZpZGVuY2UgaW50ZXJ2YWxzIGZvciB0aGUgZ3JvdXAgZGlmZmVyZW5jZXMgKGFycm93cykuCgpgYGB7cn0KcGxvdChjZWxsX2xvZ19lbW0sIGNvbXBhcmlzb25zID0gVCwgdHlwZSA9ICdyZXNwb25zZScsIAogICAgIHhsYWIgPSAnTWVhbiBmbHVvcmVzY2VuY2UgKy8tIDk1JSBDSSAoYS51LiknKQpgYGAKCldlIGNhbiBjb25jbHVkZSB0aGF0IGFsbCBncm91cHMKYXJlIHNpZ25pZmljYW50bHkgZGlmZmVyZW50LCAKd2l0aCBleHRyZW1lbHkgbG93IHAtdmFsdWVzIChub3QgdGhhdCBpdCBtYXR0ZXJzKS4KM+KIhiBiYWNrZ3JvdW5kIGluY3JlYXNlcyBFZGUxIGxldmVscyBieSBhIG1vZGVzdCB+MjAlLAphbmQgQURIMS1kcml2ZW4gb3ZlcmV4cHJlc3Npb24gYnkgfjIwMCUuCgojIyMjIEN5dG9zb2xpYwoKYGBge3J9CmN5dG9fZGF0YSA8LSBpbnRlbnNpdGllcyAlPiUKICBmaWx0ZXIobG9jYWxpemF0aW9uID09ICdDeXRvc29saWMnKQpgYGAKCkFmdGVyIGxvb2tpbmcgYXQgdGhlIHBsb3RzLCAKd2UgZ28gaW50byB0aGlzIGV4ZXJjaXNlIGFscmVhZHkgc3VzcGVjdGluZyAKdGhhdCBgc3RyYWluYCBoYXMgbm8gZWZmZWN0LiBCdXQgbGV0J3MgYnVpbGQgYSBtb2RlbCBhcyBiZWZvcmU6CgpgYGB7cn0KY3l0b19sbW0gPC0gbG1lcihpbnRlbnNpdHkgfiBzdHJhaW4gKyAoMXxkYXRhc2V0KSwgZGF0YSA9IGN5dG9fZGF0YSkKc3VtbWFyeShjeXRvX2xtbSkKYGBgCgpBZnRlciBtb2RlbGluZyB0aGUgZml4ZWQgZWZmZWN0IG9mIGBzdHJhaW5gLApkYXRhc2V0IGV4cGxhaW5zIH4yNSUgb2YgdGhlIHZhcmlhbmNlLgpgc3RyYWluYCBpdHNlbGYgaXMgbm90IGEgc2lnbmlmaWNhbnQgcHJlZGljdG9yOgoKYGBge3J9CmFub3ZhKGN5dG9fbG1tKQpgYGAKCldlIHJlYWxseSBjb3VsZCBzdG9wIGhlcmUsIHNpbmNlIGBzdHJhaW5gIGRvZXMgbm90IGV4cGxhaW4gYW55dGhpbmcsCmFuZCB0aGF0J3MgYXMgY2xlYXIgZnJvbSB0aGUgbW9kZWwgYXMgZnJvbSB0aGUgcGxvdHMuCkJ1dCBsZXQncyBjaGVjayBtb2RlbCByZXNpZHVhbHM6CgpgYGB7cn0KcXFub3JtKHJlc2lkKGN5dG9fbG1tKSwgbWFpbiA9ICdDeXRvc29saWMgTE1NIFEtUSBwbG90JykKcXFsaW5lKHJlc2lkKGN5dG9fbG1tKSkKcGxvdChjeXRvX2xtbSwgeWxhYiA9ICdSZXNpZHVhbHMnLCB4bGFiID0gJ0ZpdHRlZCB2YWx1ZXMnKQpgYGAKClRoaXMgaXMgbm90IHNvIGJhZCBhY3R1YWxseSBidXQgd2UgaGF2ZSBzb21lIGZhdCB0YWlscywKYW5kIGEgc21hbGwgZGlmZmVyZW5jZSBpbiBzcHJlYWQgZm9yIGxhcmdlciB2YWx1ZXMuCkxvZy10cmFuc2Zvcm0gaGVscGVkIGJlZm9yZSBzbyBsZXQncyBzZWUgd2hhdCBpdCBkb2VzIGhlcmU6CgpgYGB7cn0KY3l0b19sb2dfbG1tIDwtIGxtZXIobG9nKGludGVuc2l0eSkgfiBzdHJhaW4gKyAoMXxkYXRhc2V0KSwgZGF0YSA9IGN5dG9fZGF0YSkKc3VtbWFyeShjeXRvX2xvZ19sbW0pCmBgYAoKVGhpcyBpcyBiZXR0ZXIsIGVycm9yLXdpc2U6CgpgYGB7cn0KcXFub3JtKHJlc2lkKGN5dG9fbG9nX2xtbSksIG1haW4gPSAnQ3l0b3NvbGljIGxvZ19sbW0gUS1RIHBsb3QnKQpxcWxpbmUocmVzaWQoY3l0b19sb2dfbG1tKSkKcGxvdChjeXRvX2xvZ19sbW0sIHlsYWIgPSAnUmVzaWR1YWxzJywgeGxhYiA9ICdGaXR0ZWQgdmFsdWVzJykKYGBgCgpCdXQgYHN0cmFpbmAgaXMgc3RpbGwgbm90IHNpZ25pZmljYW50OgoKYGBge3J9CmFub3ZhKGN5dG9fbG9nX2xtbSkKYGBgCgpBbmQgaWYgd2UgdXNlIGBlbW1lYW5zYCB0byBnZXQgY29udHJhc3RzLAp3ZSBmaW5kIG5vIHNpZ25pZmljYW50IGRpZmZlcmVuY2VzIGJldHdlZW4gZ3JvdXBzOgoKYGBge3J9CmN5dG9fbG9nX2VtbSA8LSBlbW1lYW5zKGN5dG9fbG9nX2xtbSwgInN0cmFpbiIpCnN1bW1hcnkoY29udHJhc3QoY3l0b19sb2dfZW1tLCB0eXBlID0gJ3Jlc3BvbnNlJywgbWV0aG9kID0gJ3JldnBhaXJ3aXNlJyksCiAgICAgICAgaW5mZXIgPSBUKSAlPiUKICBrYWJsZSgpCmBgYAoKU2luY2UgZmx1b3Jlc2NlbmNlIGludGVuc2l0eSB2YWx1ZXMgYXJlIG5vdCBtZWFuaW5nZnVsLApJIGFtIHNraXBwaW5nIHRoZSBlc3RpbWF0ZWQgZ3JvdXAgbWVhbnMgaGVyZS4KCiMjIENvbmNsdXNpb25zCgoxLiBXZSBjYW5ub3QgZGV0ZWN0IGFueSBkaWZmZXJlbmNlcyAKICAgIGluIGN5dG9zb2xpYyBFZGUxIGludGVuc2l0aWVzIAogICAgYWNyb3NzIGRpZmZlcmVudCBzdHJhaW5zLgoKMi4gV2Ugc2VlIHNpZ25pZmljYW50IGRpZmZlcmVuY2VzIGluIG1lYW4gdG90YWwgY2VsbHVsYXIgbGV2ZWxzIG9mIEVkZTEsIG5hbWVseToKICAgICogfjEyMCUgb2Ygd2lsZC10eXBlIEVkZTEgbGV2ZWxzIGluIDPiiIYgc3RyYWlucywgYW5kCiAgICAqIH4zMjAlIG9mIHdpbGQtdHlwZSBFZGUxIGxldmVscyBpbiBBREgxLWRyaXZlbiBvdmVyZXhwcmVzc2lvbi4KCkEgdGFibGUgc3VtbWFyaXNpbmcgYWxsIGdyb3VwIGNvbnRyYXN0czoKCmBgYHtyfQp0aWJibGUoc3VtbWFyeShjb250cmFzdChjeXRvX2xvZ19lbW0sIHR5cGUgPSAncmVzcG9uc2UnLCBtZXRob2QgPSAncmV2cGFpcndpc2UnKSwKICAgICAgICBpbmZlciA9IFQpLCBsb2NhbGl6YXRpb24gPSAnQ3l0b3NvbGljJykgJT4lCiAgYmluZF9yb3dzKHRpYmJsZShzdW1tYXJ5KGNvbnRyYXN0KGNlbGxfbG9nX2VtbSwgdHlwZSA9ICdyZXNwb25zZScsIG1ldGhvZCA9ICdyZXZwYWlyd2lzZScpLAogICAgICAgIGluZmVyID0gVCksIGxvY2FsaXphdGlvbiA9ICdDZWxsdWxhcicpKSU+JQogIHNlbGVjdChjb250cmFzdCwgcmF0aW8sIGxvd2VyLkNMLCB1cHBlci5DTCwgbG9jYWxpemF0aW9uKSAlPiUKICBtdXRhdGUoYWNyb3NzKHdoZXJlKGlzLm51bWVyaWMpLCByb3VuZCwgZGlnaXRzID0gMikpICU+JQogIGthYmxlKCkKYGBgCgojIyBTb3VyY2UgZGF0YQoKIyMjIC5jc3YKCmBgYHtyIGVjaG89RkFMU0V9CnhmdW46OmVtYmVkX2ZpbGUoJ2RhdGEvaW50ZW5zaXRpZXMuY3N2JykKYGBgCgojIyMgLlJEYXRhCgpgYGB7ciBlY2hvPUZBTFNFfQp4ZnVuOjplbWJlZF9maWxlKCdkYXRhL2RhdGFfY2xlYW4uUkRhdGEnKQpgYGAKCiMjIFNlc3Npb24gaW5mbwoKUiBzZXNzaW9uIHVzZWQgdG8gZ2VuZXJhdGUgdGhpcyBkb2N1bWVudC4KCmBgYHtyIHNlc3Npb24sIG1lc3NhZ2U9VFJVRX0Kc2Vzc2lvbkluZm8oKQpgYGAK
