# Supplementary material for: Condensation of Ede1 promotes the initiation of endocytosis
Source: eLife. 2022 Apr 12;11:e72865. doi: 10.7554/eLife.72865 (PMC9064294; doi:10.7554/eLife.72865)
Supplement: Figure 3—source data 2. [file elife-72865-fig3-data2.zip › figure3_data2.html]

GALS induction of Ede1 expression


Code 

- Download Rmd

# GALS induction of Ede1 expression

#### Last compiled on July 28, 2021

# 

## Experiment

### Premise

The aim of this experiment is to probe the cytosolic concentration of Ede1 after induction of expression from the GALS promoter.

The prediction from a pure phase separation model based on homotypic interactions is that the cytosolic concentration would be buffered, as the dense and light phase concentrations are fixed, and only the volume fraction changes as total concentration increases.

The prediction from a pure scaffold-binding model is that light phase concentration can increase, and the size of the structures is limited by the availability of the scaffold.

Phase separation driven by heterotypic interactions would be more complex, as the phase separation would depend both on concentration and relative stoichiometry of phase-separating components.

### Yeast growth

Yeast strains MKY4405 (‘oe’, *GALSpr-EGFP-EDE1*) and MKY0379 (‘wt’, *EDE1-EGFP, SLA1-mCherry*) were grown on an SC-Gal plate. They were cultured overnight in a liquid SC-Trp medium with 2% Raffinose as the carbon source, diluted in the morning to OD600 = 0.2 in SC-Trp medium with 2% glucose as the carbon source and grown for ~4h. The two strains were mixed in equal amounts and attached to a coverslip. ~15 minutes before the start of the movies, the medium on the coverslip was exchanged to SC-Trp with 2% galactose as the carbon source.

### Imaging

The yeast were imaged on a Nikon Ti with Yokogawa CSW-1 spinning disk confocal. Both channels were acquired with single bandpass filters for their respective wavelengths (excitation using 488 and 561 nm lasers). Exposure time and laser power were equal for both (400 ms / 100%), with 5-minute intervals between frames. GFP signal was acquired with camera gain 1 and mCherry with gain 3. Perfect Focus system was used throughout the movie.

### Processing

The movies were first corrected for drift using the StackReg FIJI plugin from EPFL-BIG.

Regions of interest were selected manually and measured over the entire duration of the movies. Background and cytosolic intensities were measured over 5 px square regions. Care was taken to avoid condensates and, if possible, dark vacuolar regions in the cytosolic ROIs. The cellular signal was measured from oval ROIs on the same cells (these are single-plane movies).

Cell selection:

- wt => cells bearing both markers
- oe => cells having only GFP and showing condensates in the focal plane

Regions measured in a representative movie

## Results

### Raw plot

This plot shows three classes of signal intensity:

- background,
- EGFP-Ede1 in overexpressing cells, and
- Ede1-EGFP in wild-type cells.

Line is mean, shaded area is 95% confidence interval.

### Background corrected

Plot shows two classes of signal intensity:

- EGFP-Ede1 in overexpressing cells, and
- Ede1-EGFP in wild-type cells.

Both were corrected by subtracting the background value.

Line is mean +/- 2xSEM (95% confidence interval based on normal distribution); uncertainty of background measurements has been propagated during subtraction.

### Normalized

Plot shows cytosolic intensity of EGFP-Ede1 in overexpressing cells normalized in respect to the Ede1-EGFP cytosolic intensity in wild-type cells. Both were corrected prior to division by subtracting the background value.

Line is mean +/- 2xSEM (95% confidence interval based on normal distribution); errors from subtraction and division have been propagated.

## Source data

### .csv (raw intensities)

Download ede1\_gal\_induction.csv

### .RData (everything)

Download ede1\_gal\_induction.RData

LS0tCnRpdGxlOiAiR0FMUyBpbmR1Y3Rpb24gb2YgRWRlMSBleHByZXNzaW9uIgpkYXRlOiAiTGFzdCBjb21waWxlZCBvbiBgciBmb3JtYXQoU3lzLnRpbWUoKSwgJyVCICVkLCAlWScpYCIKb3V0cHV0OgogIGh0bWxfZG9jdW1lbnQ6CiAgICBjb2RlX2Rvd25sb2FkOiB0cnVlCi0tLQoKYGBge3Igc2V0dXAsIGluY2x1ZGU9RkFMU0V9CmtuaXRyOjpvcHRzX2NodW5rJHNldChlY2hvID0gRkFMU0UsIG1lc3NhZ2U9RkFMU0UsIHdhcm5pbmc9RkFMU0UsCiAgICAgICAgICAgICAgICAgICAgICBkcGkgPSA5NiwgZmlnLndpZHRoID0gNywgZmlnLmhlaWdodCA9IDUpCmBgYAoKYGBge3IgbGlic30KbGlicmFyeSh0aWR5dmVyc2UpICMgSSBtYWtlIGEgbG90IG9mIHVzZSBvZiBkcGx5ciBhbmQgb3RoZXIgdGlkeXZlcnNlIHBhY2thZ2VzCmBgYAoKYGBge3IgbG9hZH0Kcm0obGlzdCA9IGxzKCkpCmludGVuc2l0aWVzIDwtIHJlYWRfY3N2KCdkYXRhL2VkZTFfZ2FsX2luZHVjdGlvbi5jc3YnKSAKYGBgCgpgYGB7ciB0aGVtZX0KIyBDdXN0b20gZ2dwbG90MiB0aGVtZQojIC0tLS0tLS0tLS0tLS0tLS0tLS0tCgojIG1pbmltYWwgdGhlbWUgd2l0aCBib3JkZXIKIyBiYXNlZCBvbiB0aGVtZV9saW5lZHJhdyB3aXRob3V0IHRoZSBncmlkIGxpbmVzCiMgYWxzbyB0cnlpbmcgdG8gcmVtb3ZlIGFsbCBiYWNrZ3JvdW5kcyBhbmQgbWFyZ2lucwojIHRoZSBhaW0gaXMgdG8gbWFrZSBpdCBhcyBlYXN5IGFzIHBvc3NpYmxlIHRvIGVkaXQgaW4gaWxsdXN0cmF0b3IKCnRoZW1lX2ZyYXAgPC0gZnVuY3Rpb24oYmFzZV9zaXplID0gMTEsIGJhc2VfZmFtaWx5ID0gIiIsCiAgICAgICAgICAgICAgICAgICAgICAgIGJhc2VfbGluZV9zaXplID0gYmFzZV9zaXplIC8gMjIsCiAgICAgICAgICAgICAgICAgICAgICAgIGJhc2VfcmVjdF9zaXplID0gYmFzZV9zaXplIC8gMjIpIHsKICB0aGVtZV9saW5lZHJhdygKICAgIGJhc2Vfc2l6ZSA9IGJhc2Vfc2l6ZSwKICAgIGJhc2VfZmFtaWx5ID0gYmFzZV9mYW1pbHksCiAgICBiYXNlX2xpbmVfc2l6ZSA9IGJhc2VfbGluZV9zaXplLAogICAgYmFzZV9yZWN0X3NpemUgPSBiYXNlX3JlY3Rfc2l6ZQogICkgJStyZXBsYWNlJQogICAgdGhlbWUoCiAgICAgICMgbm8gZ3JpZCBhbmQgbm8gYmFja2dyb3VuZHMgaWYgSSBjYW4gaGVscCBpdAogICAgICBsZWdlbmQuYmFja2dyb3VuZCA9ICBlbGVtZW50X2JsYW5rKCksCiAgICAgIHBhbmVsLmJhY2tncm91bmQgPSBlbGVtZW50X2JsYW5rKCksCiAgICAgIHBhbmVsLmdyaWQgPSBlbGVtZW50X2JsYW5rKCksCiAgICAgIHBsb3QuYmFja2dyb3VuZCA9IGVsZW1lbnRfYmxhbmsoKSwKICAgICAgcGxvdC5tYXJnaW4gPSBtYXJnaW4oMCwgMCwgMCwgMCksCiAgICAgIGNvbXBsZXRlID0gVFJVRQogICAgKQp9CgojIFNldCBkZWZhdWx0IHRoZW1lCiMgLS0tLS0tLS0tLS0tLS0tLS0KdGhlbWVfc2V0KHRoZW1lX2ZyYXAoYmFzZV9zaXplID0gMTQsIGJhc2VfZmFtaWx5ID0gIk15cmlhZCBQcm8iKSkKCiMgQ3JlYXRlIGEgZ2dzYXZlIHdyYXBwZXIKIyAtLS0tLS0tLS0tLS0tLS0tLS0tLS0tLQoKIyBUaGlzIHdheSB3ZSBjYW4gc2V0IGEgZGVmYXVsdCBzaXplIGFuZCBkZXZpY2UgZm9yIGFsbCBwbG90cwpteV9nZ3NhdmUgPC0gZnVuY3Rpb24oZmlsZW5hbWUsIHBsb3QgPSBsYXN0X3Bsb3QoKSwKICAgICAgICAgICAgICAgICAgICAgIGRldmljZSA9IGNhaXJvX3BkZiwgdW5pdHMgPSAibW0iLAogICAgICAgICAgICAgICAgICAgICAgd2lkdGggPSAxMjAsIGhlaWdodCA9IDkwLCAuLi4pewogIGdnc2F2ZShmaWxlbmFtZSA9IGZpbGVuYW1lLCBwbG90ID0gcGxvdCwKICAgICAgICAgZGV2aWNlID0gZGV2aWNlLCB1bml0cyA9IHVuaXRzLAogICAgICAgICBoZWlnaHQgPSBoZWlnaHQsIHdpZHRoID0gd2lkdGgsICAuLi4pCiAgfQpgYGAKCiMgey50YWJzZXR9CgojIyBFeHBlcmltZW50CgojIyMgUHJlbWlzZQoKVGhlIGFpbSBvZiB0aGlzIGV4cGVyaW1lbnQgaXMgdG8gcHJvYmUgdGhlIGN5dG9zb2xpYyBjb25jZW50cmF0aW9uIG9mIEVkZTEKYWZ0ZXIgaW5kdWN0aW9uIG9mIGV4cHJlc3Npb24gZnJvbSB0aGUgR0FMUyBwcm9tb3Rlci4KClRoZSBwcmVkaWN0aW9uIGZyb20gYSBwdXJlIHBoYXNlIHNlcGFyYXRpb24gbW9kZWwgCmJhc2VkIG9uIGhvbW90eXBpYyBpbnRlcmFjdGlvbnMKaXMgdGhhdCB0aGUgY3l0b3NvbGljIGNvbmNlbnRyYXRpb24gd291bGQgYmUgYnVmZmVyZWQsIAphcyB0aGUgZGVuc2UgYW5kIGxpZ2h0IHBoYXNlIGNvbmNlbnRyYXRpb25zIGFyZSBmaXhlZCwgCmFuZCBvbmx5IHRoZSB2b2x1bWUgZnJhY3Rpb24gY2hhbmdlcyBhcyB0b3RhbCBjb25jZW50cmF0aW9uIGluY3JlYXNlcy4KClRoZSBwcmVkaWN0aW9uIGZyb20gYSBwdXJlIHNjYWZmb2xkLWJpbmRpbmcgbW9kZWwgCmlzIHRoYXQgbGlnaHQgcGhhc2UgY29uY2VudHJhdGlvbiBjYW4gaW5jcmVhc2UsIAphbmQgdGhlIHNpemUgb2YgdGhlIHN0cnVjdHVyZXMgaXMgbGltaXRlZCBieSB0aGUgYXZhaWxhYmlsaXR5IG9mIHRoZSBzY2FmZm9sZC4KClBoYXNlIHNlcGFyYXRpb24gZHJpdmVuIGJ5IGhldGVyb3R5cGljIGludGVyYWN0aW9ucwp3b3VsZCBiZSBtb3JlIGNvbXBsZXgsIGFzIHRoZSBwaGFzZSBzZXBhcmF0aW9uIHdvdWxkIGRlcGVuZApib3RoIG9uIGNvbmNlbnRyYXRpb24gYW5kIHJlbGF0aXZlIHN0b2ljaGlvbWV0cnkKb2YgcGhhc2Utc2VwYXJhdGluZyBjb21wb25lbnRzLgoKIyMjIFllYXN0IGdyb3d0aAoKWWVhc3Qgc3RyYWlucyBNS1k0NDA1ICgnb2UnLCAqR0FMU3ByLUVHRlAtRURFMSopCmFuZCBNS1kwMzc5ICgnd3QnLCAqRURFMS1FR0ZQLCBTTEExLW1DaGVycnkqKSAKd2VyZSBncm93biBvbiBhbiBTQy1HYWwgcGxhdGUuIApUaGV5IHdlcmUgY3VsdHVyZWQgb3Zlcm5pZ2h0IAppbiBhIGxpcXVpZCBTQy1UcnAgbWVkaXVtIHdpdGggMiUgUmFmZmlub3NlIGFzIHRoZSBjYXJib24gc291cmNlLCAKZGlsdXRlZCBpbiB0aGUgbW9ybmluZyB0byBPRDYwMCA9IDAuMiAKaW4gU0MtVHJwIG1lZGl1bSB3aXRoIDIlIGdsdWNvc2UgYXMgdGhlIGNhcmJvbiBzb3VyY2UgYW5kIGdyb3duIGZvciB+NGguIApUaGUgdHdvIHN0cmFpbnMgd2VyZSBtaXhlZCBpbiBlcXVhbCBhbW91bnRzIGFuZCBhdHRhY2hlZCB0byBhIGNvdmVyc2xpcC4KfjE1IG1pbnV0ZXMgYmVmb3JlIHRoZSBzdGFydCBvZiB0aGUgbW92aWVzLCAKdGhlIG1lZGl1bSBvbiB0aGUgY292ZXJzbGlwIHdhcyBleGNoYW5nZWQgCnRvIFNDLVRycCB3aXRoIDIlIGdhbGFjdG9zZSBhcyB0aGUgY2FyYm9uIHNvdXJjZS4KCiMjIyBJbWFnaW5nCgpUaGUgeWVhc3Qgd2VyZSBpbWFnZWQgb24gYSBOaWtvbiBUaSB3aXRoIFlva29nYXdhIENTVy0xIHNwaW5uaW5nIGRpc2sgY29uZm9jYWwuIApCb3RoIGNoYW5uZWxzIHdlcmUgYWNxdWlyZWQgd2l0aCBzaW5nbGUgYmFuZHBhc3MgZmlsdGVycyAKZm9yIHRoZWlyIHJlc3BlY3RpdmUgd2F2ZWxlbmd0aHMgKGV4Y2l0YXRpb24gdXNpbmcgNDg4IGFuZCA1NjEgbm0gbGFzZXJzKS4gCkV4cG9zdXJlIHRpbWUgYW5kIGxhc2VyIHBvd2VyIHdlcmUgZXF1YWwgZm9yIGJvdGggKDQwMCBtcyAvIDEwMCUpLCAKd2l0aCA1LW1pbnV0ZSBpbnRlcnZhbHMgYmV0d2VlbiBmcmFtZXMuCkdGUCBzaWduYWwgd2FzIGFjcXVpcmVkIHdpdGggY2FtZXJhIGdhaW4gMSBhbmQgbUNoZXJyeSB3aXRoIGdhaW4gMy4KUGVyZmVjdCBGb2N1cyBzeXN0ZW0gd2FzIHVzZWQgdGhyb3VnaG91dCB0aGUgbW92aWUuCgojIyMgUHJvY2Vzc2luZwoKVGhlIG1vdmllcyB3ZXJlIGZpcnN0IGNvcnJlY3RlZCBmb3IgZHJpZnQgCnVzaW5nIHRoZSBTdGFja1JlZyBGSUpJIHBsdWdpbiBmcm9tIEVQRkwtQklHLgoKUmVnaW9ucyBvZiBpbnRlcmVzdCB3ZXJlIHNlbGVjdGVkIG1hbnVhbGx5CmFuZCBtZWFzdXJlZCBvdmVyIHRoZSBlbnRpcmUgZHVyYXRpb24gb2YgdGhlIG1vdmllcy4KQmFja2dyb3VuZCBhbmQgY3l0b3NvbGljIGludGVuc2l0aWVzIHdlcmUgbWVhc3VyZWQgb3ZlciA1IHB4IHNxdWFyZSByZWdpb25zLgpDYXJlIHdhcyB0YWtlbiB0byBhdm9pZCBjb25kZW5zYXRlcyBhbmQsIGlmIHBvc3NpYmxlLCAKZGFyayB2YWN1b2xhciByZWdpb25zIGluIHRoZSBjeXRvc29saWMgUk9Jcy4KVGhlIGNlbGx1bGFyIHNpZ25hbCB3YXMgbWVhc3VyZWQgZnJvbSBvdmFsIFJPSXMgb24gdGhlIHNhbWUgY2VsbHMKKHRoZXNlIGFyZSBzaW5nbGUtcGxhbmUgbW92aWVzKS4KCkNlbGwgc2VsZWN0aW9uOgoKKiB3dCA9PiBjZWxscyBiZWFyaW5nIGJvdGggbWFya2VycwoqIG9lID0+IGNlbGxzIGhhdmluZyBvbmx5IEdGUCBhbmQgc2hvd2luZyBjb25kZW5zYXRlcyBpbiB0aGUgZm9jYWwgcGxhbmUKCiFbUmVnaW9ucyBtZWFzdXJlZCBpbiBhIHJlcHJlc2VudGF0aXZlIG1vdmllXShmaWd1cmVzL2NlbGxfcmVnaW9ucy5wbmcpCgojIyBSZXN1bHRzIHsudGFic2V0IC50YWJzZXQtcGlsbHN9CgpgYGB7ciBzdW1tYXJpc2V9Cm1lYW5faW50ZW5zaXRpZXMgPC0gaW50ZW5zaXRpZXMgJT4lCiAgZ3JvdXBfYnkodGltZSwgc2lnbmFsKSAlPiUKICAjIHNpZ25hbCB2YXJpYWJsZSBjb21iaW5lcyBzdHJhaW4gYW5kIGxvY2FsaXphdGlvbiBmb3Igbm93CiAgIyBnZW5lcmF0ZSBzdGF0aXN0aWNzIGZvciBlYWNoIHNpZ25hbCBhdCBlYWNoIHRpbWVwb2ludAogIHN1bW1hcmlzZShtZWFuID0gbWVhbihpbnRlbnNpdHkpLCBzZCA9IHNkKGludGVuc2l0eSksIG4gPSBuKCksIHNlID0gc2QoaW50ZW5zaXR5KSAvIHNxcnQobigpKSkgJT4lCiAgIyBnZW5lcmF0ZSB3aWRlIGZvcm1hdCBkYXRhOiBlYWNoIHN0YXRpc3RpYyBnZXRzIGl0cyBvd24gY29sdW1uIGZvciBlYWNoIHNpZ25hbAogIHBpdm90X3dpZGVyKG5hbWVzX2Zyb20gPSBzaWduYWwsIHZhbHVlc19mcm9tID0gYyhtZWFuLCBzZCwgc2UsIG4pKSAlPiUKICAjIHVzZSB0aGUgbmV3IGNvbHVtbnMgdG8gZ2VuZXJhdGUgYmFja2dyb3VuZC1zdWJ0cmFjdGVkIGNhbHVlcyAoeF9jb3JyKQogICMgYW5kIHRoZSByYXRpbyAoY29ycmVjdGVkIG9lIGRpdmlkZWQgYnkgd3QgZm9yIGVhY2ggbG9jYWxpemF0aW9uKQogICMgYW5kIHByb3BhZ2F0ZSB0aGUgZXJyb3JzCiAgbXV0YXRlKGNvcnJfd3QuY3l0byA9IG1lYW5fd3QuY3l0byAtIG1lYW5fYmcsCiAgICAgICAgIGNvcnJfd3QudG90YWwgPSBtZWFuX3d0LnRvdGFsIC0gbWVhbl9iZywKICAgICAgICAgY29ycl9vZS5jeXRvID0gbWVhbl9vZS5jeXRvIC0gbWVhbl9iZywKICAgICAgICAgY29ycl9vZS50b3RhbCA9IG1lYW5fb2UudG90YWwgLSBtZWFuX2JnLAogICAgICAgICBjb3JyLnNkX3d0LmN5dG8gPSBzcXJ0KHNkX3d0LmN5dG8gXiAyICsgc2RfYmcgXiAyKSwKICAgICAgICAgY29yci5zZF93dC50b3RhbCA9IHNxcnQoc2Rfd3QudG90YWwgXiAyICsgc2RfYmcgXiAyKSwKICAgICAgICAgY29yci5zZF9vZS5jeXRvID0gc3FydChzZF9vZS5jeXRvIF4gMiArIHNkX2JnIF4gMiksCiAgICAgICAgIGNvcnIuc2Rfb2UudG90YWwgPSBzcXJ0KHNkX29lLnRvdGFsIF4gMiArIHNkX2JnIF4gMiksCiAgICAgICAgIGNvcnIuc2Vfd3QuY3l0byA9IHNxcnQoc2Vfd3QuY3l0byBeIDIgKyBzZV9iZyBeIDIpLAogICAgICAgICBjb3JyLnNlX3d0LnRvdGFsID0gc3FydChzZV93dC50b3RhbCBeIDIgKyBzZF9iZyBeIDIpLAogICAgICAgICBjb3JyLnNlX29lLmN5dG8gPSBzcXJ0KHNlX29lLmN5dG8gXiAyICsgc2VfYmcgXiAyKSwKICAgICAgICAgY29yci5zZV9vZS50b3RhbCA9IHNxcnQoc2Vfb2UudG90YWwgXiAyICsgc2RfYmcgXiAyKSwKICAgICAgICAgY29ycl9yYXRpby5jeXRvID0gY29ycl9vZS5jeXRvIC8gY29ycl93dC5jeXRvLAogICAgICAgICBjb3JyLnNkX3JhdGlvLmN5dG8gPSBjb3JyX3JhdGlvLmN5dG8qIHNxcnQoCiAgICAgICAgICAgKGNvcnIuc2Rfb2UuY3l0byAvIGNvcnJfb2UuY3l0bykgXiAyICsgKGNvcnIuc2Rfd3QuY3l0byAvIGNvcnJfd3QuY3l0bykgXiAyKSwKICAgICAgICAgY29yci5zZV9yYXRpby5jeXRvPSBjb3JyX3JhdGlvLmN5dG8qIHNxcnQoCiAgICAgICAgICAgKGNvcnIuc2Vfb2UuY3l0byAvIGNvcnJfb2UuY3l0bykgXiAyICsgKGNvcnIuc2Vfd3QuY3l0byAvIGNvcnJfd3QuY3l0bykgXiAyKSwgCiAgICAgICAgIGNvcnJfcmF0aW8udG90YWwgPSBjb3JyX29lLnRvdGFsIC8gY29ycl93dC50b3RhbCwKICAgICAgICAgY29yci5zZF9yYXRpby50b3RhbCA9IGNvcnJfcmF0aW8udG90YWwgKiBzcXJ0KAogICAgICAgICAgIChjb3JyLnNkX29lLnRvdGFsIC8gY29ycl9vZS50b3RhbCkgXiAyICsgKGNvcnIuc2Rfd3QudG90YWwgLyBjb3JyX3d0LnRvdGFsKSBeIDIpLAogICAgICAgICBjb3JyLnNlX3JhdGlvLnRvdGFsID0gY29ycl9yYXRpby50b3RhbCAqIHNxcnQoCiAgICAgICAgICAgKGNvcnIuc2Vfb2UudG90YWwgLyBjb3JyX29lLnRvdGFsKSBeIDIgKyAoY29yci5zZV93dC50b3RhbCAvIGNvcnJfd3QudG90YWwpIF4gMikgCiAgKQoKIyBnbyBiYWNrIHRvIGxvbmcgZm9ybWF0IGRhdGE6IGl0J3MgbmVhciB1bnJlYWRhYmxlLCBidXQgZWFzaWVyIHRvIHBsb3QKbWVhbl9pbnRlbnNpdGllc19sb25nIDwtIG1lYW5faW50ZW5zaXRpZXMgJT4lCiAgcGl2b3RfbG9uZ2VyKCF0aW1lLCAjcHJvY2VzcyBldmVyeXRoaW5nIGV4Y2VwdCBmb3IgdGltZQogICAgICAgICAgICAgICBuYW1lc190byA9IGMoJy52YWx1ZScsICdzaWduYWwnKSwgIyBuZXcgdmFyaWFibGVzCiAgICAgICAgICAgICAgIG5hbWVzX3NlcCA9ICdfJykgJT4lICMgZ2VuZXJhdGUgdmFsdWVzIGZvciBuZXcgdmFyaWFibGVzCiAgc2VwYXJhdGUoc2lnbmFsLCBjKCdzdHJhaW4nLCAnbG9jYWxpemF0aW9uJyksIHNlcCA9ICdbLl0nKSAlPiUKICBtdXRhdGUobG9jYWxpemF0aW9uID0gcmVjb2RlX2ZhY3Rvcihsb2NhbGl6YXRpb24sCiAgICAgICAgICAgICAgICAgICAgICAgICAgICAgICAndG90YWwnID0gJ0NlbGx1bGFyJywKICAgICAgICAgICAgICAgICAgICAgICAgICAgICAgICdjeXRvJyA9ICdDeXRvc29saWMnLAogICAgICAgICAgICAgICAgICAgICAgICAgICAgICAgKSkKYGBgCgojIyMgUmF3IHBsb3QKClRoaXMgcGxvdCBzaG93cyB0aHJlZSBjbGFzc2VzIG9mIHNpZ25hbCBpbnRlbnNpdHk6IAogIAogICogYmFja2dyb3VuZCwgCiAgKiBFR0ZQLUVkZTEgaW4gb3ZlcmV4cHJlc3NpbmcgY2VsbHMsIGFuZCAKICAqIEVkZTEtRUdGUCBpbiB3aWxkLXR5cGUgY2VsbHMuCgpMaW5lIGlzIG1lYW4sIHNoYWRlZCBhcmVhIGlzIDk1JSBjb25maWRlbmNlIGludGVydmFsLgoKYGBge3J9CnJhd19wbG90IDwtIGdncGxvdChpbnRlbnNpdGllcywKICAgICAgICAgICAgICAgICAgIGFlcyh4ID0gdGltZSwgeSA9IGludGVuc2l0eSkpICsKICBzY2FsZV9jb2xvcl9icmV3ZXIocGFsZXR0ZSA9ICdTZXQyJykrCiAgc2NhbGVfZmlsbF9icmV3ZXIocGFsZXR0ZSA9ICdTZXQyJykrCiAgc3RhdF9zdW1tYXJ5KGFlcyhjb2xvdXIgPSBzaWduYWwpLAogICAgICAgICAgICAgICBmdW4ueSA9IG1lYW4sIGdlb20gPSAnbGluZScsIG5hLnJtID0gVCkrCiAgc3RhdF9zdW1tYXJ5KGFlcyhmaWxsID0gc2lnbmFsKSwKICAgICAgICAgICAgICAgZnVuLmRhdGEgPSAnbWVhbl9jbF9ub3JtYWwnLAogICAgICAgICAgICAgICAjZnVuLmFyZ3MgPSBsaXN0KG11bHQgPSAxKSwgIyBtdWx0ID0gaG93IG1hbnkgZGV2aWF0aW9ucwogICAgICAgICAgICAgICBnZW9tID0gJ3JpYmJvbicsIGFscGhhID0gMC4zLCBuYS5ybSA9IFQpCnByaW50KHJhd19wbG90KQpgYGAgCgojIyMgQmFja2dyb3VuZCBjb3JyZWN0ZWQKClBsb3Qgc2hvd3MgdHdvIGNsYXNzZXMgb2Ygc2lnbmFsIGludGVuc2l0eToKICAKICAqIEVHRlAtRWRlMSBpbiBvdmVyZXhwcmVzc2luZyBjZWxscywgYW5kCiAgKiBFZGUxLUVHRlAgaW4gd2lsZC10eXBlIGNlbGxzLgogIApCb3RoIHdlcmUgY29ycmVjdGVkIGJ5IHN1YnRyYWN0aW5nIHRoZSBiYWNrZ3JvdW5kIHZhbHVlLgoKTGluZSBpcyBtZWFuICsvLSAyeFNFTSAoOTUlIGNvbmZpZGVuY2UgaW50ZXJ2YWwgYmFzZWQgb24gbm9ybWFsIGRpc3RyaWJ1dGlvbik7IAp1bmNlcnRhaW50eSBvZiBiYWNrZ3JvdW5kIG1lYXN1cmVtZW50cyBoYXMgYmVlbiBwcm9wYWdhdGVkIGR1cmluZyBzdWJ0cmFjdGlvbi4KCmBgYHtyfQojYmFzZV9wbG90IDwtIGdncGxvdChtZWFuX2ludGVuc2l0aWVzKQoKY29ycl9wbG90IDwtIG1lYW5faW50ZW5zaXRpZXNfbG9uZyAlPiUKICBmaWx0ZXIoc3RyYWluID09ICd3dCcgfCBzdHJhaW4gPT0gJ29lJyApICU+JQogIG11dGF0ZSh0aW1lID0gdGltZSAvIDYwKSAlPiUKICAgIGdncGxvdChhZXMoeCA9IHRpbWUpKSArCiAgICBnZW9tX2xpbmUoYWVzKHkgPSBjb3JyLAogICAgICAgICAgICAgICAgICBjb2xvdXIgPSBzdHJhaW4pKSsKICAgIGdlb21fcmliYm9uKGFlcyh5bWluID0gY29yciAtIDIgKiBjb3JyLnNlLAogICAgICAgICAgICAgICAgICAgIHltYXggPSBjb3JyICsgMiAqIGNvcnIuc2UsCiAgICAgICAgICAgICAgICAgICAgZmlsbCA9IHN0cmFpbiksCiAgICAgICAgICAgICAgICBhbHBoYSA9IDAuMykrCiAgICBzY2FsZV9jb2xvcl9icmV3ZXIocGFsZXR0ZSA9ICdTZXQyJykrCiAgICBzY2FsZV9maWxsX2JyZXdlcihwYWxldHRlID0gJ1NldDInKSsKICAgIGZhY2V0X2dyaWQocm93cyA9IHZhcnMobG9jYWxpemF0aW9uKSwKICAgICAgICAgICAgICAgc2NhbGVzID0gJ2ZyZWUnLAogICAgICAgICAgICAgICApKwogICAgc2NhbGVfeF9jb250aW51b3VzKGJyZWFrcyA9IHNlcSgwLCA4LCAyKSkrCiAgICBsYWJzKHg9IlRpbWUgKGgpIiwgeSA9ICJNZWFuIGZsdW9yZXNjZW5jZSBpbnRlbnNpdHkgKGEudS4pIikKCnByaW50KGNvcnJfcGxvdCkKbXlfZ2dzYXZlKCdmaWd1cmVzL2NvcnJlY3RlZC5wZGYnKQpgYGAKCgojIyMgTm9ybWFsaXplZAoKUGxvdCBzaG93cyBjeXRvc29saWMgaW50ZW5zaXR5IG9mIEVHRlAtRWRlMSBpbiBvdmVyZXhwcmVzc2luZyBjZWxscyAKbm9ybWFsaXplZCBpbiByZXNwZWN0IHRvIHRoZSBFZGUxLUVHRlAgY3l0b3NvbGljIGludGVuc2l0eSBpbiB3aWxkLXR5cGUgY2VsbHMuIApCb3RoIHdlcmUgY29ycmVjdGVkIHByaW9yIHRvIGRpdmlzaW9uIGJ5IHN1YnRyYWN0aW5nIHRoZSBiYWNrZ3JvdW5kIHZhbHVlLgoKTGluZSBpcyBtZWFuICsvLSAyeFNFTSAoOTUlIGNvbmZpZGVuY2UgaW50ZXJ2YWwgYmFzZWQgb24gbm9ybWFsIGRpc3RyaWJ1dGlvbik7IAplcnJvcnMgZnJvbSBzdWJ0cmFjdGlvbiBhbmQgZGl2aXNpb24gaGF2ZSBiZWVuIHByb3BhZ2F0ZWQuCgpgYGB7cn0KcmF0aW9fcGxvdCA8LSBtZWFuX2ludGVuc2l0aWVzX2xvbmcgJT4lCiAgZmlsdGVyKHN0cmFpbiA9PSAncmF0aW8nKSAlPiUKICAgIGdncGxvdChhZXMoeCA9IHRpbWUpKSArCiAgICBnZW9tX2xpbmUoYWVzKHkgPSBjb3JyLCBjb2xvciA9IGxvY2FsaXphdGlvbikpKwogICAgZ2VvbV9yaWJib24oYWVzKHltaW4gPSBjb3JyIC0gMiAqIGNvcnIuc2UsCiAgICAgICAgICAgICAgICAgICAgeW1heCA9IGNvcnIgKyAyICogY29yci5zZSwKICAgICAgICAgICAgICAgICAgICBmaWxsID0gbG9jYWxpemF0aW9uKSwgYWxwaGEgPSAwLjMpKwogICAgc2NhbGVfY29sb3JfYnJld2VyKHBhbGV0dGUgPSAnU2V0MicpKwogICAgc2NhbGVfZmlsbF9icmV3ZXIocGFsZXR0ZSA9ICdTZXQyJykrCiAgICBsYWJzKHg9IlRpbWUgKG1pbikiLCB5ID0gIk5vcm1hbGl6ZWQgaW50ZW5zaXR5IikKCnByaW50KHJhdGlvX3Bsb3QpCm15X2dnc2F2ZSgnZmlndXJlcy9yYXRpby5wZGYnKQpgYGAKCiMjIFNvdXJjZSBkYXRhCgpgYGB7cn0KIyBzYXZlIGltYWdlCnNhdmUuaW1hZ2UoZmlsZSA9ICdkYXRhL2VkZTFfZ2FsX2luZHVjdGlvbi5SRGF0YScpCmBgYAoKIyMjIC5jc3YgKHJhdyBpbnRlbnNpdGllcykKCmBgYHtyIGVjaG89RkFMU0V9CnhmdW46OmVtYmVkX2ZpbGUoJ2RhdGEvZWRlMV9nYWxfaW5kdWN0aW9uLmNzdicpCmBgYAoKIyMjIC5SRGF0YSAoZXZlcnl0aGluZykKCmBgYHtyIGVjaG89RkFMU0V9CnhmdW46OmVtYmVkX2ZpbGUoJ2RhdGEvZWRlMV9nYWxfaW5kdWN0aW9uLlJEYXRhJykKYGBgCg==
