# Supplementary figures and images for: Condensation of Ede1 promotes the initiation of endocytosis
Source: eLife. 2022 Apr 12;11:e72865. doi: 10.7554/eLife.72865 (PMC9064294; doi:10.7554/eLife.72865)

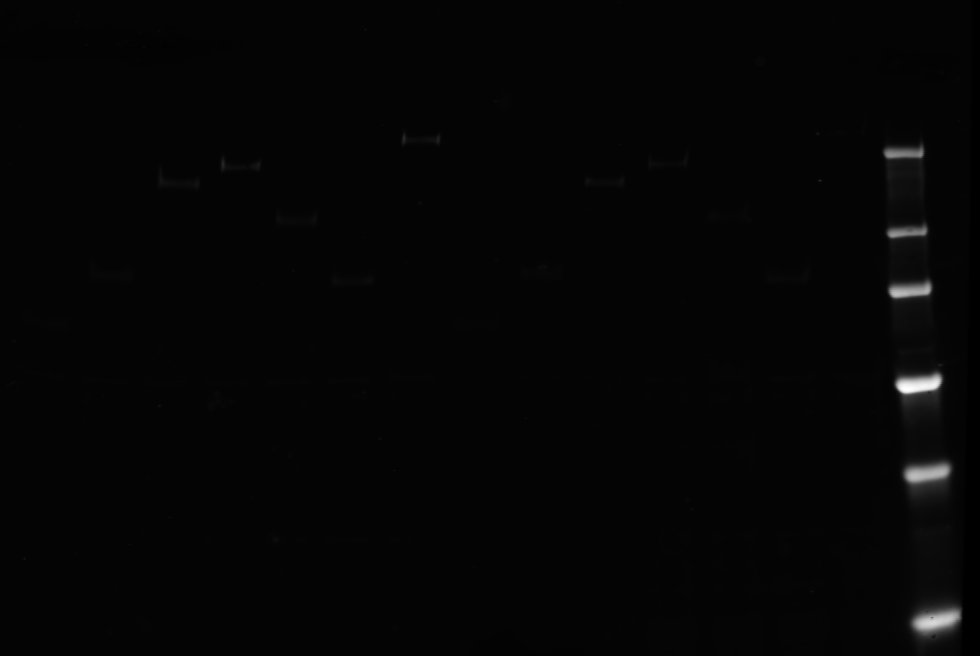

Supplement: Figure 5—figure supplement 1—source data 1. [file elife-72865-fig5-figsupp1-data1.zip › figure5_sup1_data1/rep2_16bit.tif]

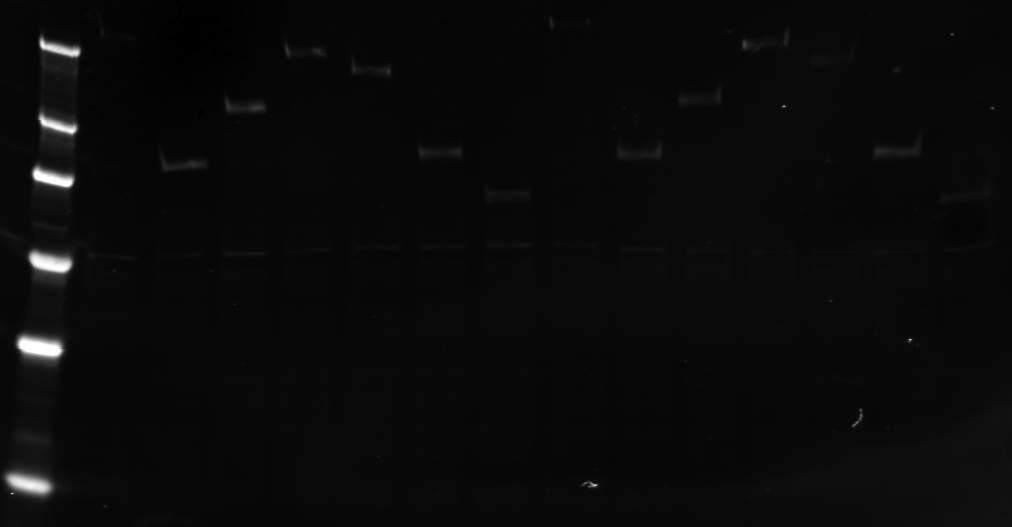

Supplement: Figure 5—figure supplement 1—source data 1. [file elife-72865-fig5-figsupp1-data1.zip › figure5_sup1_data1/rep3_16bit.tif]

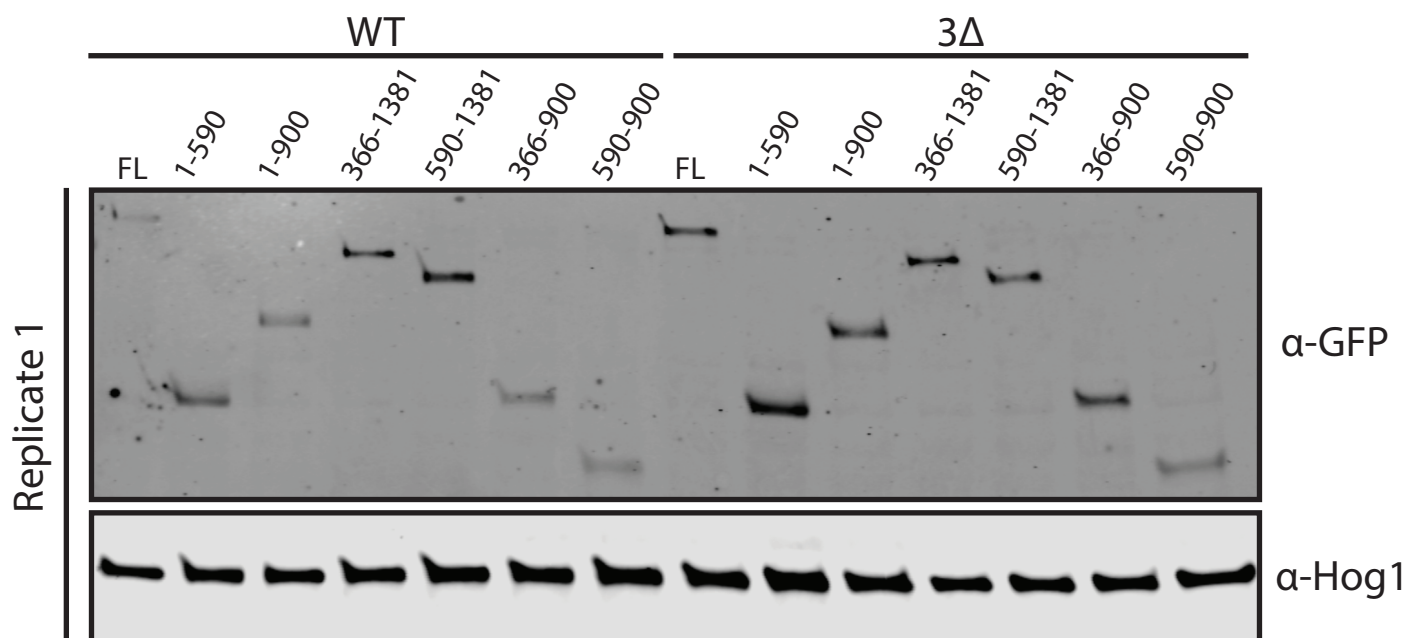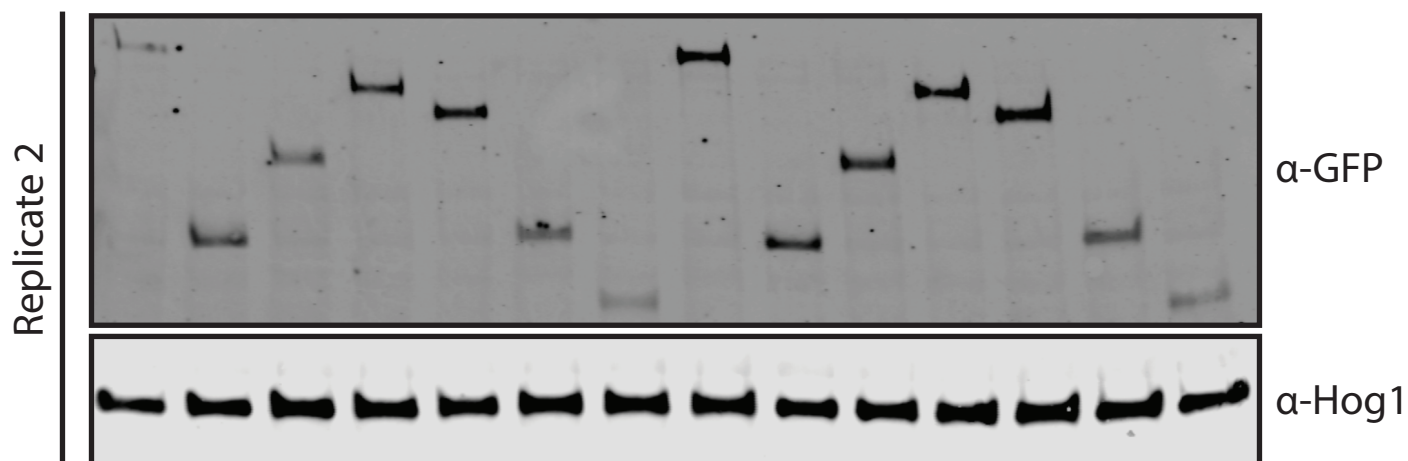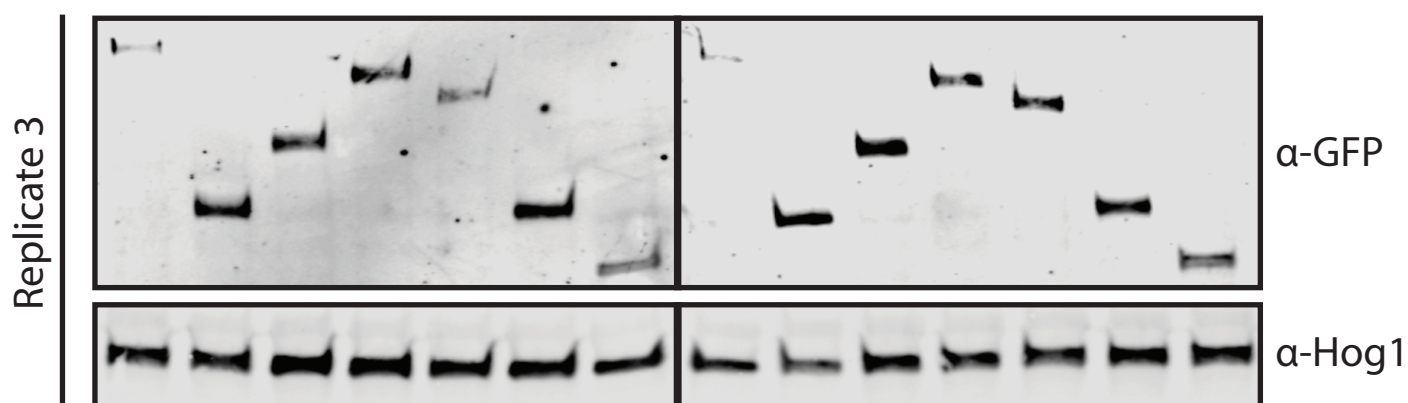

Supplement: Figure 5—figure supplement 1—source data 1. [file elife-72865-fig5-figsupp1-data1.zip › figure5_sup1_data1/ede1_truncation_blots.pdf]

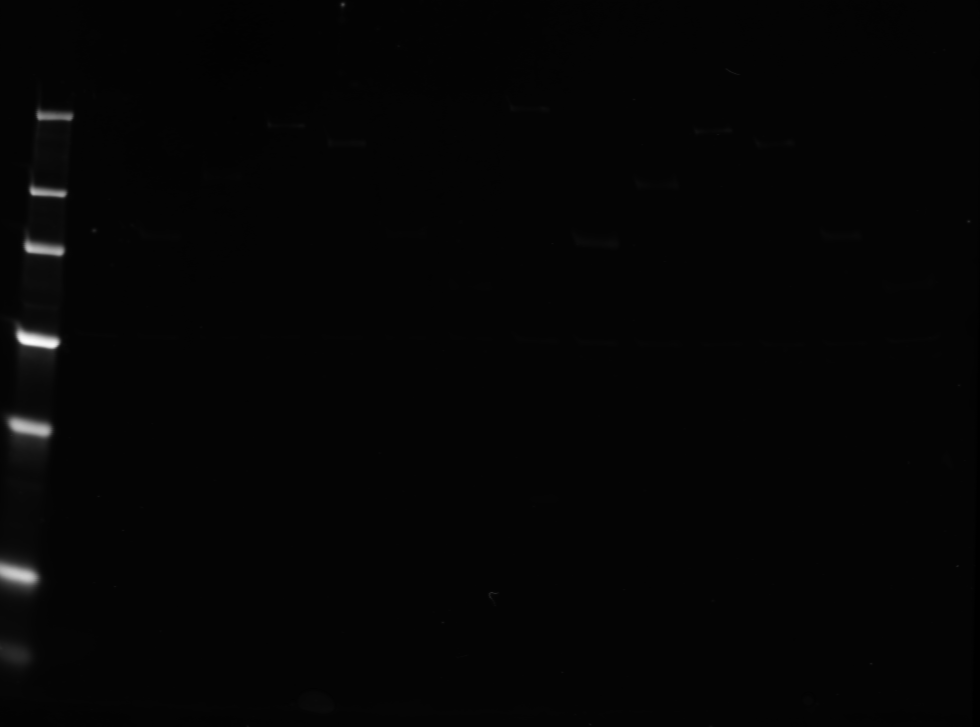

Supplement: Figure 5—figure supplement 1—source data 1. [file elife-72865-fig5-figsupp1-data1.zip › figure5_sup1_data1/rep1_16bit.tif]
