# Supplementary material for: Condensation of Ede1 promotes the initiation of endocytosis
Source: eLife. 2022 Apr 12;11:e72865. doi: 10.7554/eLife.72865 (PMC9064294; doi:10.7554/eLife.72865)
Supplement: Figure 6—source data 1. [file elife-72865-fig6-data1.zip › figure6_data1.html]

Sla1 patch density in Ede1 internal mutants


Code 

- Download Rmd

# Sla1 patch density in Ede1 internal mutants

#### Last compiled on July 30, 2021

# 

## Experiment

### Rationale

The aim of the experiment is to determine patch initiation rates in Ede1 internal domain deletion mutants. We looked at the patch density and lifetimes of late coat protein Sla1 in Ede1 mutants lacking all or some of the central region. This notebook is devoted to the Sla1 patch density.

### Illumination settings

I acquired all data on the Olympus IX81 equipped with a 100x/1.49 objective, using the X-Cite 120PC lamp at 100% intensity and 400 ms exposure for illumination. Light was filtered through a U-MGFPHQ filter cube. I acquired stacks of 26 planes with a step size of 0.2 microns.

### Image processing

Individual non-budding cells were cropped from fields of view. Patch numbers were extracted using Python function `count_patches` from my personal package `mkimage` containing a set of wrappers for `scikit-image` functions. Briefly, the images were median-filtered with a 5 px disk brush, and the filtered images were subtracted from the originals to subtract local background. The background-subtracted images were thresholded using the Yen method. The thresholded images were eroded using the number of non-zero neighbouring pixels in 3D as the erosion criterion. The spots were counted using skimage.measure.label() function with 2-connectivity.

Cross-section area was obtained by median-filtering of the stack with 10px disk brush, calculating the maximum projection image, thresholding using Otsu’s algorithm, and using skimage.measure.regionprops() to measure area. Note that these are pixel counts of *cross-section* area. To determine the total surface area, I assumed that an unbudded cell is spherical (suface area is four times the cross-section area).

This call to `site_counter` was used to process all datasets:

```
process_folder(path, median_radius = 5, erosion_n = 1, con = 2,
                   method = Yen, mask = False, loop = False, save_images = True)
```

`data_cleanup.Rmd` was used to gather all output into tidy data frames with no further modifications.

### List of strains used

| strain | ede1 |
| --- | --- |
| MKY0140 | wt |
| MKY3770 | pq |
| MKY3776 | cc |
| MKY3782 | pqcc |
| MKY0654 | delta |

### Addendum on replicates

Despite my best intentions, there were some discrepancies in the illumination settings between datasets. Datasets #1 and #2 were acquired at 100% lamp power, but #1 had a 50% ND filter inserted in the light path and #2 did not, which I did not know at the time.

As a result, the overall signal in dataset #2 was brighter, but there was more bleaching of the further parts of the stack. This might have resulted in undercounting of some patches at the bottom.

With datasets #3 and #4 I was careful not to repeat the same mistake and acquired everything at 50% lamp power.

Therefore datasets 1, 3 and 4 could be seen as ‘canonical’ independent repeats. However, #2 does not ultimately seem like an outlier, so I included it in the final analysis.

## Per-dataset summary

### Patch number and area

| ede1 | dataset | n | patches\_mean | patches\_sd | patches\_se | area\_mean | area\_sd | area\_se |
| --- | --- | --- | --- | --- | --- | --- | --- | --- |
| wt | 1 | 33 | 30.72727 | 7.702567 | 1.3408449 | 57.01005 | 7.899497 | 1.3751260 |
| wt | 2 | 52 | 25.28846 | 5.496297 | 0.7621992 | 53.95972 | 13.422453 | 1.8613593 |
| wt | 3 | 63 | 29.74603 | 8.090154 | 1.0192636 | 49.81814 | 6.591686 | 0.8304744 |
| wt | 4 | 56 | 29.30357 | 6.717466 | 0.8976592 | 53.99737 | 8.254882 | 1.1031050 |
| pq | 1 | 49 | 27.34694 | 7.512631 | 1.0732330 | 64.07668 | 9.498606 | 1.3569437 |
| pq | 2 | 61 | 23.54098 | 5.687922 | 0.7282638 | 64.85298 | 11.574001 | 1.4818990 |
| pq | 3 | 56 | 24.85714 | 7.590493 | 1.0143223 | 62.52588 | 11.025026 | 1.4732810 |
| pq | 4 | 56 | 25.08929 | 7.012396 | 0.9370709 | 59.07555 | 9.917223 | 1.3252447 |
| cc | 1 | 47 | 22.91489 | 5.356141 | 0.7812735 | 58.75194 | 10.444307 | 1.5234588 |
| cc | 2 | 52 | 20.01923 | 5.859433 | 0.8125572 | 56.04337 | 11.722141 | 1.6255685 |
| cc | 3 | 56 | 20.98214 | 6.397417 | 0.8548908 | 50.73425 | 10.279776 | 1.3736928 |
| cc | 4 | 56 | 21.98214 | 5.937526 | 0.7934354 | 52.72582 | 6.696932 | 0.8949152 |
| pqcc | 1 | 56 | 16.08929 | 6.162280 | 0.8234694 | 57.45513 | 8.006104 | 1.0698607 |
| pqcc | 2 | 62 | 16.80645 | 5.337276 | 0.6778347 | 59.10695 | 11.661839 | 1.4810550 |
| pqcc | 3 | 54 | 16.03704 | 6.933547 | 0.9435362 | 52.34180 | 10.339465 | 1.4070229 |
| pqcc | 4 | 49 | 19.30612 | 5.598378 | 0.7997683 | 54.37294 | 9.698409 | 1.3854870 |
| delta | 1 | 44 | 14.63636 | 6.324890 | 0.9535130 | 58.88947 | 10.932393 | 1.6481203 |
| delta | 2 | 45 | 15.46667 | 5.562047 | 0.8291410 | 56.58791 | 12.111897 | 1.8055350 |
| delta | 3 | 52 | 15.09615 | 6.114142 | 0.8478789 | 50.81073 | 8.869104 | 1.2299234 |
| delta | 4 | 52 | 17.96154 | 6.293356 | 0.8727315 | 57.26936 | 11.048515 | 1.5321534 |

### Sla1 density

We can combine the patch number and area into \(density = \frac{patches}{area}\), calculated individually for each cell. We can summarise the data for each Ede1 mutant in each dataset:

| ede1 | dataset | n | density\_mean | density\_sd | density\_se | density\_median | density\_mad |
| --- | --- | --- | --- | --- | --- | --- | --- |
| wt | 1 | 33 | 0.5382331 | 0.1249763 | 0.0217556 | 0.5509918 | 0.1424483 |
| wt | 2 | 52 | 0.4853156 | 0.1166988 | 0.0161832 | 0.4963251 | 0.1036285 |
| wt | 3 | 63 | 0.5931019 | 0.1156752 | 0.0145737 | 0.5885655 | 0.0961262 |
| wt | 4 | 56 | 0.5488695 | 0.1227789 | 0.0164070 | 0.5656413 | 0.1345622 |
| pq | 1 | 49 | 0.4303778 | 0.1108477 | 0.0158354 | 0.4152123 | 0.0870553 |
| pq | 2 | 61 | 0.3707952 | 0.0950338 | 0.0121678 | 0.3782260 | 0.1032522 |
| pq | 3 | 56 | 0.4020377 | 0.1236939 | 0.0165293 | 0.3800441 | 0.1032181 |
| pq | 4 | 56 | 0.4306633 | 0.1226327 | 0.0163875 | 0.4399799 | 0.0801368 |
| cc | 1 | 47 | 0.3942192 | 0.0851579 | 0.0124216 | 0.4025688 | 0.0699532 |
| cc | 2 | 52 | 0.3679239 | 0.1097261 | 0.0152163 | 0.3763154 | 0.1089664 |
| cc | 3 | 56 | 0.4169928 | 0.1201964 | 0.0160619 | 0.4289882 | 0.1041989 |
| cc | 4 | 56 | 0.4174882 | 0.1025519 | 0.0137041 | 0.4003336 | 0.0996016 |
| pqcc | 1 | 56 | 0.2796635 | 0.0951736 | 0.0127181 | 0.2875115 | 0.0965174 |
| pqcc | 2 | 62 | 0.2906701 | 0.0906537 | 0.0115130 | 0.3039545 | 0.0874739 |
| pqcc | 3 | 54 | 0.3030404 | 0.1107896 | 0.0150766 | 0.2889415 | 0.1137708 |
| pqcc | 4 | 49 | 0.3595033 | 0.0960130 | 0.0137161 | 0.3714628 | 0.1000371 |
| delta | 1 | 44 | 0.2482628 | 0.0983372 | 0.0148249 | 0.2557438 | 0.1065543 |
| delta | 2 | 45 | 0.2789393 | 0.0996907 | 0.0148610 | 0.2756539 | 0.0931660 |
| delta | 3 | 52 | 0.2934675 | 0.1064340 | 0.0147597 | 0.3275947 | 0.0893750 |
| delta | 4 | 52 | 0.3199552 | 0.1152373 | 0.0159805 | 0.3101383 | 0.0986257 |

## Plots

### SuperPlots

I have chosen to show this data using the SuperPlot style. Each point shows density of Sla1-EGFP patches in an individual cell.

Big colour points show mean measurements from four independent repeats.

Range is mean +/- SD, calculated based on the four independent repeat means.

#### Beeswarm

#### Violin

### With significance

Let’s add significance stars based on Tukey’s test.

This is only a subset of comparisons and it’s already cluttered. The alternative is…

#### Letter annotations

In this view, groups sharing at least one letter are not significant at a chosen \(\alpha\) (here, 95%).

Pros:

- less cluttered and simpler to read (5 groups = 10 comparisons)
- focus away from p-values; provide a binary decision on the null hypothesis

Cons:

- cannot distinguish different confidence levels

## Hypothesis testing

### Assumptions

ANOVA and similar parametric tests assume that the errors are normally distributed, with homogeneous variances, and that the samples are independent.

We will test the null hypothesis that mean Sla1 density is the same across different Ede1 strains. We will use repeat-level data for the tests to account for experimental variability.

#### Normality

From the plots it looks like the underlying data is ‘normal enough’, considering that ANOVA can tolerate some departure from normality. We can check the normality of residuals used in the model later, but it might still be interesting to know how normal the underlying data is overall.

If we do a formal test (Shapiro-Wilkes):

| ede1 | n | shapiro.p |
| --- | --- | --- |
| wt | 204 | 0.7403509 |
| pq | 222 | 0.4077729 |
| cc | 211 | 0.1588376 |
| pqcc | 221 | 0.6747696 |
| delta | 193 | 0.0978718 |

Q-Q plots:

The data looks quite normal.

#### Homoscedasticity

4 points per group is probably enough to assess whether the variance is similar in the repeat-level data. Levene’s test:

| df1 | df2 | statistic | p |
| --- | --- | --- | --- |
| 4 | 15 | 0.1598623 | 0.9553962 |

Levene’s cannot reject the null here (variance does not differ between groups).

### One-way ANOVA

Given the null of mean equality, what is the likelihood of obtaining these results?

| term | df | sumsq | meansq | statistic | p.value |
| --- | --- | --- | --- | --- | --- |
| ede1 | 4 | 0.1640381 | 0.0410095 | 37.4362 | 1e-07 |
| Residuals | 15 | 0.0164318 | 0.0010955 | NA | NA |

One-way ANOVA rejects the null with \(p = 10^{-7}\).

#### Diagnostic plots

The residuals look approximately normally distributed (histogram, Q-Q plot) with similar variance (Residuals vs. Fitted, grouped by factor).

### Post-hoc test (Tukey)

Following the rejection of the null by ANOVA, we can use Tukey-Kramer to check pariwse comparisons.

| term | group1 | group2 | null.value | estimate | conf.low | conf.high | p.adj | p.adj.signif |
| --- | --- | --- | --- | --- | --- | --- | --- | --- |
| ede1 | wt | pq | 0 | -0.1329115 | -0.2051799 | -0.0606432 | 3.61e-04 | \*\*\* |
| ede1 | wt | cc | 0 | -0.1422240 | -0.2144923 | -0.0699556 | 1.77e-04 | \*\*\* |
| ede1 | wt | pqcc | 0 | -0.2331607 | -0.3054290 | -0.1608923 | 5.00e-07 | \*\*\*\* |
| ede1 | wt | delta | 0 | -0.2562238 | -0.3284922 | -0.1839555 | 1.00e-07 | \*\*\*\* |
| ede1 | pq | cc | 0 | -0.0093125 | -0.0815808 | 0.0629559 | 9.94e-01 | ns |
| ede1 | pq | pqcc | 0 | -0.1002492 | -0.1725175 | -0.0279808 | 5.02e-03 | \*\* |
| ede1 | pq | delta | 0 | -0.1233123 | -0.1955807 | -0.0510440 | 7.69e-04 | \*\*\* |
| ede1 | cc | pqcc | 0 | -0.0909367 | -0.1632050 | -0.0186683 | 1.09e-02 | \* |
| ede1 | cc | delta | 0 | -0.1139998 | -0.1862682 | -0.0417315 | 1.62e-03 | \*\* |
| ede1 | pqcc | delta | 0 | -0.0230631 | -0.0953315 | 0.0492052 | 8.58e-01 | ns |

Two comparisons do not produce statistically significant differences:

- between ede1∆PQ and ede1∆CC
- between ede1∆PQCC and ede1∆

For all other groups, \(p < 0.05\) (at least); for all comparisons with wild type \(p < 0.001\).

## Overall summary

Summary statistics for all experiments, derived from *mean values* of N independent repeats.

### Final estimates

Final estimates with lower / upper 95% confidence intervals and a comparison to wild type (in %). `half_ci` is just the error for writing CI ranges in the format mean +/- error.

| ede1 | mean | lower | upper | proc\_wt | half\_ci |
| --- | --- | --- | --- | --- | --- |
| wt | 0.541 | 0.471 | 0.612 | 100 | 0.070 |
| pq | 0.408 | 0.363 | 0.454 | 75 | 0.045 |
| cc | 0.399 | 0.362 | 0.437 | 74 | 0.037 |
| pqcc | 0.308 | 0.252 | 0.365 | 57 | 0.056 |
| delta | 0.285 | 0.238 | 0.333 | 53 | 0.048 |

### Conclusions

1. All mutations cause a significant reduction in patch density from wild type
2. Ede1∆PQCC is indistinguishable from full Ede1 deletion, causes ~50% reduction in density
3. Individual PQ / CC deletions have intermediate defects

### More statistics

| ede1 | N | mean | sd | se | median | mad |
| --- | --- | --- | --- | --- | --- | --- |
| wt | 4 | 0.541 | 0.044 | 0.022 | 0.544 | 0.041 |
| pq | 4 | 0.408 | 0.028 | 0.014 | 0.416 | 0.021 |
| cc | 4 | 0.399 | 0.023 | 0.012 | 0.406 | 0.017 |
| pqcc | 4 | 0.308 | 0.035 | 0.018 | 0.297 | 0.017 |
| delta | 4 | 0.285 | 0.030 | 0.015 | 0.286 | 0.030 |

### More statistics (observation-level)

So far we have mostly looked at the statistics derived from experiment-level means. For completeness, the table below reports number of observations and density statistic derived from pooled observations from all repeats, in each group:

| ede1 | n | mean | sd | se | median | mad | 25% | 75% |
| --- | --- | --- | --- | --- | --- | --- | --- | --- |
| wt | 204 | 0.545 | 0.125 | 0.009 | 0.551 | 0.132 | 0.459 | 0.633 |
| pq | 222 | 0.407 | 0.115 | 0.008 | 0.408 | 0.104 | 0.328 | 0.473 |
| cc | 211 | 0.400 | 0.107 | 0.007 | 0.399 | 0.099 | 0.332 | 0.465 |
| pqcc | 221 | 0.306 | 0.102 | 0.007 | 0.308 | 0.100 | 0.240 | 0.375 |
| delta | 193 | 0.287 | 0.108 | 0.008 | 0.289 | 0.107 | 0.218 | 0.361 |

## Source data

### .csv

Download sla1\_density.csv

### .RData

Download sla1\_density.RData

## Session info

```
## R version 4.1.0 (2021-05-18)
## Platform: x86_64-apple-darwin17.0 (64-bit)
## Running under: macOS Mojave 10.14.6
## 
## Matrix products: default
## BLAS:   /Library/Frameworks/R.framework/Versions/4.1/Resources/lib/libRblas.dylib
## LAPACK: /Library/Frameworks/R.framework/Versions/4.1/Resources/lib/libRlapack.dylib
## 
## locale:
## [1] en_US.UTF-8/en_US.UTF-8/en_US.UTF-8/C/en_US.UTF-8/en_US.UTF-8
## 
## attached base packages:
## [1] stats     graphics  grDevices utils     datasets  methods   base     
## 
## other attached packages:
##  [1] multcompView_0.1-8 knitr_1.33         rstatix_0.7.0      broom_0.7.6       
##  [5] ggsignif_0.6.1     ggbeeswarm_0.6.0   forcats_0.5.1      stringr_1.4.0     
##  [9] dplyr_1.0.6        purrr_0.3.4        readr_1.4.0        tidyr_1.1.3       
## [13] tibble_3.1.2       ggplot2_3.3.3      tidyverse_1.3.1   
## 
## loaded via a namespace (and not attached):
##  [1] fs_1.5.0            lubridate_1.7.10    RColorBrewer_1.1-2 
##  [4] httr_1.4.2          tools_4.1.0         backports_1.2.1    
##  [7] utf8_1.2.1          R6_2.5.0            rpart_4.1-15       
## [10] vipor_0.4.5         Hmisc_4.5-0         DBI_1.1.1          
## [13] colorspace_2.0-1    nnet_7.3-16         withr_2.4.2        
## [16] gridExtra_2.3       tidyselect_1.1.1    curl_4.3.1         
## [19] compiler_4.1.0      cli_2.5.0           rvest_1.0.0        
## [22] htmlTable_2.2.1     xml2_1.3.2          labeling_0.4.2     
## [25] checkmate_2.0.0     scales_1.1.1        digest_0.6.27      
## [28] foreign_0.8-81      rmarkdown_2.8       rio_0.5.26         
## [31] base64enc_0.1-3     jpeg_0.1-8.1        pkgconfig_2.0.3    
## [34] htmltools_0.5.1.1   dbplyr_2.1.1        highr_0.9          
## [37] htmlwidgets_1.5.3   rlang_0.4.11        readxl_1.3.1       
## [40] rstudioapi_0.13     farver_2.1.0        generics_0.1.0     
## [43] jsonlite_1.7.2      zip_2.2.0           car_3.0-10         
## [46] magrittr_2.0.1      Formula_1.2-4       Matrix_1.3-3       
## [49] Rcpp_1.0.6          munsell_0.5.0       fansi_0.5.0        
## [52] abind_1.4-5         lifecycle_1.0.0     stringi_1.6.2      
## [55] yaml_2.2.1          carData_3.0-4       grid_4.1.0         
## [58] crayon_1.4.1        lattice_0.20-44     haven_2.4.1        
## [61] splines_4.1.0       hms_1.1.0           pillar_1.6.1       
## [64] reprex_2.0.0        glue_1.4.2          evaluate_0.14      
## [67] latticeExtra_0.6-29 data.table_1.14.0   modelr_0.1.8       
## [70] vctrs_0.3.8         png_0.1-7           cellranger_1.1.0   
## [73] gtable_0.3.0        assertthat_0.2.1    xfun_0.23          
## [76] openxlsx_4.2.3      mime_0.10           survival_3.2-11    
## [79] beeswarm_0.3.1      cluster_2.1.2       ellipsis_0.3.2
```

LS0tCnRpdGxlOiAiU2xhMSBwYXRjaCBkZW5zaXR5IGluIEVkZTEgaW50ZXJuYWwgbXV0YW50cyIKZGF0ZTogIkxhc3QgY29tcGlsZWQgb24gYHIgZm9ybWF0KFN5cy50aW1lKCksICclQiAlZCwgJVknKWAiCm91dHB1dDogCiAgaHRtbF9kb2N1bWVudDoKICAgIGNvZGVfZG93bmxvYWQ6IFRSVUUKZWRpdG9yX29wdGlvbnM6IAogIGNodW5rX291dHB1dF90eXBlOiBpbmxpbmUKLS0tCgpgYGB7ciBzZXR1cCwgaW5jbHVkZT1GQUxTRX0Ka25pdHI6Om9wdHNfY2h1bmskc2V0KGVjaG8gPSBGQUxTRSwgbWVzc2FnZT1GQUxTRSwgd2FybmluZz1GQUxTRSwKICAgICAgICAgICAgICAgICAgICAgIGRwaSA9IDk2LCBmaWcud2lkdGggPSA0LCBmaWcuaGVpZ2h0ID0gMykKYGBgCgpgYGB7ciBsaWJzfQpsaWJyYXJ5KHRpZHl2ZXJzZSkKbGlicmFyeShnZ2JlZXN3YXJtKQpsaWJyYXJ5KGdnc2lnbmlmKQpsaWJyYXJ5KGJyb29tKQpsaWJyYXJ5KHJzdGF0aXgpCmxpYnJhcnkoa25pdHIpCmxpYnJhcnkobXVsdGNvbXBWaWV3KQpgYGAKCmBgYHtyIGxvYWR9CiMgTG9hZCBkYXRhIGdlbmVyYXRlZCBieSBjbGVhbnVwIG5vdGVib29rCnJtKGxpc3QgPSBscygpKQpsb2FkKCdkYXRhL3NsYTFfZGVuc2l0eS5SRGF0YScpCmBgYAoKYGBge3IgdGhlbWV9CiMgQ3VzdG9tIGdncGxvdDIgdGhlbWUKIyAtLS0tLS0tLS0tLS0tLS0tLS0tLQoKIyBtaW5pbWFsIHRoZW1lIHdpdGggYm9yZGVyCiMgYmFzZWQgb24gdGhlbWVfbGluZWRyYXcgd2l0aG91dCB0aGUgZ3JpZCBsaW5lcwojIGFsc28gdHJ5aW5nIHRvIHJlbW92ZSBhbGwgYmFja2dyb3VuZHMgYW5kIG1hcmdpbnMKIyB0aGUgYWltIGlzIHRvIG1ha2UgaXQgYXMgZWFzeSBhcyBwb3NzaWJsZSB0byBlZGl0IGluIGlsbHVzdHJhdG9yCgp0aGVtZV9jbGVhbiA8LSBmdW5jdGlvbihiYXNlX3NpemUgPSAxMSwgYmFzZV9mYW1pbHkgPSAiIiwKICAgICAgICAgICAgICAgICAgICAgICAgYmFzZV9saW5lX3NpemUgPSBiYXNlX3NpemUgLyAyMiwKICAgICAgICAgICAgICAgICAgICAgICAgYmFzZV9yZWN0X3NpemUgPSBiYXNlX3NpemUgLyAyMikgewogIHRoZW1lX2xpbmVkcmF3KAogICAgYmFzZV9zaXplID0gYmFzZV9zaXplLAogICAgYmFzZV9mYW1pbHkgPSBiYXNlX2ZhbWlseSwKICAgIGJhc2VfbGluZV9zaXplID0gYmFzZV9saW5lX3NpemUsCiAgICBiYXNlX3JlY3Rfc2l6ZSA9IGJhc2VfcmVjdF9zaXplCiAgKSAlK3JlcGxhY2UlCiAgICB0aGVtZSgKICAgICAgIyBubyBncmlkIGFuZCBubyBiYWNrZ3JvdW5kcyBpZiBJIGNhbiBoZWxwIGl0CiAgICAgIGxlZ2VuZC5iYWNrZ3JvdW5kID0gIGVsZW1lbnRfYmxhbmsoKSwKICAgICAgcGFuZWwuYmFja2dyb3VuZCA9IGVsZW1lbnRfYmxhbmsoKSwKICAgICAgcGFuZWwuZ3JpZCA9IGVsZW1lbnRfYmxhbmsoKSwKICAgICAgcGxvdC5iYWNrZ3JvdW5kID0gZWxlbWVudF9ibGFuaygpLAogICAgICBwbG90Lm1hcmdpbiA9IG1hcmdpbigwLCAwLCAwLCAwKSwKICAgICAgY29tcGxldGUgPSBUUlVFCiAgICApCn0KCiMgU2V0IGRlZmF1bHQgdGhlbWUKIyAtLS0tLS0tLS0tLS0tLS0tLQp0aGVtZV9zZXQodGhlbWVfY2xlYW4oYmFzZV9zaXplID0gMTIsIGJhc2VfZmFtaWx5ID0gIk15cmlhZCBQcm8iKSkKCiMgQ3JlYXRlIGEgZ2dzYXZlIHdyYXBwZXIKIyAtLS0tLS0tLS0tLS0tLS0tLS0tLS0tLQoKIyBUaGlzIHdheSB3ZSBjYW4gc2V0IGEgZGVmYXVsdCBzaXplIGFuZCBkZXZpY2UgZm9yIGFsbCBwbG90cwpteV9nZ3NhdmUgPC0gZnVuY3Rpb24oZmlsZW5hbWUsIHBsb3QgPSBsYXN0X3Bsb3QoKSwKICAgICAgICAgICAgICAgICAgICAgIGRldmljZSA9IGNhaXJvX3BkZiwgdW5pdHMgPSAibW0iLAogICAgICAgICAgICAgICAgICAgICAgd2lkdGggPSAxMDAsIGhlaWdodCA9IDgwLCAuLi4pewogIGdnc2F2ZShmaWxlbmFtZSA9IGZpbGVuYW1lLCBwbG90ID0gcGxvdCwKICAgICAgICAgZGV2aWNlID0gZGV2aWNlLCB1bml0cyA9IHVuaXRzLAogICAgICAgICBoZWlnaHQgPSBoZWlnaHQsIHdpZHRoID0gd2lkdGgsICAuLi4pCiAgfQpgYGAKCmBgYHtyIGZ1bmN0aW9uc30KIyBjb3VwbGUgb2Ygc21hbGwgZnVuY3Rpb25zIGZvciBleHRyYWN0aW5nIGNvbXBhcmlzb25zCiMgaW50byBhIGZvcm1hdCB1bmRlcnN0YW5kYWJsZSBieSBnZ3Bsb3QKCiMnIEFkZCBsZXR0ZXIgZ3JvdXAgbGFiZWxzIGdlbmVyYXRlZCBieSBUdWtleSdzIEhTRAojJwojJyBUaGlzIGZ1bmN0aW9uIHBlcmZvcm1zIEFOT1ZBIGFuZCBUdWtleSdzIEhTRCwKIycgZXh0cmFjdHMgdGhlIGxldHRlciBsYWJlbHMgYW5kIGF0dGFjaGVzIHRoZW0KIycgdG8gdGhlIG9yaWdpbmFsIGRhdGEuCiMnCiMnIEBwYXJhbSB4OiBkZiBvciB0aWJibGUsIHRoZSBkYXRhIChsb25nIGZvcm1hdCEpCiMnIEBwYXJhbSB5dmFyOiBjaHIsIG5hbWUgb2YgdGhlIGRlcGVuZGVudCB2YXJpYWJsZQojJyBAcGFyYW0geHZhcjogY2hyLCBuYW1lIG9mIHRoZSBpbmRlcGVuZGVudCB2YXJpYWJsZQojJyBAcGFyYW0gYWxwaGE6IGRibCwgY29uZmlkZW5jZSBsZXZlbCBwYXNzZWQgb24gdG8gVHVrZXkncyB0ZXN0CiMnCmFkZF90dWtleV9sYWJlbHMgPC0gZnVuY3Rpb24oeCwgeXZhciwgeHZhciwgYWxwaGEgPSAwLjk1KXsKICBhb3ZfZm9ybSA8LSBmb3JtdWxhKHBhc3RlKHl2YXIsICd+JywgeHZhcikpCiAgYW5vdmEgPC0gYW92KGZvcm11bGEgPSBhb3ZfZm9ybSwgZGF0YSA9IHgpCiAgdHVrZXkgPC0gVHVrZXlIU0QoYW5vdmEsIHdoaWNoID0geHZhciwgY29uZi5sZXZlbCA9IGFscGhhKQogICMgRXh0cmFjdCBsYWJlbHMgYW5kIGZhY3RvciBsZXZlbHMgZnJvbSBUdWtleSBwb3N0LWhvYyAKICB4X2xhYmVscyA8LSBhc190aWJibGUoCiAgICBtdWx0Y29tcExldHRlcnM0KGFub3ZhLCB0dWtleSlbW3h2YXJdXSRMZXR0ZXJzLAogICAgcm93bmFtZXMgPSB4dmFyCiAgICApCiAgCiAgaWYgKGlzLmZhY3Rvcih4W1t4dmFyXV0pKSB7CiAgICB4X2xhYmVsc1tbeHZhcl1dIDwtIGZhY3RvcigKICAgICAgeF9sYWJlbHNbW3h2YXJdXSwgbGV2ZWxzID0gbGV2ZWxzKHhbW3h2YXJdXSkKICAgICkKICB9CiAgCiAgeF9sYWJlbHMgPC0gcmVuYW1lKHhfbGFiZWxzLCB0dWtleV9ncm91cCA9IHZhbHVlKQogIHggPC0gbGVmdF9qb2luKHgsIHhfbGFiZWxzLCBieSA9IHh2YXIpCiAgCiAgcmV0dXJuKHgpCiAgfQoKCiMnIEV4dHJhY3QgY29tcGFyaXNvbnMgZnJvbSByc3RhdGl4IHRpZHkgdGVzdHMKIycgCiMnIFRoaXMgZnVuY3Rpb24gc3Vic2V0cyB0aGUgc2VsZWN0ZWQgY29tcGFyaXNvbnMgaW4gYSBUdWtleSwKIycgRHVubiBvciBzaW1pbGFyIHRlc3QgZG9uZSBieSByc3RhdGl4LgojJyBJdCBjb252ZXJ0cyBncm91cHMgdG8gYSBsaXN0IG9mIHZlY3RvcnMgdGhhdCBjYW4gYmUgcGFzc2VkIHRvIGdlb21fc2lnbmlmCiMnCiMnCiMnIEBwYXJhbSB4OiBkZiBvciB0aWJibGUsIHRoZSBjb21wYXJpc29uIHJlc3VsdHMKIycgQHBhcmFtIHJvd3M6IGludGVnZXIgdmVjdG9yLCB0aGUgcm93cyB3aXRoIGRlc2lyZWQgY29tcGFyaXNvbnMKZXh0cmFjdF9jb21wYXJpc29ucyA8LSBmdW5jdGlvbih4LCByb3dzKXsKICB4X3N1YnNldCA8LSB4W3Jvd3MsXSAlPiUKICAgIC5bbnJvdyguKToxLF0KICAKICB4X2NvbXBhcmlzb25zIDwtIHhfc3Vic2V0ICU+JQogICAgc2VsZWN0KGdyb3VwMSwgZ3JvdXAyKSAlPiUKICAgIHQoKSAlPiUKICAgIGFzLmRhdGEuZnJhbWUoKSAlPiUKICAgIGFzLmxpc3QKICB4X2Fubm90YXRpb25zIDwtIHhfc3Vic2V0JHAuYWRqLnNpZ25pZiAlPiUKICAgIGFzLnZlY3RvcigpCiAgCiAgc2lnbmlmaWNhbmNlIDwtIGxpc3QoCiAgICBjb21wYXJpc29ucyA9IHhfY29tcGFyaXNvbnMsCiAgICBhbm5vdGF0aW9ucyA9IHhfYW5ub3RhdGlvbnMKICApCiAgCiAgcmV0dXJuKHNpZ25pZmljYW5jZSkKfQpgYGAKCiMgey50YWJzZXQgLnRhYnNldC1waWxsc30KCiMjIEV4cGVyaW1lbnQKCiMjIyBSYXRpb25hbGUKClRoZSBhaW0gb2YgdGhlIGV4cGVyaW1lbnQgaXMgdG8gZGV0ZXJtaW5lIHBhdGNoIGluaXRpYXRpb24gcmF0ZXMgCmluIEVkZTEgaW50ZXJuYWwgZG9tYWluIGRlbGV0aW9uIG11dGFudHMuCldlIGxvb2tlZCBhdCB0aGUgcGF0Y2ggZGVuc2l0eSBhbmQgbGlmZXRpbWVzIG9mIGxhdGUgY29hdCBwcm90ZWluIFNsYTEgCmluIEVkZTEgbXV0YW50cyBsYWNraW5nIGFsbCBvciBzb21lIG9mIHRoZSBjZW50cmFsIHJlZ2lvbi4KVGhpcyBub3RlYm9vayBpcyBkZXZvdGVkIHRvIHRoZSBTbGExIHBhdGNoIGRlbnNpdHkuCgojIyMgSWxsdW1pbmF0aW9uIHNldHRpbmdzCgpJIGFjcXVpcmVkIGFsbCBkYXRhIG9uIHRoZSBPbHltcHVzIElYODEKZXF1aXBwZWQgd2l0aCBhIDEwMHgvMS40OSBvYmplY3RpdmUsCnVzaW5nIHRoZSBYLUNpdGUgMTIwUEMgbGFtcCBhdCAxMDAlIGludGVuc2l0eQphbmQgNDAwIG1zIGV4cG9zdXJlIGZvciBpbGx1bWluYXRpb24uCkxpZ2h0IHdhcyBmaWx0ZXJlZCB0aHJvdWdoIGEgVS1NR0ZQSFEgZmlsdGVyIGN1YmUuCkkgYWNxdWlyZWQgc3RhY2tzIG9mIDI2IHBsYW5lcyAKd2l0aCBhIHN0ZXAgc2l6ZSBvZiAwLjIgbWljcm9ucy4KCiMjIyBJbWFnZSBwcm9jZXNzaW5nCgpJbmRpdmlkdWFsIG5vbi1idWRkaW5nIGNlbGxzIHdlcmUgY3JvcHBlZCBmcm9tIGZpZWxkcyBvZiB2aWV3LgpQYXRjaCBudW1iZXJzIHdlcmUgZXh0cmFjdGVkIHVzaW5nIFB5dGhvbiBmdW5jdGlvbiBgY291bnRfcGF0Y2hlc2AKZnJvbSBteSBwZXJzb25hbCBwYWNrYWdlIGBta2ltYWdlYApjb250YWluaW5nIGEgc2V0IG9mIHdyYXBwZXJzIGZvciBgc2Npa2l0LWltYWdlYCBmdW5jdGlvbnMuCkJyaWVmbHksIHRoZSBpbWFnZXMgd2VyZSBtZWRpYW4tZmlsdGVyZWQgd2l0aCBhIDUgcHggZGlzayBicnVzaCwKYW5kIHRoZSBmaWx0ZXJlZCBpbWFnZXMgd2VyZSBzdWJ0cmFjdGVkIGZyb20gdGhlIG9yaWdpbmFscwp0byBzdWJ0cmFjdCBsb2NhbCBiYWNrZ3JvdW5kLgpUaGUgYmFja2dyb3VuZC1zdWJ0cmFjdGVkIGltYWdlcyB3ZXJlIHRocmVzaG9sZGVkIHVzaW5nIHRoZSBZZW4gbWV0aG9kLgpUaGUgdGhyZXNob2xkZWQgaW1hZ2VzIHdlcmUgZXJvZGVkIHVzaW5nIHRoZSBudW1iZXIgb2Ygbm9uLXplcm8KbmVpZ2hib3VyaW5nIHBpeGVscyBpbiAzRCBhcyB0aGUgZXJvc2lvbiBjcml0ZXJpb24uClRoZSBzcG90cyB3ZXJlIGNvdW50ZWQgdXNpbmcgc2tpbWFnZS5tZWFzdXJlLmxhYmVsKCkgZnVuY3Rpb24gd2l0aCAyLWNvbm5lY3Rpdml0eS4KCkNyb3NzLXNlY3Rpb24gYXJlYSB3YXMgb2J0YWluZWQgYnkgCm1lZGlhbi1maWx0ZXJpbmcgb2YgdGhlIHN0YWNrIHdpdGggMTBweCBkaXNrIGJydXNoLCAKY2FsY3VsYXRpbmcgdGhlIG1heGltdW0gcHJvamVjdGlvbiBpbWFnZSwgCnRocmVzaG9sZGluZyB1c2luZyBPdHN1J3MgYWxnb3JpdGhtLAphbmQgdXNpbmcgc2tpbWFnZS5tZWFzdXJlLnJlZ2lvbnByb3BzKCkgdG8gbWVhc3VyZSBhcmVhLgpOb3RlIHRoYXQgdGhlc2UgYXJlIHBpeGVsIGNvdW50cyBvZiAqY3Jvc3Mtc2VjdGlvbiogYXJlYS4gClRvIGRldGVybWluZSB0aGUgdG90YWwgc3VyZmFjZSBhcmVhLCAKSSBhc3N1bWVkIHRoYXQgYW4gdW5idWRkZWQgY2VsbCBpcyBzcGhlcmljYWwKKHN1ZmFjZSBhcmVhIGlzIGZvdXIgdGltZXMgdGhlIGNyb3NzLXNlY3Rpb24gYXJlYSkuCgpUaGlzIGNhbGwgdG8gYHNpdGVfY291bnRlcmAgd2FzIHVzZWQgdG8gcHJvY2VzcyBhbGwgZGF0YXNldHM6CgpgYGAKcHJvY2Vzc19mb2xkZXIocGF0aCwgbWVkaWFuX3JhZGl1cyA9IDUsIGVyb3Npb25fbiA9IDEsIGNvbiA9IDIsCiAgICAgICAgICAgICAgICAgICBtZXRob2QgPSBZZW4sIG1hc2sgPSBGYWxzZSwgbG9vcCA9IEZhbHNlLCBzYXZlX2ltYWdlcyA9IFRydWUpCmBgYAoKYGRhdGFfY2xlYW51cC5SbWRgIHdhcyB1c2VkIHRvIGdhdGhlciBhbGwgb3V0cHV0IGludG8gdGlkeSBkYXRhIGZyYW1lcwp3aXRoIG5vIGZ1cnRoZXIgbW9kaWZpY2F0aW9ucy4KCiMjIyBMaXN0IG9mIHN0cmFpbnMgdXNlZAoKYGBge3Igc3RyYWluc30Ka2FibGUoc3RyYWlucykKYGBgCgojIyMgQWRkZW5kdW0gb24gcmVwbGljYXRlcwoKRGVzcGl0ZSBteSBiZXN0IGludGVudGlvbnMsIHRoZXJlIHdlcmUgc29tZSBkaXNjcmVwYW5jaWVzCmluIHRoZSBpbGx1bWluYXRpb24gc2V0dGluZ3MgYmV0d2VlbiBkYXRhc2V0cy4KRGF0YXNldHMgIzEgYW5kICMyIHdlcmUgYWNxdWlyZWQgYXQgMTAwJSBsYW1wIHBvd2VyLApidXQgIzEgaGFkIGEgNTAlIE5EIGZpbHRlciBpbnNlcnRlZCBpbiB0aGUgbGlnaHQgcGF0aCBhbmQgIzIgZGlkIG5vdCwKd2hpY2ggSSBkaWQgbm90IGtub3cgYXQgdGhlIHRpbWUuCgpBcyBhIHJlc3VsdCwgdGhlIG92ZXJhbGwgc2lnbmFsIGluIGRhdGFzZXQgIzIgd2FzIGJyaWdodGVyLApidXQgdGhlcmUgd2FzIG1vcmUgYmxlYWNoaW5nIG9mIHRoZSBmdXJ0aGVyIHBhcnRzIG9mIHRoZSBzdGFjay4KVGhpcyBtaWdodCBoYXZlIHJlc3VsdGVkIGluIHVuZGVyY291bnRpbmcgb2Ygc29tZSBwYXRjaGVzIGF0IHRoZSBib3R0b20uCgpXaXRoIGRhdGFzZXRzICMzIGFuZCAjNCBJIHdhcyBjYXJlZnVsIG5vdCB0byByZXBlYXQgdGhlIHNhbWUgbWlzdGFrZQphbmQgYWNxdWlyZWQgZXZlcnl0aGluZyBhdCA1MCUgbGFtcCBwb3dlci4KClRoZXJlZm9yZSBkYXRhc2V0cyAxLCAzIGFuZCA0IGNvdWxkIGJlIHNlZW4gYXMgJ2Nhbm9uaWNhbCcgaW5kZXBlbmRlbnQgcmVwZWF0cy4KSG93ZXZlciwgIzIgZG9lcyBub3QgdWx0aW1hdGVseSBzZWVtIGxpa2UgYW4gb3V0bGllciwKc28gSSBpbmNsdWRlZCBpdCBpbiB0aGUgZmluYWwgYW5hbHlzaXMuCgojIyBQZXItZGF0YXNldCBzdW1tYXJ5IHsudGFic2V0fQoKIyMjIFBhdGNoIG51bWJlciBhbmQgYXJlYQoKYGBge3IgbnVtc19kYXRhc2V0fQojIG1lYW5zIGFuZCBTRHMgb2YgbnVtYmVyIG9mIHBhdGNoZXMgYW5kIGFyZWEKc2xhMV9kZW5zaXR5ICU+JQogIGdyb3VwX2J5KGVkZTEsIGRhdGFzZXQpICU+JQogIHN1bW1hcmlzZShuID0gbigpLAogICAgICAgICAgICBhY3Jvc3MoYyhwYXRjaGVzLCBhcmVhKSwgbGlzdChtZWFuID0gbWVhbiwgc2QgPSBzZCwKICAgICAgICAgICAgICAgICAgICAgICAgICAgICAgICAgICAgICAgICAgc2UgPSB+c2QoLngpIC8gc3FydChuKCkpKSkpICU+JQogIGthYmxlKCkKYGBgCgojIyMgU2xhMSBkZW5zaXR5CgpXZSBjYW4gY29tYmluZSB0aGUgcGF0Y2ggbnVtYmVyIGFuZCBhcmVhIGludG8gJGRlbnNpdHkgPSBcZnJhY3twYXRjaGVzfXthcmVhfSQsCmNhbGN1bGF0ZWQgaW5kaXZpZHVhbGx5IGZvciBlYWNoIGNlbGwuCldlIGNhbiBzdW1tYXJpc2UgdGhlIGRhdGEgZm9yIGVhY2ggRWRlMSBtdXRhbnQgaW4gZWFjaCBkYXRhc2V0OgoKYGBge3IgZGVuc2l0eX0Kc2xhMV9kZW5zaXR5X3N0YXRzIDwtIHNsYTFfZGVuc2l0eSAlPiUKICBncm91cF9ieShlZGUxLCBkYXRhc2V0KSAlPiUKICBzdW1tYXJpc2UobiA9IG4oKSwKICAgICAgICAgICAgYWNyb3NzKGRlbnNpdHksCiAgICAgICAgICAgICAgICAgICBsaXN0KG1lYW4gPSBtZWFuLCBzZCA9IHNkLCAKICAgICAgICAgICAgICAgICAgICAgICAgc2UgPSB+IHNkKC54KSAvIHNxcnQobiksCiAgICAgICAgICAgICAgICAgICAgICAgIG1lZGlhbiA9IG1lZGlhbiwgbWFkID0gbWFkKSksCiAgICAgICAgICAgIC5ncm91cHMgPSAnZHJvcCcpCgpzbGExX2RlbnNpdHlfc3RhdHMgJT4lIGthYmxlKCkKYGBgCgojIyBQbG90cyB7LnRhYnNldH0KCmBgYHtyIGRlbnNpdHkuc2NhdHRlcn0KcGxvdF9ibGFuayA8LSBnZ3Bsb3Qoc2xhMV9kZW5zaXR5X3N0YXRzLAogICAgICAgICAgICAgICAgICAgICBhZXMoeCA9IGVkZTEsIHkgPSBkZW5zaXR5X21lYW4pKSArCiAgICAgICAgICAgICAgICAgICAgICAgICAjc2hhcGUgPSBkYXRhc2V0LCBmaWxsID0gZGF0YXNldCkpKwogIGxhYnModGl0bGUgPSBOVUxMLCB4ID0gJ0VkZTEnLCB5ID0gZXhwcmVzc2lvbiggIlNsYTEgcGF0Y2hlcy/CtW0iXjIpKSArCiAgc2NhbGVfeV9jb250aW51b3VzKGJyZWFrcyA9IHNlcSgwLjAsIDEuMCwgMC4xKSkgKwogIHNjYWxlX3NoYXBlX21hbnVhbCh2YWx1ZXMgPSBjKDIxOjI1KSkgKwogIHNjYWxlX2NvbG9yX2JyZXdlcihwYWxldHRlID0gJ1NldDInKSArCiAgc2NhbGVfZmlsbF9icmV3ZXIocGFsZXR0ZSA9ICdTZXQyJykKCnBsb3Rfc2NhdHRlciA8LSBwbG90X2JsYW5rICsKICBnZW9tX3F1YXNpcmFuZG9tKGluaGVyaXQuYWVzID0gRiwgZGF0YSA9IHNsYTFfZGVuc2l0eSwKICAgICAgICAgICAgICAgICAgIGFlcyh4ID0gZWRlMSwgeSA9IGRlbnNpdHksCiAgICAgICAgICAgICAgICAgICAgICAgc2hhcGUgPSBkYXRhc2V0LCMgY29sb3VyID0gZGF0YXNldAogICAgICAgICAgICAgICAgICAgICAgICksCiAgICAgICAgICAgICAgICAgICBjb2xvdXIgPSAnZ3JleTc1JywjIHNoYXBlID0gMSwKICAgICAgICAgICAgICAgICAgIHNob3cubGVnZW5kID0gRiwgc2l6ZSA9IDAuOAogICAgICAgICAgICAgICAgICAgKQoKcGxvdF92aW9saW4gPC0gcGxvdF9ibGFuayArIAogIGdlb21fdmlvbGluKGluaGVyaXQuYWVzID0gRiwKICAgICAgICAgICAgICBkYXRhID0gc2xhMV9kZW5zaXR5LCBhZXMoeCA9IGVkZTEsIHkgPSBkZW5zaXR5KSwKICAgICAgICAgICAgICBjb2xvdXIgPSAnZ3JleTc1JywgZmlsbCA9ICd0cmFuc3BhcmVudCcKICAgICAgICAgICAgICApCmBgYAoKIyMjIFN1cGVyUGxvdHMKCkkgaGF2ZSBjaG9zZW4gdG8gc2hvdyB0aGlzIGRhdGEgdXNpbmcgdGhlIApbU3VwZXJQbG90XShodHRwczovL2RvaS5vcmcvMTAuMTA4My9qY2IuMjAyMDAxMDY0KSBzdHlsZS4KRWFjaCBwb2ludCBzaG93cyBkZW5zaXR5IG9mIFNsYTEtRUdGUCBwYXRjaGVzIGluIGFuIGluZGl2aWR1YWwgY2VsbC4KCkJpZyBjb2xvdXIgcG9pbnRzIHNob3cgbWVhbiBtZWFzdXJlbWVudHMgZnJvbSBmb3VyIGluZGVwZW5kZW50IHJlcGVhdHMuCgpSYW5nZSBpcyBtZWFuICsvLSBTRCwgY2FsY3VsYXRlZCBiYXNlZCBvbiB0aGUgZm91ciBpbmRlcGVuZGVudCByZXBlYXQgbWVhbnMuCgojIyMjIEJlZXN3YXJtCgpgYGB7cn0KcGxvdF9zdXBlciA8LSBwbG90X3NjYXR0ZXIgKwogIGdlb21fcXVhc2lyYW5kb20oYWVzKHNoYXBlID0gZGF0YXNldCwgZmlsbCA9IGRhdGFzZXQpLAogICAgICAgICAgICAgICAgICAgc2hvdy5sZWdlbmQgPSBGLAogICAgICAgICAgICAgICAgICAgd2lkdGggPSAwLjMsIHNpemUgPSAyKSsKICBzdGF0X3N1bW1hcnkoZnVuID0gbWVhbiwgZ2VvbSA9ICdjcm9zc2JhcicsCiAgICAgICAgICAgICAgIHdpZHRoID0gMC41LCBmYXR0ZW4gPSAxKSsKICBzdGF0X3N1bW1hcnkoZnVuLmRhdGEgPSAnbWVhbl9zZGwnLAogICAgICAgICAgICAgICBmdW4uYXJncyA9IGxpc3QobXVsdCA9IDEpLCAKICAgICAgICAgICAgICAgZ2VvbSA9ICdlcnJvcmJhcicsIHdpZHRoID0gMC4yKQogIApwcmludChwbG90X3N1cGVyKQpteV9nZ3NhdmUoJ2ZpZ3VyZXMvZGVuc2l0eV9zdXBlci5wZGYnKQoKYGBgCgojIyMjIFZpb2xpbgoKYGBge3J9CnBsb3Rfc3VwZXJfdmlvbGluIDwtIHBsb3RfdmlvbGluICsKICBnZW9tX3F1YXNpcmFuZG9tKGFlcyhzaGFwZSA9IGRhdGFzZXQsIGZpbGwgPSBkYXRhc2V0KSwKICAgICAgICAgICAgICAgICAgIHNob3cubGVnZW5kID0gRiwKICAgICAgICAgICAgICAgICAgIHdpZHRoID0gMC4zLCBzaXplID0gMikrCiAgc3RhdF9zdW1tYXJ5KGZ1biA9IG1lYW4sIGdlb20gPSAnY3Jvc3NiYXInLAogICAgICAgICAgICAgICB3aWR0aCA9IDAuNSwgZmF0dGVuID0gMSkrCiAgc3RhdF9zdW1tYXJ5KGZ1bi5kYXRhID0gJ21lYW5fc2RsJywKICAgICAgICAgICAgICAgZnVuLmFyZ3MgPSBsaXN0KG11bHQgPSAxKSwKICAgICAgICAgICAgICAgZ2VvbSA9ICdlcnJvcmJhcicsIHdpZHRoID0gMC4yKQoKcHJpbnQocGxvdF9zdXBlcl92aW9saW4pCm15X2dnc2F2ZSgnZmlndXJlcy9kZW5zaXR5X3N1cGVyX3Zpb2xpbi5wZGYnKQpgYGAKCiMjIyBXaXRoIHNpZ25pZmljYW5jZQoKTGV0J3MgYWRkIHNpZ25pZmljYW5jZSBzdGFycyBiYXNlZCBvbiBUdWtleSdzIHRlc3QuCgpgYGB7ciB0ZXN0c30KdHVrZXkgPC0gdHVrZXlfaHNkKHNsYTFfZGVuc2l0eV9zdGF0cywgZGVuc2l0eV9tZWFuIH4gZWRlMSwKICAgICAgICAgICAgICAgICAgIG9yZGVyZWQgPSBUUlVFKQpgYGAKCmBgYHtyfQpzaWduaWZpY2FuY2UgPC0gZXh0cmFjdF9jb21wYXJpc29ucyh0dWtleSwgYygxOjQsIDEwKSkKCnBsb3Rfc3VwZXJfc2lnbmlmIDwtIHBsb3Rfc3VwZXIgKwogIGdlb21fc2lnbmlmKGNvbXBhcmlzb25zID0gc2lnbmlmaWNhbmNlJGNvbXBhcmlzb25zLAogICAgICAgICAgICAgIGFubm90YXRpb25zID0gc2lnbmlmaWNhbmNlJGFubm90YXRpb25zLAogICAgICAgICAgICAgIHN0ZXBfaW5jcmVhc2UgPSAwLjAzLAogICAgICAgICAgICAgIHRpcF9sZW5ndGggPSAwLjAxLCB2anVzdCA9IDAuOCwKICAgICAgICAgICAgICBtYXJnaW5fdG9wID0gLTAuMSkKcHJpbnQocGxvdF9zdXBlcl9zaWduaWYpCm15X2dnc2F2ZSgnZmlndXJlcy9kZW5zaXR5X3N1cGVyX3NpZ25pZi5wZGYnKQpgYGAKClRoaXMgaXMgb25seSBhIHN1YnNldCBvZiBjb21wYXJpc29ucyBhbmQgaXQncyBhbHJlYWR5IGNsdXR0ZXJlZC4KVGhlIGFsdGVybmF0aXZlIGlzLi4uCgojIyMjIExldHRlciBhbm5vdGF0aW9ucwoKSW4gdGhpcyB2aWV3LCBncm91cHMgc2hhcmluZyBhdCBsZWFzdCBvbmUgbGV0dGVyIGFyZSBub3Qgc2lnbmlmaWNhbnQKYXQgYSBjaG9zZW4gJFxhbHBoYSQgKGhlcmUsIDk1JSkuCgpQcm9zOgoKICAqIGxlc3MgY2x1dHRlcmVkIGFuZCBzaW1wbGVyIHRvIHJlYWQgKDUgZ3JvdXBzID0gMTAgY29tcGFyaXNvbnMpCiAgKiBmb2N1cyBhd2F5IGZyb20gcC12YWx1ZXM7IHByb3ZpZGUgYSBiaW5hcnkgZGVjaXNpb24gb24gdGhlIG51bGwgaHlwb3RoZXNpcwogIApDb25zOgoKICAqIGNhbm5vdCBkaXN0aW5ndWlzaCBkaWZmZXJlbnQgY29uZmlkZW5jZSBsZXZlbHMKCmBgYHtyfQpzbGExX2RlbnNpdHlfc3RhdHMgPC0gc2xhMV9kZW5zaXR5X3N0YXRzICU+JQogIGFkZF90dWtleV9sYWJlbHMoJ2RlbnNpdHlfbWVhbicsICdlZGUxJykKYGBgCgpgYGB7cn0KcGxvdF9zdXBlcl9sZXR0ZXJzIDwtIHBsb3Rfc3VwZXIgKwogIGdlb21fdGV4dChkYXRhID0gc2xhMV9kZW5zaXR5X3N0YXRzLAogICAgICAgICAgICBhZXMobGFiZWwgPSB0dWtleV9ncm91cCksIHkgPSBJbmYsIHZqdXN0ID0gMS4yKQogICNzdGF0X3N1bW1hcnkoYWVzKGxhYmVsID0gdHVrZXlfZ3JvdXApLAogICMgICAgICAgICAgICAgZnVuLnkgPSBJbmYsCiAgIyAgICAgICAgICAgICBnZW9tID0gJ3RleHQnLCBuYS5ybSA9IFQsIHZqdXN0ID0gLTAuNSwKICAjICAgICAgICAgICAgICNmdW4uYXJncyA9IGxpc3QoZXJyID0gZXJyb3JzLCBtdWx0ID0gZXJyb3JfcmFuZ2UpCiAgIyAgICAgICAgICAgICApCnByaW50KHBsb3Rfc3VwZXJfbGV0dGVycykKbXlfZ2dzYXZlKCdmaWd1cmVzL2RlbnNpdHlfc3VwZXJfbGV0dGVycy5wZGYnKQpgYGAKCiMjIEh5cG90aGVzaXMgdGVzdGluZyB7LnRhYnNldH0KCiMjIyBBc3N1bXB0aW9ucwoKQU5PVkEgYW5kIHNpbWlsYXIgcGFyYW1ldHJpYyB0ZXN0cyAKYXNzdW1lIHRoYXQgdGhlIGVycm9ycyBhcmUgbm9ybWFsbHkgZGlzdHJpYnV0ZWQsCndpdGggaG9tb2dlbmVvdXMgdmFyaWFuY2VzLAphbmQgdGhhdCB0aGUgc2FtcGxlcyBhcmUgaW5kZXBlbmRlbnQuCgpXZSB3aWxsIHRlc3QgdGhlIG51bGwgaHlwb3RoZXNpcyB0aGF0IG1lYW4gU2xhMSBkZW5zaXR5IGlzIHRoZSBzYW1lCmFjcm9zcyBkaWZmZXJlbnQgRWRlMSBzdHJhaW5zLgpXZSB3aWxsIHVzZSByZXBlYXQtbGV2ZWwgZGF0YSBmb3IgdGhlIHRlc3RzCnRvIGFjY291bnQgZm9yIGV4cGVyaW1lbnRhbCB2YXJpYWJpbGl0eS4KCiMjIyMgTm9ybWFsaXR5CgpGcm9tIHRoZSBwbG90cyBpdCBsb29rcyBsaWtlIHRoZSB1bmRlcmx5aW5nIGRhdGEgaXMgJ25vcm1hbCBlbm91Z2gnLApjb25zaWRlcmluZyB0aGF0IEFOT1ZBIGNhbiB0b2xlcmF0ZSBzb21lIGRlcGFydHVyZSBmcm9tIG5vcm1hbGl0eS4KV2UgY2FuIGNoZWNrIHRoZSBub3JtYWxpdHkgb2YgcmVzaWR1YWxzIHVzZWQgaW4gdGhlIG1vZGVsIGxhdGVyLCBidXQgaXQgbWlnaHQKc3RpbGwgYmUgaW50ZXJlc3RpbmcgdG8ga25vdyBob3cgbm9ybWFsIHRoZSB1bmRlcmx5aW5nIGRhdGEgaXMgb3ZlcmFsbC4KCklmIHdlIGRvIGEgZm9ybWFsIHRlc3QgKFNoYXBpcm8tV2lsa2VzKToKCmBgYHtyfQpzbGExX2RlbnNpdHkgJT4lCiAgZ3JvdXBfYnkoZWRlMSkgJT4lCiAgc3VtbWFyaXNlKG4gPSBuKCksCiAgICAgICAgICAgIHNoYXBpcm8ucCA9IHRpZHkoc2hhcGlyby50ZXN0KGRlbnNpdHkpKSRwLnZhbHVlLCAKICAgICAgICAgICAgLmdyb3VwcyA9ICdkcm9wJykgJT4lCiAga2FibGUoKQpgYGAKClEtUSBwbG90czoKCmBgYHtyIGZpZy5oZWlnaHQ9NiwgZmlnLndpZHRoPTh9CnNsYTFfZGVuc2l0eSAlPiUKICBnZ3Bsb3QoYWVzKHNhbXBsZSA9IGRlbnNpdHkpKSsKICBmYWNldF93cmFwKCdlZGUxJywgc2NhbGVzID0gJ2ZyZWUnKSsgICAgICAgICAgCiAgbGFicyhjb2xvdXIgPSAnRGF0YXNldCcsIHNoYXBlID0gJ0RhdGFzZXQnKSsKICBzdGF0X3FxKHNoYXBlID0gMSkrCiAgc3RhdF9xcV9saW5lKCkKYGBgCgpUaGUgZGF0YSBsb29rcyBxdWl0ZSBub3JtYWwuCgojIyMjIEhvbW9zY2VkYXN0aWNpdHkKCjQgcG9pbnRzIHBlciBncm91cCBpcyBwcm9iYWJseSBlbm91Z2ggdG8gYXNzZXNzCndoZXRoZXIgdGhlIHZhcmlhbmNlIGlzIHNpbWlsYXIgaW4gdGhlIHJlcGVhdC1sZXZlbCBkYXRhLgpMZXZlbmUncyB0ZXN0OgoKYGBge3J9CnNsYTFfZGVuc2l0eV9zdGF0cyAlPiUKICBsZXZlbmVfdGVzdChkZW5zaXR5X21lYW4gfiBlZGUxKSAlPiUKICBrYWJsZSgpCmBgYAoKTGV2ZW5lJ3MgY2Fubm90IHJlamVjdCB0aGUgbnVsbCBoZXJlICh2YXJpYW5jZSBkb2VzIG5vdCBkaWZmZXIgYmV0d2VlbiBncm91cHMpLgoKIyMjIE9uZS13YXkgQU5PVkEgCgpHaXZlbiB0aGUgbnVsbCBvZiBtZWFuIGVxdWFsaXR5LCAKd2hhdCBpcyB0aGUgbGlrZWxpaG9vZCBvZiBvYnRhaW5pbmcgdGhlc2UgcmVzdWx0cz8KCmBgYHtyfQphbm92YSA8LSBhb3YoZGVuc2l0eV9tZWFuIH4gZWRlMSwgZGF0YSA9IHNsYTFfZGVuc2l0eV9zdGF0cykKdGlkeShhbm92YSkgJT4lIGthYmxlKCkKYGBgCgpPbmUtd2F5IEFOT1ZBIHJlamVjdHMgdGhlIG51bGwgd2l0aCAkcCA9IDEwXnstN30kLgoKIyMjIyBEaWFnbm9zdGljIHBsb3RzCgpgYGB7cn0KaGlzdChyZXNpZHVhbHMoYW5vdmEpKQpwbG90KGFub3ZhKSAKYGBgCgpUaGUgcmVzaWR1YWxzIGxvb2sgYXBwcm94aW1hdGVseSBub3JtYWxseSBkaXN0cmlidXRlZCAoaGlzdG9ncmFtLCBRLVEgcGxvdCkKd2l0aCBzaW1pbGFyIHZhcmlhbmNlIChSZXNpZHVhbHMgdnMuIEZpdHRlZCwgZ3JvdXBlZCBieSBmYWN0b3IpLgoKIyMjIFBvc3QtaG9jIHRlc3QgKFR1a2V5KQoKRm9sbG93aW5nIHRoZSByZWplY3Rpb24gb2YgdGhlIG51bGwgYnkgQU5PVkEsIHdlIGNhbiB1c2UgVHVrZXktS3JhbWVyCnRvIGNoZWNrIHBhcml3c2UgY29tcGFyaXNvbnMuIAoKYGBge3J9CnR1a2V5ICU+JQogIGthYmxlKCkKYGBgCgpUd28gY29tcGFyaXNvbnMgZG8gbm90IHByb2R1Y2Ugc3RhdGlzdGljYWxseSBzaWduaWZpY2FudCBkaWZmZXJlbmNlczoKCiogYmV0d2VlbiBlZGUx4oiGUFEgYW5kIGVkZTHiiIZDQwoqIGJldHdlZW4gZWRlMeKIhlBRQ0MgYW5kIGVkZTHiiIYKCkZvciBhbGwgb3RoZXIgZ3JvdXBzLCAkcCA8IDAuMDUkIChhdCBsZWFzdCk7CmZvciBhbGwgY29tcGFyaXNvbnMgd2l0aCB3aWxkIHR5cGUgJHAgPCAwLjAwMSQuCgojIyBPdmVyYWxsIHN1bW1hcnkKClN1bW1hcnkgc3RhdGlzdGljcyBmb3IgYWxsIGV4cGVyaW1lbnRzLCBkZXJpdmVkIGZyb20gKm1lYW4gdmFsdWVzKgpvZiBOIGluZGVwZW5kZW50IHJlcGVhdHMuCgojIyMgRmluYWwgZXN0aW1hdGVzCgpGaW5hbCBlc3RpbWF0ZXMgd2l0aCBsb3dlciAvIHVwcGVyIDk1JSBjb25maWRlbmNlIGludGVydmFscyBhbmQgYSBjb21wYXJpc29uCnRvIHdpbGQgdHlwZSAoaW4gJSkuIGBoYWxmX2NpYCBpcyBqdXN0IHRoZSBlcnJvciBmb3Igd3JpdGluZyBDSSByYW5nZXMKaW4gdGhlIGZvcm1hdCBtZWFuICsvLSBlcnJvci4KCmBgYHtyfQp3dF9tZWFuIDwtIHNsYTFfZGVuc2l0eV9zdGF0cyAlPiUKICBmaWx0ZXIoZWRlMSA9PSAnd3QnKSAlPiUKICBwdWxsKGRlbnNpdHlfbWVhbikgJT4lCiAgbWVhbigpCgpkZW5zaXR5X2NpIDwtIHNsYTFfZGVuc2l0eV9zdGF0cyAlPiUKICBncm91cF9ieShlZGUxKSU+JQogIHN1bW1hcmlzZShtZWFuX2NsX25vcm1hbChkZW5zaXR5X21lYW4pKSAlPiUKICByZW5hbWUobWVhbiA9IHksIGxvd2VyID0geW1pbiwgdXBwZXIgPSB5bWF4KSAlPiUKICBtdXRhdGUocHJvY193dCA9IHJvdW5kKDEwMCAqIG1lYW4gLyB3dF9tZWFuKSwKICAgICAgICAgaGFsZl9jaSA9ICh1cHBlciAtIGxvd2VyKSAvIDIpIAoKa2FibGUoZGVuc2l0eV9jaSwgZGlnaXRzID0gMykKYGBgCgojIyMgQ29uY2x1c2lvbnMKCjEuIEFsbCBtdXRhdGlvbnMgY2F1c2UgYSBzaWduaWZpY2FudCByZWR1Y3Rpb24gaW4gcGF0Y2ggZGVuc2l0eSBmcm9tIHdpbGQgdHlwZQoyLiBFZGUx4oiGUFFDQyBpcyBpbmRpc3Rpbmd1aXNoYWJsZSBmcm9tIGZ1bGwgRWRlMSBkZWxldGlvbiwgCiAgY2F1c2VzIH41MCUgcmVkdWN0aW9uIGluIGRlbnNpdHkKMy4gSW5kaXZpZHVhbCBQUSAvIENDIGRlbGV0aW9ucyBoYXZlIGludGVybWVkaWF0ZSBkZWZlY3RzCgojIyMgTW9yZSBzdGF0aXN0aWNzCgpgYGB7cn0Kc2xhMV9kZW5zaXR5X3N0YXRzICU+JQogIGdyb3VwX2J5KGVkZTEpJT4lCiAgc3VtbWFyaXNlKE4gPSBuKCksCiAgICAgICAgICAgIGFjcm9zcyhkZW5zaXR5X21lYW4sCiAgICAgICAgICAgICAgICAgICBsaXN0KG1lYW4gPSBtZWFuLCBzZCA9IHNkLAogICAgICAgICAgICAgICAgICAgICAgICBzZSA9IH4gc2QoLngpIC8gc3FydChuKCkpLAogICAgICAgICAgICAgICAgICAgICAgICBtZWRpYW4gPSBtZWRpYW4sIG1hZCA9IG1hZCksCiAgICAgICAgICAgICAgICAgICAgICAgLm5hbWVzID0gJ3suZm59JyksCiAgICAgICAgICAgIC5ncm91cHMgPSAnZHJvcCcpICU+JQogIGthYmxlKGRpZ2l0cyA9IDMpCmBgYAoKIyMjIE1vcmUgc3RhdGlzdGljcyAob2JzZXJ2YXRpb24tbGV2ZWwpCgpTbyBmYXIgd2UgaGF2ZSBtb3N0bHkgbG9va2VkIGF0IHRoZSBzdGF0aXN0aWNzIApkZXJpdmVkIGZyb20gZXhwZXJpbWVudC1sZXZlbCBtZWFucy4KRm9yIGNvbXBsZXRlbmVzcywgdGhlIHRhYmxlIGJlbG93CnJlcG9ydHMgbnVtYmVyIG9mIG9ic2VydmF0aW9ucyBhbmQgZGVuc2l0eSBzdGF0aXN0aWMgCmRlcml2ZWQgZnJvbSBwb29sZWQgb2JzZXJ2YXRpb25zIGZyb20gYWxsIHJlcGVhdHMsCmluIGVhY2ggZ3JvdXA6CgpgYGB7cn0Kc2xhMV9kZW5zaXR5ICU+JQogIGdyb3VwX2J5KGVkZTEpJT4lCiAgc3VtbWFyaXNlKG4gPSBuKCksCiAgICAgICAgICAgIGFjcm9zcyhkZW5zaXR5LAogICAgICAgICAgICAgICAgICAgbGlzdChtZWFuID0gbWVhbiwgc2QgPSBzZCwKICAgICAgICAgICAgICAgICAgICAgICAgc2UgPSB+IHNkKC54KSAvIHNxcnQobigpKSwKICAgICAgICAgICAgICAgICAgICAgICAgbWVkaWFuID0gbWVkaWFuLCBtYWQgPSBtYWQKICAgICAgICAgICAgICAgICAgICAgICAgIywgcXVhbnQgPSB+cXVhbnRpbGUoLngsIGMoMC4yNSwgMC41LCAwLjc1KSkKICAgICAgICAgICAgICAgICAgICAgICAgKSwKICAgICAgICAgICAgICAgICAgICAgICAubmFtZXMgPSAney5mbn0nKSwKICAgICAgICAgICAgcGl2b3Rfd2lkZXIoZW5mcmFtZShxdWFudGlsZShkZW5zaXR5LCBjKDAuMjUsIDAuNzUpKSkpLAogICAgICAgICAgICAuZ3JvdXBzID0gJ2Ryb3AnKSAlPiUKICAKICBrYWJsZShkaWdpdHMgPSAzKQpgYGAKCiMjIFNvdXJjZSBkYXRhCgojIyMgLmNzdgoKYGBge3IgZWNobz1GQUxTRX0KeGZ1bjo6ZW1iZWRfZmlsZSgnZGF0YS9zbGExX2RlbnNpdHkuY3N2JykKYGBgCgojIyMgLlJEYXRhCgpgYGB7ciBlY2hvPUZBTFNFfQp4ZnVuOjplbWJlZF9maWxlKCdkYXRhL3NsYTFfZGVuc2l0eS5SRGF0YScpCmBgYAoKIyMgU2Vzc2lvbiBpbmZvCgpgYGB7ciBzZXNzaW9uLCBtZXNzYWdlPVRSVUV9CnNlc3Npb25JbmZvKCkKYGBgCg==
