# Supplementary material for: Condensation of Ede1 promotes the initiation of endocytosis
Source: eLife. 2022 Apr 12;11:e72865. doi: 10.7554/eLife.72865 (PMC9064294; doi:10.7554/eLife.72865)
Supplement: Figure 6—source data 2. [file elife-72865-fig6-data2.zip › figure6_data2.html]

Sla1 lifetime in Ede1 internal deletion mutants


Code 

- Download Rmd

# Sla1 lifetime in Ede1 internal deletion mutants

#### Last compiled on July 30, 2021

# 

## Experiment

### Rationale

The aim of the experiment is to determine patch initiation rates in Ede1 internal domain deletion mutants. We looked at the patch density and lifetimes of late coat protein Sla1 in Ede1 mutants lacking all or some of the central region. This notebook is devoted to the Sla1 patch lifetime.

### Acquisition and replicates

All lifetime estimates come from movies acquired on the Olympus IX83 with a 150x/1.45 objective. The illumination was CoolLED’s pE-300 lamp with a GFP filter cube. Camera was Hammamatsu’s ImageEMX2 EMCCD.

The first dataset (#0 in this notebook) was acquired with 25% power and 200 ms exposure times. For the three subsequent datasets, the lamp power was reduced to 15%, and exposure was increased to 500 ms. Despite this difference, the result from the first, exploratory dataset looks in line with the rest of the repeats, so I decided to include it in the analysis.

### Processing and data extraction

All images were background subtracted using ImageJ rolling ball algorithm with 80 px radius, and normalized to correct for photobleaching. Individual cells were cropped out and median-filtered image (6px disk brush) was subtracted. ParticleTracker from the MOSAIC Suite was used to track the spots. Individual tracks were manually selected based on the quality of the tracking.

Another notebook was used to gather all output into tidy data frames with no further modifications.

### List of strains used

| strain | ede1 |
| --- | --- |
| MKY0140 | wt |
| MKY3770 | pq |
| MKY3776 | cc |
| MKY3782 | pqcc |
| MKY0654 | delta |

## Per-dataset summary

Basic summary of each dataset: exact sample sizes, mean, sd, se, median, MAD.

| ede1 | dataset | n | lifetime\_mean | lifetime\_sd | lifetime\_se | lifetime\_median | lifetime\_mad |
| --- | --- | --- | --- | --- | --- | --- | --- |
| wt | 0 | 49 | 22.51184 | 6.264182 | 0.8948831 | 22.080 | 5.455968 |
| wt | 1 | 92 | 24.98370 | 6.769539 | 0.7057732 | 23.750 | 6.671700 |
| wt | 2 | 61 | 22.90164 | 6.952709 | 0.8902032 | 22.000 | 4.447800 |
| wt | 3 | 82 | 22.23171 | 6.700007 | 0.7398920 | 21.500 | 5.559750 |
| pq | 0 | 58 | 19.09397 | 4.435555 | 0.5824168 | 18.630 | 3.409980 |
| pq | 1 | 94 | 20.31915 | 5.703635 | 0.5882851 | 20.000 | 5.930400 |
| pq | 2 | 60 | 19.57500 | 7.816662 | 1.0091268 | 19.000 | 7.042350 |
| pq | 3 | 77 | 18.33766 | 5.593959 | 0.6374908 | 17.500 | 5.930400 |
| cc | 0 | 69 | 17.68667 | 4.399831 | 0.5296774 | 17.020 | 2.727984 |
| cc | 1 | 95 | 18.50526 | 5.158908 | 0.5292928 | 17.500 | 5.189100 |
| cc | 2 | 62 | 19.00806 | 5.240520 | 0.6655467 | 18.750 | 4.818450 |
| cc | 3 | 79 | 16.89241 | 6.307134 | 0.7096080 | 17.000 | 5.189100 |
| pqcc | 0 | 66 | 16.55652 | 4.510219 | 0.5551695 | 15.755 | 3.921477 |
| pqcc | 1 | 100 | 18.39000 | 5.278401 | 0.5278401 | 17.000 | 5.189100 |
| pqcc | 2 | 62 | 16.19355 | 5.228669 | 0.6640416 | 16.000 | 4.818450 |
| pqcc | 3 | 74 | 15.73649 | 5.200252 | 0.6045170 | 14.500 | 4.447800 |
| delta | 0 | 61 | 15.34213 | 4.225024 | 0.5409589 | 14.720 | 4.432974 |
| delta | 1 | 100 | 18.09000 | 4.979443 | 0.4979443 | 18.000 | 5.189100 |
| delta | 2 | 61 | 17.10656 | 5.922496 | 0.7582979 | 16.000 | 5.189100 |
| delta | 3 | 78 | 15.27564 | 4.611285 | 0.5221251 | 15.000 | 5.189100 |

## Plots

### SuperPlots

Large, coloured points represent mean values from each independent experiment. The center line and range represent the mean +/- SD, calculated based on the experimental averages.

Beeswarm points represent all underlying observations, shaped according to the dataset.

Violin instead of beeswarm to summarize all observations:

### With significance

Let’s add significance stars based on Tukey’s test.

Like with density, this view is getting complicated even though it’s only half of the comparisons.

We can simplify it down to binary comparisons at a given α level (here, α = 0.95). We can reject the null of group mean equality at this level for groups which do not share any letters between them.

## Hypothesis tests

### Assumptions

ANOVA and similar parametric tests assume that the errors are normally distributed, with homogeneous variances, and that the samples are independent. We will test the null hypothesis that mean Sla1 lifetime is the same across different Ede1 strains.

We will use repeat-level data for the tests to account for experimental variability. Also, even if the populations are skewed (as it seems from the plots), the sample means should still be normally distributed (according to the Central Limit Theorem).

#### Normality

From the plots it looks like the underlying data is not perfectly normal with some skew. We can check the normality of residuals used in the model later, but it might still be interesting to know how normal the underlying data is overall.

If we do a formal test (Shapiro-Wilkes):

| ede1 | n | p.value |
| --- | --- | --- |
| wt | 284 | 2.5e-06 |
| pq | 289 | 0.0e+00 |
| cc | 305 | 0.0e+00 |
| pqcc | 302 | 2.2e-06 |
| delta | 300 | 5.3e-06 |

Shapiro-Wilkes rejects the normality of the data in each group. That is about expected with a large sample size, but it probably also reflects an actual skew in lifetimes.

Q-Q plots:

All datasets do indeed look heavy-tailed. Histograms:

#### Homoscedasticity

4 points per group is probably enough to assess whether the variance is similar in the repeat-level data. Levene’s test:

| df1 | df2 | statistic | p |
| --- | --- | --- | --- |
| 4 | 15 | 0.3414199 | 0.8457886 |

Levene’s cannot reject the null here (variance does not differ between groups).

### One-way ANOVA

| term | df | sumsq | meansq | statistic | p.value |
| --- | --- | --- | --- | --- | --- |
| ede1 | 4 | 118.74850 | 29.687125 | 23.27211 | 2.8e-06 |
| Residuals | 15 | 19.13478 | 1.275652 | NA | NA |

#### Diagnostic plots

Again, the variance looks homogeneous enough. There is a definite departure from normality as well, although I am not sure how concerning it really is.

### Post-hoc test (Tukey)

| term | group1 | group2 | null.value | estimate | conf.low | conf.high | p.adj | p.adj.signif |
| --- | --- | --- | --- | --- | --- | --- | --- | --- |
| ede1 | wt | pq | 0 | -3.8257756 | -6.291916 | -1.3596350 | 1.89e-03 | \*\* |
| ede1 | wt | cc | 0 | -5.1341199 | -7.600261 | -2.6679793 | 9.59e-05 | \*\*\*\* |
| ede1 | wt | pqcc | 0 | -6.4380823 | -8.904223 | -3.9719417 | 6.80e-06 | \*\*\*\* |
| ede1 | wt | delta | 0 | -6.7036374 | -9.169778 | -4.2374968 | 4.10e-06 | \*\*\*\* |
| ede1 | pq | cc | 0 | -1.3083443 | -3.774485 | 1.1577963 | 4.97e-01 | ns |
| ede1 | pq | pqcc | 0 | -2.6123067 | -5.078447 | -0.1461661 | 3.55e-02 | \* |
| ede1 | pq | delta | 0 | -2.8778618 | -5.344002 | -0.4117212 | 1.88e-02 | \* |
| ede1 | cc | pqcc | 0 | -1.3039623 | -3.770103 | 1.1621783 | 5.01e-01 | ns |
| ede1 | cc | delta | 0 | -1.5695175 | -4.035658 | 0.8966231 | 3.28e-01 | ns |
| ede1 | pqcc | delta | 0 | -0.2655551 | -2.731696 | 2.2005855 | 9.97e-01 | ns |

All mutants are significantly different than wild-type, but not necessarily between themselves. Most notably, we do not have enough power to say if ∆CC is different from ∆PQ, ∆PQCC or ede1∆. At the same time, ∆PQ difference from ∆PQCC and ede1∆ reaches the significance threshold.

## Overall summary

Summary statistics for all experiments, derived from *mean values* of N independent repeats.

### Final estimates

Final estimates with lower / upper 95% confidence intervals and a comparison to wild type (in %). `half_ci` is just the error for writing CI ranges in the format mean +/- error.

| ede1 | mean | lower | upper | proc\_wt | half\_ci |
| --- | --- | --- | --- | --- | --- |
| wt | 23.157 | 21.171 | 25.143 | 100 | 1.986 |
| pq | 19.331 | 18.007 | 20.656 | 83 | 1.325 |
| cc | 18.023 | 16.543 | 19.503 | 78 | 1.480 |
| pqcc | 16.719 | 14.868 | 18.570 | 72 | 1.851 |
| delta | 16.454 | 14.255 | 18.652 | 71 | 2.199 |

### Conclusions

1. All mutations cause a significant reduction in Sla1 patch lifetime from wild type
2. Ede1∆PQCC is indistinguishable from full Ede1 deletion, causes ~30% reduction in lifetime
3. Individual PQ / CC deletions have intermediate defects

### More statistics

| ede1 | N | mean | sd | se | median | mad |
| --- | --- | --- | --- | --- | --- | --- |
| wt | 4 | 23.157 | 1.248 | 0.624 | 22.707 | 0.497 |
| pq | 4 | 19.331 | 0.832 | 0.416 | 19.334 | 0.908 |
| cc | 4 | 18.023 | 0.930 | 0.465 | 18.096 | 0.980 |
| pqcc | 4 | 16.719 | 1.163 | 0.582 | 16.375 | 0.608 |
| delta | 4 | 16.454 | 1.382 | 0.691 | 16.224 | 1.357 |

### More statistics (observation-level)

It might be useful to also look at observation-level summary. The experimental means can be used to determine true population mean (because of the CLT), but if the population is really skewed, median and quartiles are particularly useful information which cannot be accurately assessed from only 4 points.

| ede1 | n | mean | sd | se | median | mad | 25% | 75% |
| --- | --- | --- | --- | --- | --- | --- | --- | --- |
| wt | 284 | 23.315 | 6.774 | 0.402 | 22.50 | 5.930 | 19.0 | 27.035 |
| pq | 289 | 19.391 | 5.981 | 0.352 | 18.63 | 5.382 | 15.5 | 22.500 |
| cc | 305 | 18.005 | 5.376 | 0.308 | 17.50 | 4.448 | 14.5 | 21.000 |
| pqcc | 302 | 16.888 | 5.182 | 0.298 | 16.00 | 5.189 | 13.0 | 20.353 |
| delta | 300 | 16.600 | 5.087 | 0.294 | 16.00 | 5.189 | 13.0 | 19.608 |

## Source data

### .csv

Download sla1\_lifetime.csv

### .RData

Download sla1\_lifetime.RData

## Session info

```
## R version 4.1.0 (2021-05-18)
## Platform: x86_64-apple-darwin17.0 (64-bit)
## Running under: macOS Mojave 10.14.6
## 
## Matrix products: default
## BLAS:   /Library/Frameworks/R.framework/Versions/4.1/Resources/lib/libRblas.dylib
## LAPACK: /Library/Frameworks/R.framework/Versions/4.1/Resources/lib/libRlapack.dylib
## 
## locale:
## [1] en_US.UTF-8/en_US.UTF-8/en_US.UTF-8/C/en_US.UTF-8/en_US.UTF-8
## 
## attached base packages:
## [1] stats     graphics  grDevices utils     datasets  methods   base     
## 
## other attached packages:
##  [1] multcompView_0.1-8 knitr_1.33         rstatix_0.7.0      broom_0.7.6       
##  [5] ggsignif_0.6.1     ggbeeswarm_0.6.0   forcats_0.5.1      stringr_1.4.0     
##  [9] dplyr_1.0.6        purrr_0.3.4        readr_1.4.0        tidyr_1.1.3       
## [13] tibble_3.1.2       ggplot2_3.3.3      tidyverse_1.3.1   
## 
## loaded via a namespace (and not attached):
##  [1] fs_1.5.0            lubridate_1.7.10    RColorBrewer_1.1-2 
##  [4] httr_1.4.2          tools_4.1.0         backports_1.2.1    
##  [7] utf8_1.2.1          R6_2.5.0            rpart_4.1-15       
## [10] vipor_0.4.5         Hmisc_4.5-0         DBI_1.1.1          
## [13] colorspace_2.0-1    nnet_7.3-16         withr_2.4.2        
## [16] gridExtra_2.3       tidyselect_1.1.1    curl_4.3.1         
## [19] compiler_4.1.0      cli_2.5.0           rvest_1.0.0        
## [22] htmlTable_2.2.1     xml2_1.3.2          labeling_0.4.2     
## [25] checkmate_2.0.0     scales_1.1.1        digest_0.6.27      
## [28] foreign_0.8-81      rmarkdown_2.8       rio_0.5.26         
## [31] base64enc_0.1-3     jpeg_0.1-8.1        pkgconfig_2.0.3    
## [34] htmltools_0.5.1.1   dbplyr_2.1.1        highr_0.9          
## [37] htmlwidgets_1.5.3   rlang_0.4.11        readxl_1.3.1       
## [40] rstudioapi_0.13     farver_2.1.0        generics_0.1.0     
## [43] jsonlite_1.7.2      zip_2.2.0           car_3.0-10         
## [46] magrittr_2.0.1      Formula_1.2-4       Matrix_1.3-3       
## [49] Rcpp_1.0.6          munsell_0.5.0       fansi_0.5.0        
## [52] abind_1.4-5         lifecycle_1.0.0     stringi_1.6.2      
## [55] yaml_2.2.1          carData_3.0-4       grid_4.1.0         
## [58] crayon_1.4.1        lattice_0.20-44     haven_2.4.1        
## [61] splines_4.1.0       hms_1.1.0           pillar_1.6.1       
## [64] reprex_2.0.0        glue_1.4.2          evaluate_0.14      
## [67] latticeExtra_0.6-29 data.table_1.14.0   modelr_0.1.8       
## [70] vctrs_0.3.8         png_0.1-7           cellranger_1.1.0   
## [73] gtable_0.3.0        assertthat_0.2.1    xfun_0.23          
## [76] openxlsx_4.2.3      mime_0.10           survival_3.2-11    
## [79] beeswarm_0.3.1      cluster_2.1.2       ellipsis_0.3.2
```

LS0tCnRpdGxlOiAiU2xhMSBsaWZldGltZSBpbiBFZGUxIGludGVybmFsIGRlbGV0aW9uIG11dGFudHMiCmRhdGU6ICJMYXN0IGNvbXBpbGVkIG9uIGByIGZvcm1hdChTeXMudGltZSgpLCAnJUIgJWQsICVZJylgIgpvdXRwdXQ6IAogIGh0bWxfZG9jdW1lbnQ6CiAgICBjb2RlX2Rvd25sb2FkOiBUUlVFCmVkaXRvcl9vcHRpb25zOiAKICBjaHVua19vdXRwdXRfdHlwZTogaW5saW5lCi0tLQoKYGBge3Igc2V0dXAsIGluY2x1ZGU9RkFMU0V9CmtuaXRyOjpvcHRzX2NodW5rJHNldChlY2hvID0gRkFMU0UsIG1lc3NhZ2U9RkFMU0UsIHdhcm5pbmc9RkFMU0UsCiAgICAgICAgICAgICAgICAgICAgICBkcGkgPSA5NiwgZmlnLndpZHRoID0gNCwgZmlnLmhlaWdodCA9IDMpCmBgYAoKYGBge3IgbGlic30KbGlicmFyeSh0aWR5dmVyc2UpCmxpYnJhcnkoZ2diZWVzd2FybSkKbGlicmFyeShnZ3NpZ25pZikKbGlicmFyeShicm9vbSkKbGlicmFyeShyc3RhdGl4KQpsaWJyYXJ5KGtuaXRyKQpsaWJyYXJ5KG11bHRjb21wVmlldykKYGBgCgpgYGB7ciBsb2FkfQojIExvYWQgZGF0YSBnZW5lcmF0ZWQgYnkgY2xlYW51cCBub3RlYm9vawpybShsaXN0ID0gbHMoKSkKbG9hZCgnZGF0YS9zbGExX2xpZmV0aW1lLlJEYXRhJykKYGBgCgpgYGB7ciB0aGVtZX0KIyBDdXN0b20gZ2dwbG90MiB0aGVtZQojIC0tLS0tLS0tLS0tLS0tLS0tLS0tCgojIG1pbmltYWwgdGhlbWUgd2l0aCBib3JkZXIKIyBiYXNlZCBvbiB0aGVtZV9saW5lZHJhdyB3aXRob3V0IHRoZSBncmlkIGxpbmVzCiMgYWxzbyB0cnlpbmcgdG8gcmVtb3ZlIGFsbCBiYWNrZ3JvdW5kcyBhbmQgbWFyZ2lucwojIHRoZSBhaW0gaXMgdG8gbWFrZSBpdCBhcyBlYXN5IGFzIHBvc3NpYmxlIHRvIGVkaXQgaW4gaWxsdXN0cmF0b3IKCnRoZW1lX2NsZWFuIDwtIGZ1bmN0aW9uKGJhc2Vfc2l6ZSA9IDExLCBiYXNlX2ZhbWlseSA9ICIiLAogICAgICAgICAgICAgICAgICAgICAgICBiYXNlX2xpbmVfc2l6ZSA9IGJhc2Vfc2l6ZSAvIDIyLAogICAgICAgICAgICAgICAgICAgICAgICBiYXNlX3JlY3Rfc2l6ZSA9IGJhc2Vfc2l6ZSAvIDIyKSB7CiAgdGhlbWVfbGluZWRyYXcoCiAgICBiYXNlX3NpemUgPSBiYXNlX3NpemUsCiAgICBiYXNlX2ZhbWlseSA9IGJhc2VfZmFtaWx5LAogICAgYmFzZV9saW5lX3NpemUgPSBiYXNlX2xpbmVfc2l6ZSwKICAgIGJhc2VfcmVjdF9zaXplID0gYmFzZV9yZWN0X3NpemUKICApICUrcmVwbGFjZSUKICAgIHRoZW1lKAogICAgICAjIG5vIGdyaWQgYW5kIG5vIGJhY2tncm91bmRzIGlmIEkgY2FuIGhlbHAgaXQKICAgICAgbGVnZW5kLmJhY2tncm91bmQgPSAgZWxlbWVudF9ibGFuaygpLAogICAgICBwYW5lbC5iYWNrZ3JvdW5kID0gZWxlbWVudF9ibGFuaygpLAogICAgICBwYW5lbC5ncmlkID0gZWxlbWVudF9ibGFuaygpLAogICAgICBwbG90LmJhY2tncm91bmQgPSBlbGVtZW50X2JsYW5rKCksCiAgICAgIHBsb3QubWFyZ2luID0gbWFyZ2luKDAsIDAsIDAsIDApLAogICAgICBjb21wbGV0ZSA9IFRSVUUKICAgICkKfQoKIyBTZXQgZGVmYXVsdCB0aGVtZQojIC0tLS0tLS0tLS0tLS0tLS0tCnRoZW1lX3NldCh0aGVtZV9jbGVhbihiYXNlX3NpemUgPSAxMiwgYmFzZV9mYW1pbHkgPSAiTXlyaWFkIFBybyIpKQoKIyBDcmVhdGUgYSBnZ3NhdmUgd3JhcHBlcgojIC0tLS0tLS0tLS0tLS0tLS0tLS0tLS0tCgojIFRoaXMgd2F5IHdlIGNhbiBzZXQgYSBkZWZhdWx0IHNpemUgYW5kIGRldmljZSBmb3IgYWxsIHBsb3RzCm15X2dnc2F2ZSA8LSBmdW5jdGlvbihmaWxlbmFtZSwgcGxvdCA9IGxhc3RfcGxvdCgpLAogICAgICAgICAgICAgICAgICAgICAgZGV2aWNlID0gY2Fpcm9fcGRmLCB1bml0cyA9ICJtbSIsCiAgICAgICAgICAgICAgICAgICAgICB3aWR0aCA9IDEwMCwgaGVpZ2h0ID0gODAsIC4uLil7CiAgZ2dzYXZlKGZpbGVuYW1lID0gZmlsZW5hbWUsIHBsb3QgPSBwbG90LAogICAgICAgICBkZXZpY2UgPSBkZXZpY2UsIHVuaXRzID0gdW5pdHMsCiAgICAgICAgIGhlaWdodCA9IGhlaWdodCwgd2lkdGggPSB3aWR0aCwgIC4uLikKICB9CmBgYAoKYGBge3IgZnVuY3Rpb25zfQojIGNvdXBsZSBvZiBzbWFsbCBmdW5jdGlvbnMgZm9yIGV4dHJhY3RpbmcgY29tcGFyaXNvbnMKIyBpbnRvIGEgZm9ybWF0IHVuZGVyc3RhbmRhYmxlIGJ5IGdncGxvdAoKIycgQWRkIGxldHRlciBncm91cCBsYWJlbHMgZ2VuZXJhdGVkIGJ5IFR1a2V5J3MgSFNECiMnCiMnIFRoaXMgZnVuY3Rpb24gcGVyZm9ybXMgQU5PVkEgYW5kIFR1a2V5J3MgSFNELAojJyBleHRyYWN0cyB0aGUgbGV0dGVyIGxhYmVscyBhbmQgYXR0YWNoZXMgdGhlbQojJyB0byB0aGUgb3JpZ2luYWwgZGF0YS4KIycKIycgQHBhcmFtIHg6IGRmIG9yIHRpYmJsZSwgdGhlIGRhdGEgKGxvbmcgZm9ybWF0ISkKIycgQHBhcmFtIHl2YXI6IGNociwgbmFtZSBvZiB0aGUgZGVwZW5kZW50IHZhcmlhYmxlCiMnIEBwYXJhbSB4dmFyOiBjaHIsIG5hbWUgb2YgdGhlIGluZGVwZW5kZW50IHZhcmlhYmxlCiMnIEBwYXJhbSBhbHBoYTogZGJsLCBjb25maWRlbmNlIGxldmVsIHBhc3NlZCBvbiB0byBUdWtleSdzIHRlc3QKIycKYWRkX3R1a2V5X2xhYmVscyA8LSBmdW5jdGlvbih4LCB5dmFyLCB4dmFyLCBhbHBoYSA9IDAuOTUpewogIGFvdl9mb3JtIDwtIGZvcm11bGEocGFzdGUoeXZhciwgJ34nLCB4dmFyKSkKICBhbm92YSA8LSBhb3YoZm9ybXVsYSA9IGFvdl9mb3JtLCBkYXRhID0geCkKICB0dWtleSA8LSBUdWtleUhTRChhbm92YSwgd2hpY2ggPSB4dmFyLCBjb25mLmxldmVsID0gYWxwaGEpCiAgIyBFeHRyYWN0IGxhYmVscyBhbmQgZmFjdG9yIGxldmVscyBmcm9tIFR1a2V5IHBvc3QtaG9jIAogIHhfbGFiZWxzIDwtIGFzX3RpYmJsZSgKICAgIG11bHRjb21wTGV0dGVyczQoYW5vdmEsIHR1a2V5KVtbeHZhcl1dJExldHRlcnMsCiAgICByb3duYW1lcyA9IHh2YXIKICAgICkKICAKICBpZiAoaXMuZmFjdG9yKHhbW3h2YXJdXSkpIHsKICAgIHhfbGFiZWxzW1t4dmFyXV0gPC0gZmFjdG9yKAogICAgICB4X2xhYmVsc1tbeHZhcl1dLCBsZXZlbHMgPSBsZXZlbHMoeFtbeHZhcl1dKQogICAgKQogIH0KICAKICB4X2xhYmVscyA8LSByZW5hbWUoeF9sYWJlbHMsIHR1a2V5X2dyb3VwID0gdmFsdWUpCiAgeCA8LSBsZWZ0X2pvaW4oeCwgeF9sYWJlbHMsIGJ5ID0geHZhcikKICAKICByZXR1cm4oeCkKICB9CgoKIycgRXh0cmFjdCBjb21wYXJpc29ucyBmcm9tIHJzdGF0aXggdGlkeSB0ZXN0cwojJyAKIycgVGhpcyBmdW5jdGlvbiBzdWJzZXRzIHRoZSBzZWxlY3RlZCBjb21wYXJpc29ucyBpbiBhIFR1a2V5LAojJyBEdW5uIG9yIHNpbWlsYXIgdGVzdCBkb25lIGJ5IHJzdGF0aXguCiMnIEl0IGNvbnZlcnRzIGdyb3VwcyB0byBhIGxpc3Qgb2YgdmVjdG9ycyB0aGF0IGNhbiBiZSBwYXNzZWQgdG8gZ2VvbV9zaWduaWYKIycKIycKIycgQHBhcmFtIHg6IGRmIG9yIHRpYmJsZSwgdGhlIGNvbXBhcmlzb24gcmVzdWx0cwojJyBAcGFyYW0gcm93czogaW50ZWdlciB2ZWN0b3IsIHRoZSByb3dzIHdpdGggZGVzaXJlZCBjb21wYXJpc29ucwpleHRyYWN0X2NvbXBhcmlzb25zIDwtIGZ1bmN0aW9uKHgsIHJvd3MpewogIHhfc3Vic2V0IDwtIHhbcm93cyxdICU+JQogICAgLltucm93KC4pOjEsXQogIAogIHhfY29tcGFyaXNvbnMgPC0geF9zdWJzZXQgJT4lCiAgICBzZWxlY3QoZ3JvdXAxLCBncm91cDIpICU+JQogICAgdCgpICU+JQogICAgYXMuZGF0YS5mcmFtZSgpICU+JQogICAgYXMubGlzdAogIHhfYW5ub3RhdGlvbnMgPC0geF9zdWJzZXQkcC5hZGouc2lnbmlmICU+JQogICAgYXMudmVjdG9yKCkKICAKICBzaWduaWZpY2FuY2UgPC0gbGlzdCgKICAgIGNvbXBhcmlzb25zID0geF9jb21wYXJpc29ucywKICAgIGFubm90YXRpb25zID0geF9hbm5vdGF0aW9ucwogICkKICAKICByZXR1cm4oc2lnbmlmaWNhbmNlKQp9CmBgYAoKIyB7LnRhYnNldCAudGFic2V0LXBpbGxzfQoKIyMgRXhwZXJpbWVudAoKIyMjIFJhdGlvbmFsZQoKVGhlIGFpbSBvZiB0aGUgZXhwZXJpbWVudCBpcyB0byBkZXRlcm1pbmUgcGF0Y2ggaW5pdGlhdGlvbiByYXRlcyAKaW4gRWRlMSBpbnRlcm5hbCBkb21haW4gZGVsZXRpb24gbXV0YW50cy4KV2UgbG9va2VkIGF0IHRoZSBwYXRjaCBkZW5zaXR5IGFuZCBsaWZldGltZXMgb2YgbGF0ZSBjb2F0IHByb3RlaW4gU2xhMSAKaW4gRWRlMSBtdXRhbnRzIGxhY2tpbmcgYWxsIG9yIHNvbWUgb2YgdGhlIGNlbnRyYWwgcmVnaW9uLgpUaGlzIG5vdGVib29rIGlzIGRldm90ZWQgdG8gdGhlIFNsYTEgcGF0Y2ggbGlmZXRpbWUuCgojIyMgQWNxdWlzaXRpb24gYW5kIHJlcGxpY2F0ZXMKCkFsbCBsaWZldGltZSBlc3RpbWF0ZXMgY29tZSBmcm9tIG1vdmllcyAKYWNxdWlyZWQgb24gdGhlIE9seW1wdXMgSVg4MyB3aXRoIGEgMTUweC8xLjQ1IG9iamVjdGl2ZS4KVGhlIGlsbHVtaW5hdGlvbiB3YXMgQ29vbExFROKAmXMgcEUtMzAwIGxhbXAgd2l0aCBhIEdGUCBmaWx0ZXIgY3ViZS4gCkNhbWVyYSB3YXMgSGFtbWFtYXRzdSdzIEltYWdlRU1YMiBFTUNDRC4KClRoZSBmaXJzdCBkYXRhc2V0ICgjMCBpbiB0aGlzIG5vdGVib29rKSAKd2FzIGFjcXVpcmVkIHdpdGggMjUlIHBvd2VyIGFuZCAyMDAgbXMgZXhwb3N1cmUgdGltZXMuIApGb3IgdGhlIHRocmVlIHN1YnNlcXVlbnQgZGF0YXNldHMsIAogIHRoZSBsYW1wIHBvd2VyIHdhcyByZWR1Y2VkIHRvIDE1JSwgCiAgYW5kIGV4cG9zdXJlIHdhcyBpbmNyZWFzZWQgdG8gNTAwIG1zLgpEZXNwaXRlIHRoaXMgZGlmZmVyZW5jZSwgdGhlIHJlc3VsdCBmcm9tIHRoZSBmaXJzdCwgZXhwbG9yYXRvcnkgZGF0YXNldCAKbG9va3MgaW4gbGluZSB3aXRoIHRoZSByZXN0IG9mIHRoZSByZXBlYXRzLApzbyBJIGRlY2lkZWQgdG8gaW5jbHVkZSBpdCBpbiB0aGUgYW5hbHlzaXMuCgojIyMgUHJvY2Vzc2luZyBhbmQgZGF0YSBleHRyYWN0aW9uCgpBbGwgaW1hZ2VzIHdlcmUgYmFja2dyb3VuZCBzdWJ0cmFjdGVkIAp1c2luZyBJbWFnZUogcm9sbGluZyBiYWxsIGFsZ29yaXRobSB3aXRoIDgwIHB4IHJhZGl1cywgCmFuZCBub3JtYWxpemVkIHRvIGNvcnJlY3QgZm9yIHBob3RvYmxlYWNoaW5nLgpJbmRpdmlkdWFsIGNlbGxzIHdlcmUgY3JvcHBlZCBvdXQgCmFuZCBtZWRpYW4tZmlsdGVyZWQgaW1hZ2UgKDZweCBkaXNrIGJydXNoKSB3YXMgc3VidHJhY3RlZC4gClBhcnRpY2xlVHJhY2tlciBmcm9tIHRoZSBNT1NBSUMgU3VpdGUgd2FzIHVzZWQgdG8gdHJhY2sgdGhlIHNwb3RzLgpJbmRpdmlkdWFsIHRyYWNrcyB3ZXJlIG1hbnVhbGx5IHNlbGVjdGVkIGJhc2VkIG9uIHRoZSBxdWFsaXR5IG9mIHRoZSB0cmFja2luZy4KCkFub3RoZXIgbm90ZWJvb2sgd2FzIHVzZWQgdG8gZ2F0aGVyIGFsbCBvdXRwdXQgaW50byB0aWR5IGRhdGEgZnJhbWVzCndpdGggbm8gZnVydGhlciBtb2RpZmljYXRpb25zLgoKIyMjIExpc3Qgb2Ygc3RyYWlucyB1c2VkCgpgYGB7ciBzdHJhaW5zfQprYWJsZShzdHJhaW5zKQpgYGAKCiMjIFBlci1kYXRhc2V0IHN1bW1hcnkKCkJhc2ljIHN1bW1hcnkgb2YgZWFjaCBkYXRhc2V0OiBleGFjdCBzYW1wbGUgc2l6ZXMsIG1lYW4sIHNkLCBzZSwgbWVkaWFuLCBNQUQuCgpgYGB7ciBzdGF0c30Kc2xhMV9saWZldGltZV9zdGF0cyA8LSBzbGExX2xpZmV0aW1lICU+JQogIGdyb3VwX2J5KGVkZTEsIGRhdGFzZXQpICU+JQogIHN1bW1hcmlzZShuID0gbigpLAogICAgICAgICAgICBhY3Jvc3MobGlmZXRpbWUsCiAgICAgICAgICAgICAgICAgICBsaXN0KG1lYW4gPSBtZWFuLCBzZCA9IHNkLCAKICAgICAgICAgICAgICAgICAgICAgICAgc2UgPSB+IHNkKC54KSAvIHNxcnQobigpKSwKICAgICAgICAgICAgICAgICAgICAgICAgbWVkaWFuID0gbWVkaWFuLCBtYWQgPSBtYWQpKSwKICAgICAgICAgICAgLmdyb3VwcyA9ICdkcm9wJykKCnNsYTFfbGlmZXRpbWVfc3RhdHMgJT4lIGthYmxlKCkKYGBgCgojIyBQbG90cyB7LnRhYnNldH0KCmBgYHtyIGxpZmV0aW1lLnNjYXR0ZXJ9CnBsb3RfYmxhbmsgPC0gZ2dwbG90KHNsYTFfbGlmZXRpbWVfc3RhdHMsCiAgICAgICAgICAgICAgICAgICAgIGFlcyh4ID0gZWRlMSwgeSA9IGxpZmV0aW1lX21lYW4pKSArCiAgICAgICAgICAgICAgICAgICAgICAgICAjc2hhcGUgPSBkYXRhc2V0LCBmaWxsID0gZGF0YXNldCkpKwogIGxhYnModGl0bGUgPSBOVUxMLCB4ID0gJ0VkZTEnLCB5ID0gIlNsYTEgbGlmZXRpbWUgKHMpIikgKwogIHNjYWxlX3lfY29udGludW91cyhicmVha3MgPSBzY2FsZXM6OmJyZWFrc19leHRlbmRlZCg2KSkrCiAgc2NhbGVfc2hhcGVfbWFudWFsKHZhbHVlcyA9IGMoMjE6MjUpKSArCiAgc2NhbGVfY29sb3JfYnJld2VyKHBhbGV0dGUgPSAnU2V0MicpICsKICBzY2FsZV9maWxsX2JyZXdlcihwYWxldHRlID0gJ1NldDInKQoKcGxvdF9zY2F0dGVyIDwtIHBsb3RfYmxhbmsgKwogIGdlb21fcXVhc2lyYW5kb20oaW5oZXJpdC5hZXMgPSBGLCBkYXRhID0gc2xhMV9saWZldGltZSwKICAgICAgICAgICAgICAgICAgIGFlcyh4ID0gZWRlMSwgeSA9IGxpZmV0aW1lLAogICAgICAgICAgICAgICAgICAgICAgIHNoYXBlID0gZGF0YXNldCwjIGNvbG91ciA9IGRhdGFzZXQKICAgICAgICAgICAgICAgICAgICAgICApLAogICAgICAgICAgICAgICAgICAgY29sb3VyID0gJ2dyZXk3NScsIyBzaGFwZSA9IDEsCiAgICAgICAgICAgICAgICAgICBzaG93LmxlZ2VuZCA9IEYsIHNpemUgPSAwLjgKICAgICAgICAgICAgICAgICAgICkKCnBsb3RfdmlvbGluIDwtIHBsb3RfYmxhbmsgKyAKICBnZW9tX3Zpb2xpbihpbmhlcml0LmFlcyA9IEYsCiAgICAgICAgICAgICAgZGF0YSA9IHNsYTFfbGlmZXRpbWUsIGFlcyh4ID0gZWRlMSwgeSA9IGxpZmV0aW1lKSwKICAgICAgICAgICAgICBjb2xvdXIgPSAnZ3JleTc1JywgZmlsbCA9ICd0cmFuc3BhcmVudCcKICAgICAgICAgICAgICApCmBgYAoKIyMjIFN1cGVyUGxvdHMKCkxhcmdlLCBjb2xvdXJlZCBwb2ludHMgcmVwcmVzZW50IG1lYW4gdmFsdWVzIApmcm9tIGVhY2ggaW5kZXBlbmRlbnQgZXhwZXJpbWVudC4KVGhlIGNlbnRlciBsaW5lIGFuZCByYW5nZSByZXByZXNlbnQgdGhlIG1lYW4gKy8tIFNELCAKY2FsY3VsYXRlZCBiYXNlZCBvbiB0aGUgZXhwZXJpbWVudGFsIGF2ZXJhZ2VzLgoKQmVlc3dhcm0gcG9pbnRzIHJlcHJlc2VudCBhbGwgdW5kZXJseWluZyBvYnNlcnZhdGlvbnMsIApzaGFwZWQgYWNjb3JkaW5nIHRvIHRoZSBkYXRhc2V0LgoKYGBge3J9CnBsb3Rfc3VwZXIgPC0gcGxvdF9zY2F0dGVyICsKICBnZW9tX3F1YXNpcmFuZG9tKGFlcyhzaGFwZSA9IGRhdGFzZXQsIGZpbGwgPSBkYXRhc2V0KSwKICAgICAgICAgICAgICAgICAgIHNob3cubGVnZW5kID0gRiwKICAgICAgICAgICAgICAgICAgIHdpZHRoID0gMC4zLCBzaXplID0gMikrCiAgc3RhdF9zdW1tYXJ5KGZ1biA9IG1lYW4sIGdlb20gPSAnY3Jvc3NiYXInLAogICAgICAgICAgICAgICB3aWR0aCA9IDAuNSwgZmF0dGVuID0gMSkrCiAgc3RhdF9zdW1tYXJ5KGZ1bi5kYXRhID0gJ21lYW5fc2RsJywKICAgICAgICAgICAgICAgZnVuLmFyZ3MgPSBsaXN0KG11bHQgPSAxKSwgCiAgICAgICAgICAgICAgIGdlb20gPSAnZXJyb3JiYXInLCB3aWR0aCA9IDAuMikKICAKcHJpbnQocGxvdF9zdXBlcikKbXlfZ2dzYXZlKCdmaWd1cmVzL2xpZmV0aW1lX3N1cGVyLnBkZicpCmBgYAoKVmlvbGluIGluc3RlYWQgb2YgYmVlc3dhcm0gdG8gc3VtbWFyaXplIGFsbCBvYnNlcnZhdGlvbnM6CgpgYGB7cn0KcGxvdF9zdXBlcl92aW9saW4gPC0gcGxvdF92aW9saW4gKwogIGdlb21fcXVhc2lyYW5kb20oYWVzKHNoYXBlID0gZGF0YXNldCwgZmlsbCA9IGRhdGFzZXQpLAogICAgICAgICAgICAgICAgICAgc2hvdy5sZWdlbmQgPSBGLAogICAgICAgICAgICAgICAgICAgd2lkdGggPSAwLjMsIHNpemUgPSAyKSsKICBzdGF0X3N1bW1hcnkoZnVuID0gbWVhbiwgZ2VvbSA9ICdjcm9zc2JhcicsCiAgICAgICAgICAgICAgIHdpZHRoID0gMC41LCBmYXR0ZW4gPSAxKSsKICBzdGF0X3N1bW1hcnkoZnVuLmRhdGEgPSAnbWVhbl9zZGwnLAogICAgICAgICAgICAgICBmdW4uYXJncyA9IGxpc3QobXVsdCA9IDEpLAogICAgICAgICAgICAgICBnZW9tID0gJ2Vycm9yYmFyJywgd2lkdGggPSAwLjIpCgpwcmludChwbG90X3N1cGVyX3Zpb2xpbikKbXlfZ2dzYXZlKCdmaWd1cmVzL2xpZmV0aW1lX3N1cGVyX3Zpb2xpbi5wZGYnKQpgYGAKCiMjIyBXaXRoIHNpZ25pZmljYW5jZQoKTGV0J3MgYWRkIHNpZ25pZmljYW5jZSBzdGFycyBiYXNlZCBvbiBUdWtleSdzIHRlc3QuCgpgYGB7cn0KdHVrZXkgPC0gdHVrZXlfaHNkKHNsYTFfbGlmZXRpbWVfc3RhdHMsIGxpZmV0aW1lX21lYW4gfiBlZGUxLAogICAgICAgICAgICAgICAgICAgb3JkZXJlZCA9IFRSVUUpCgpzaWduaWZpY2FuY2UgPC0gZXh0cmFjdF9jb21wYXJpc29ucyh0dWtleSwgYygxOjQsIDEwKSkKCnBsb3Rfc3VwZXJfc2lnbmlmIDwtIHBsb3Rfc3VwZXIgKwogIGdlb21fc2lnbmlmKGNvbXBhcmlzb25zID0gc2lnbmlmaWNhbmNlJGNvbXBhcmlzb25zLAogICAgICAgICAgICAgIGFubm90YXRpb25zID0gc2lnbmlmaWNhbmNlJGFubm90YXRpb25zLAogICAgICAgICAgICAgIHN0ZXBfaW5jcmVhc2UgPSAwLjAzLAogICAgICAgICAgICAgIHRpcF9sZW5ndGggPSAwLjAxLCB2anVzdCA9IDAuOCwKICAgICAgICAgICAgICBtYXJnaW5fdG9wID0gLTAuMSkKcHJpbnQocGxvdF9zdXBlcl9zaWduaWYpCm15X2dnc2F2ZSgnZmlndXJlcy9saWZldGltZV9zdXBlcl9zaWduaWYucGRmJykKYGBgCgpMaWtlIHdpdGggZGVuc2l0eSwgdGhpcyB2aWV3IGlzIGdldHRpbmcgY29tcGxpY2F0ZWQKZXZlbiB0aG91Z2ggaXQncyBvbmx5IGhhbGYgb2YgdGhlIGNvbXBhcmlzb25zLgoKV2UgY2FuIHNpbXBsaWZ5IGl0IGRvd24gdG8gYmluYXJ5IGNvbXBhcmlzb25zIGF0IGEgZ2l2ZW4gzrEgbGV2ZWwgKGhlcmUsIM6xID0gMC45NSkuCldlIGNhbiByZWplY3QgdGhlIG51bGwgb2YgZ3JvdXAgbWVhbiBlcXVhbGl0eSBhdCB0aGlzIGxldmVsCmZvciBncm91cHMgd2hpY2ggZG8gbm90IHNoYXJlIGFueSBsZXR0ZXJzIGJldHdlZW4gdGhlbS4KCmBgYHtyfQpzbGExX2xpZmV0aW1lX3N0YXRzIDwtIHNsYTFfbGlmZXRpbWVfc3RhdHMgJT4lCiAgYWRkX3R1a2V5X2xhYmVscygnbGlmZXRpbWVfbWVhbicsICdlZGUxJykKYGBgCgpgYGB7cn0KcGxvdF9zdXBlcl9sZXR0ZXJzIDwtIHBsb3Rfc3VwZXIgKwogIGdlb21fdGV4dChkYXRhID0gc2xhMV9saWZldGltZV9zdGF0cywKICAgICAgICAgICAgYWVzKGxhYmVsID0gdHVrZXlfZ3JvdXApLCB5ID0gSW5mLCB2anVzdCA9IDEuMikKCnByaW50KHBsb3Rfc3VwZXJfbGV0dGVycykKbXlfZ2dzYXZlKCdmaWd1cmVzL2xpZmV0aW1lX3N1cGVyX2xldHRlcnMucGRmJykKYGBgCgojIyBIeXBvdGhlc2lzIHRlc3RzIHsudGFic2V0fQoKIyMjIEFzc3VtcHRpb25zCgpBTk9WQSBhbmQgc2ltaWxhciBwYXJhbWV0cmljIHRlc3RzIAphc3N1bWUgdGhhdCB0aGUgZXJyb3JzIGFyZSBub3JtYWxseSBkaXN0cmlidXRlZCwKd2l0aCBob21vZ2VuZW91cyB2YXJpYW5jZXMsCmFuZCB0aGF0IHRoZSBzYW1wbGVzIGFyZSBpbmRlcGVuZGVudC4KV2Ugd2lsbCB0ZXN0IHRoZSBudWxsIGh5cG90aGVzaXMgdGhhdCBtZWFuIFNsYTEgbGlmZXRpbWUgaXMgdGhlIHNhbWUKYWNyb3NzIGRpZmZlcmVudCBFZGUxIHN0cmFpbnMuCgpXZSB3aWxsIHVzZSByZXBlYXQtbGV2ZWwgZGF0YSBmb3IgdGhlIHRlc3RzCnRvIGFjY291bnQgZm9yIGV4cGVyaW1lbnRhbCB2YXJpYWJpbGl0eS4KQWxzbywgZXZlbiBpZiB0aGUgcG9wdWxhdGlvbnMgYXJlIHNrZXdlZCAoYXMgaXQgc2VlbXMgZnJvbSB0aGUgcGxvdHMpLAp0aGUgc2FtcGxlIG1lYW5zIHNob3VsZCBzdGlsbCBiZSBub3JtYWxseSBkaXN0cmlidXRlZAooYWNjb3JkaW5nIHRvIHRoZSBDZW50cmFsIExpbWl0IFRoZW9yZW0pLiAKCiMjIyMgTm9ybWFsaXR5CgpGcm9tIHRoZSBwbG90cyBpdCBsb29rcyBsaWtlIHRoZSB1bmRlcmx5aW5nIGRhdGEKaXMgbm90IHBlcmZlY3RseSBub3JtYWwgd2l0aCBzb21lIHNrZXcuCldlIGNhbiBjaGVjayB0aGUgbm9ybWFsaXR5IG9mIHJlc2lkdWFscyB1c2VkIGluIHRoZSBtb2RlbCBsYXRlciwgYnV0IGl0IG1pZ2h0CnN0aWxsIGJlIGludGVyZXN0aW5nIHRvIGtub3cgaG93IG5vcm1hbCB0aGUgdW5kZXJseWluZyBkYXRhIGlzIG92ZXJhbGwuCgpJZiB3ZSBkbyBhIGZvcm1hbCB0ZXN0IChTaGFwaXJvLVdpbGtlcyk6CgpgYGB7cn0Kc2xhMV9saWZldGltZSAlPiUKICBncm91cF9ieShlZGUxKSAlPiUKICBzdW1tYXJpc2UobiA9IG4oKSwKICAgICAgICAgICAgcC52YWx1ZSA9IHRpZHkoc2hhcGlyby50ZXN0KGxpZmV0aW1lKSkkcC52YWx1ZSwgCiAgICAgICAgICAgIC5ncm91cHMgPSAnZHJvcCcpICU+JQogIGthYmxlKCkKYGBgCgpTaGFwaXJvLVdpbGtlcyByZWplY3RzIHRoZSBub3JtYWxpdHkgb2YgdGhlIGRhdGEgaW4gZWFjaCBncm91cC4KVGhhdCBpcyBhYm91dCBleHBlY3RlZCB3aXRoIGEgbGFyZ2Ugc2FtcGxlIHNpemUsIGJ1dCBpdApwcm9iYWJseSBhbHNvIHJlZmxlY3RzIGFuIGFjdHVhbCBza2V3IGluIGxpZmV0aW1lcy4KClEtUSBwbG90czoKCmBgYHtyIGZpZy5oZWlnaHQ9NiwgZmlnLndpZHRoPTh9CnNsYTFfbGlmZXRpbWUgJT4lCiAgZ2dwbG90KGFlcyhzYW1wbGUgPSBsaWZldGltZSkpKwogIGZhY2V0X3dyYXAoJ2VkZTEnLCBzY2FsZXMgPSAnZnJlZScpKyAgICAgICAgICAKICBzdGF0X3FxKHNoYXBlID0gMSkrCiAgc3RhdF9xcV9saW5lKCkKYGBgCgpBbGwgZGF0YXNldHMgZG8gaW5kZWVkIGxvb2sgaGVhdnktdGFpbGVkLiBIaXN0b2dyYW1zOgoKYGBge3IgZmlnLmhlaWdodD02LCBmaWcud2lkdGg9OH0Kc2xhMV9saWZldGltZSAlPiUKICBnZ3Bsb3QoYWVzKHggPSBsaWZldGltZSkpKwogIGZhY2V0X3dyYXAoJ2VkZTEnLCBzY2FsZXMgPSAnZnJlZScpKyAgICAgICAgICAKICBnZW9tX2hpc3RvZ3JhbSgpCmBgYAoKIyMjIyBIb21vc2NlZGFzdGljaXR5Cgo0IHBvaW50cyBwZXIgZ3JvdXAgaXMgcHJvYmFibHkgZW5vdWdoIHRvIGFzc2Vzcwp3aGV0aGVyIHRoZSB2YXJpYW5jZSBpcyBzaW1pbGFyIGluIHRoZSByZXBlYXQtbGV2ZWwgZGF0YS4KTGV2ZW5lJ3MgdGVzdDoKCmBgYHtyfQpzbGExX2xpZmV0aW1lX3N0YXRzICU+JQogIGxldmVuZV90ZXN0KGxpZmV0aW1lX21lYW4gfiBlZGUxKSAlPiUKICBrYWJsZSgpCmBgYAoKTGV2ZW5lJ3MgY2Fubm90IHJlamVjdCB0aGUgbnVsbCBoZXJlICh2YXJpYW5jZSBkb2VzIG5vdCBkaWZmZXIgYmV0d2VlbiBncm91cHMpLgoKIyMjIE9uZS13YXkgQU5PVkEKCmBgYHtyfQphbm92YSA8LSBhb3YobGlmZXRpbWVfbWVhbiB+IGVkZTEsIGRhdGEgPSBzbGExX2xpZmV0aW1lX3N0YXRzKQp0aWR5KGFub3ZhKSAlPiUga2FibGUoKQpgYGAKCiMjIyMgRGlhZ25vc3RpYyBwbG90cwoKYGBge3J9CnBsb3QoYW5vdmEpCmBgYAoKQWdhaW4sIHRoZSB2YXJpYW5jZSBsb29rcyBob21vZ2VuZW91cyBlbm91Z2guIApUaGVyZSBpcyBhIGRlZmluaXRlIGRlcGFydHVyZSBmcm9tIG5vcm1hbGl0eSBhcyB3ZWxsLCBhbHRob3VnaApJIGFtIG5vdCBzdXJlIGhvdyBjb25jZXJuaW5nIGl0IHJlYWxseSBpcy4KCiMjIyBQb3N0LWhvYyB0ZXN0IChUdWtleSkKCmBgYHtyfQp0dWtleSAlPiUKICBrYWJsZSgpCmBgYAoKQWxsIG11dGFudHMgYXJlIHNpZ25pZmljYW50bHkgZGlmZmVyZW50IHRoYW4gd2lsZC10eXBlLApidXQgbm90IG5lY2Vzc2FyaWx5IGJldHdlZW4gdGhlbXNlbHZlcy4gCk1vc3Qgbm90YWJseSwgd2UgZG8gbm90IGhhdmUgZW5vdWdoIHBvd2VyIHRvIHNheQppZiDiiIZDQyBpcyBkaWZmZXJlbnQgZnJvbSDiiIZQUSwg4oiGUFFDQyBvciBlZGUx4oiGLgpBdCB0aGUgc2FtZSB0aW1lLCDiiIZQUSBkaWZmZXJlbmNlIGZyb20g4oiGUFFDQyBhbmQgZWRlMeKIhiByZWFjaGVzCnRoZSBzaWduaWZpY2FuY2UgdGhyZXNob2xkLgoKIyMgT3ZlcmFsbCBzdW1tYXJ5CgpTdW1tYXJ5IHN0YXRpc3RpY3MgZm9yIGFsbCBleHBlcmltZW50cywgZGVyaXZlZCBmcm9tICptZWFuIHZhbHVlcyoKb2YgTiBpbmRlcGVuZGVudCByZXBlYXRzLgoKIyMjIEZpbmFsIGVzdGltYXRlcwoKRmluYWwgZXN0aW1hdGVzIHdpdGggbG93ZXIgLyB1cHBlciA5NSUgY29uZmlkZW5jZSBpbnRlcnZhbHMgYW5kIGEgY29tcGFyaXNvbgp0byB3aWxkIHR5cGUgKGluICUpLiBgaGFsZl9jaWAgaXMganVzdCB0aGUgZXJyb3IgZm9yIHdyaXRpbmcgQ0kgcmFuZ2VzCmluIHRoZSBmb3JtYXQgbWVhbiArLy0gZXJyb3IuCgpgYGB7cn0Kd3RfbWVhbiA8LSBzbGExX2xpZmV0aW1lX3N0YXRzICU+JQogIGZpbHRlcihlZGUxID09ICd3dCcpICU+JQogIHB1bGwobGlmZXRpbWVfbWVhbikgJT4lCiAgbWVhbigpCgpsaWZldGltZV9jaSA8LSBzbGExX2xpZmV0aW1lX3N0YXRzICU+JQogIGdyb3VwX2J5KGVkZTEpJT4lCiAgc3VtbWFyaXNlKG1lYW5fY2xfbm9ybWFsKGxpZmV0aW1lX21lYW4pKSAlPiUKICByZW5hbWUobWVhbiA9IHksIGxvd2VyID0geW1pbiwgdXBwZXIgPSB5bWF4KSAlPiUKICBtdXRhdGUocHJvY193dCA9IHJvdW5kKDEwMCAqIG1lYW4gLyB3dF9tZWFuKSwKICAgICAgICAgaGFsZl9jaSA9ICh1cHBlciAtIGxvd2VyKSAvIDIpIAoKa2FibGUobGlmZXRpbWVfY2ksIGRpZ2l0cyA9IDMpCmBgYAoKIyMjIENvbmNsdXNpb25zCgoxLiBBbGwgbXV0YXRpb25zIGNhdXNlIGEgc2lnbmlmaWNhbnQgcmVkdWN0aW9uIGluIFNsYTEgcGF0Y2ggbGlmZXRpbWUgZnJvbSB3aWxkIHR5cGUKMi4gRWRlMeKIhlBRQ0MgaXMgaW5kaXN0aW5ndWlzaGFibGUgZnJvbSBmdWxsIEVkZTEgZGVsZXRpb24sIAogIGNhdXNlcyB+MzAlIHJlZHVjdGlvbiBpbiBsaWZldGltZQozLiBJbmRpdmlkdWFsIFBRIC8gQ0MgZGVsZXRpb25zIGhhdmUgaW50ZXJtZWRpYXRlIGRlZmVjdHMKCiMjIyBNb3JlIHN0YXRpc3RpY3MKCmBgYHtyfQpzbGExX2xpZmV0aW1lX3N0YXRzICU+JQogIGdyb3VwX2J5KGVkZTEpJT4lCiAgc3VtbWFyaXNlKE4gPSBuKCksCiAgICAgICAgICAgIGFjcm9zcyhsaWZldGltZV9tZWFuLAogICAgICAgICAgICAgICAgICAgbGlzdChtZWFuID0gbWVhbiwgc2QgPSBzZCwKICAgICAgICAgICAgICAgICAgICAgICAgc2UgPSB+IHNkKC54KSAvIHNxcnQobigpKSwKICAgICAgICAgICAgICAgICAgICAgICAgbWVkaWFuID0gbWVkaWFuLCBtYWQgPSBtYWQpLAogICAgICAgICAgICAgICAgICAgICAgIC5uYW1lcyA9ICd7LmZufScpLAogICAgICAgICAgICAuZ3JvdXBzID0gJ2Ryb3AnKSAlPiUKICBrYWJsZShkaWdpdHMgPSAzKQpgYGAKCiMjIyBNb3JlIHN0YXRpc3RpY3MgKG9ic2VydmF0aW9uLWxldmVsKQoKSXQgbWlnaHQgYmUgdXNlZnVsIHRvIGFsc28gbG9vayBhdCBvYnNlcnZhdGlvbi1sZXZlbCBzdW1tYXJ5LgpUaGUgZXhwZXJpbWVudGFsIG1lYW5zIGNhbiBiZSB1c2VkIHRvIGRldGVybWluZQp0cnVlIHBvcHVsYXRpb24gbWVhbiAoYmVjYXVzZSBvZiB0aGUgQ0xUKSwKYnV0IGlmIHRoZSBwb3B1bGF0aW9uIGlzICByZWFsbHkgc2tld2VkLAptZWRpYW4gYW5kIHF1YXJ0aWxlcyBhcmUgcGFydGljdWxhcmx5IHVzZWZ1bCBpbmZvcm1hdGlvbgp3aGljaCBjYW5ub3QgYmUgYWNjdXJhdGVseSBhc3Nlc3NlZCBmcm9tIG9ubHkgNCBwb2ludHMuCgpgYGB7cn0Kc2xhMV9saWZldGltZSAlPiUKICBncm91cF9ieShlZGUxKSU+JQogIHN1bW1hcmlzZShuID0gbigpLAogICAgICAgICAgICBhY3Jvc3MobGlmZXRpbWUsCiAgICAgICAgICAgICAgICAgICBsaXN0KG1lYW4gPSBtZWFuLCBzZCA9IHNkLAogICAgICAgICAgICAgICAgICAgICAgICBzZSA9IH4gc2QoLngpIC8gc3FydChuKCkpLAogICAgICAgICAgICAgICAgICAgICAgICBtZWRpYW4gPSBtZWRpYW4sIG1hZCA9IG1hZAogICAgICAgICAgICAgICAgICAgICAgICAjLCBxdWFudCA9IH5xdWFudGlsZSgueCwgYygwLjI1LCAwLjUsIDAuNzUpKQogICAgICAgICAgICAgICAgICAgICAgICApLAogICAgICAgICAgICAgICAgICAgICAgIC5uYW1lcyA9ICd7LmZufScpLAogICAgICAgICAgICBwaXZvdF93aWRlcihlbmZyYW1lKHF1YW50aWxlKGxpZmV0aW1lLCBjKDAuMjUsIDAuNzUpKSkpLAogICAgICAgICAgICAuZ3JvdXBzID0gJ2Ryb3AnKSAlPiUKICAKICBrYWJsZShkaWdpdHMgPSAzKQpgYGAKCiMjIFNvdXJjZSBkYXRhCgojIyMgLmNzdgoKYGBge3IgZWNobz1GQUxTRX0KeGZ1bjo6ZW1iZWRfZmlsZSgnZGF0YS9zbGExX2xpZmV0aW1lLmNzdicpCmBgYAoKIyMjIC5SRGF0YQoKYGBge3IgZWNobz1GQUxTRX0KeGZ1bjo6ZW1iZWRfZmlsZSgnZGF0YS9zbGExX2xpZmV0aW1lLlJEYXRhJykKYGBgCgojIyBTZXNzaW9uIGluZm8KCmBgYHtyIHNlc3Npb24sIG1lc3NhZ2U9VFJVRX0Kc2Vzc2lvbkluZm8oKQpgYGA=
