# Supplementary material for: Condensation of Ede1 promotes the initiation of endocytosis
Source: eLife. 2022 Apr 12;11:e72865. doi: 10.7554/eLife.72865 (PMC9064294; doi:10.7554/eLife.72865)
Supplement: Figure 8—source data 1. [file elife-72865-fig8-data1.zip › fig8_data1.html]

Sla1 patch density in Ede1 internal replacement mutants


Code 

- Download Rmd

# Sla1 patch density in Ede1 internal replacement mutants

#### Last compiled on March 25, 2022

# 

## Experiment

The aim of the experiment is to determine the density of Sla1-EGFP patches in yeast mutants where the central region of Ede1 protein (amino acids 366-900, PQ-rich and coiled-coil domains) was replaced by a heterologous protein domain, one of:

- yeast Snf5 prion-like IDR
- yeast Sup35 prion-like IDR
- mCherry (monomeric fluorescent protein)
- dTomato (dimeric fluorescent protein)
- kinesin-1 coiled-coil from *Drosophila melanogaster* (dimeric)
- kinesin-5 coiled-coil from *Homo sapiens* (tetrameric)

### Imaging

I acquired all data on the Olympus IX81 equipped with a 100x/1.49 objective, using the X-Cite 120PC lamp at 50% intensity and 400 ms exposure for illumination. Light was filtered through a U-MGFPHQ filter cube. I acquired stacks of 26 planes with a step size of 0.2 microns.

### Image processing

Individual non-budding cells were cropped from fields of view. Patch numbers were extracted using Python function `count_patches` from my personal package `mkimage` containing a set of wrappers for `scikit-image` functions. Briefly, the images were median-filtered with a 5 px disk brush, and the filtered images were subtracted from the originals to subtract local background. The background-subtracted images were thresholded using the Yen method. The thresholded images were eroded using the number of non-zero neighbouring pixels in 3D as the erosion criterion. The spots were counted using skimage.measure.label() function with 2-connectivity.

Cross-section area was obtained by median-filtering of the stack with 10px disk brush, calculating the maximum projection image, thresholding using Otsu’s algorithm, and using skimage.measure.regionprops() to measure area. Note that these are pixel counts of *cross-section* area. To determine the total surface area, I assumed that an unbudded cell is spherical (suface area is four times the cross-section area).

This call to `site_counter` was used to process all datasets:

```
process_folder(path, median_radius = 5, erosion_n = 1, con = 2,
                   method = Yen, mask = False, loop = False, save_images = True)
```

Another notebook was used to gather all output into tidy data frames available here, with no further modifications.

### Strain list

| strain | ede1 |
| --- | --- |
| MKY0140 | EDE1 |
| MKY3782 | ∆PQCC |
| MKY0654 | ede1∆ |
| MKY4617 | mCherry |
| MKY4576 | dTom |
| MKY4578 | Khc |
| MKY4580 | Eg5 |
| MKY4291 | Snf5 |
| MKY4282 | Sup35 |

## Per-dataset summary

### Patch number and area

| ede1 | dataset | n | patches\_mean | patches\_sd | patches\_se | area\_mean | area\_sd | area\_se |
| --- | --- | --- | --- | --- | --- | --- | --- | --- |
| EDE1 | 1 | 63 | 27.73016 | 6.715909 | 0.8461250 | 51.94079 | 10.645161 | 1.3411642 |
| EDE1 | 2 | 58 | 29.12069 | 5.846209 | 0.7676449 | 56.44771 | 11.552946 | 1.5169761 |
| EDE1 | 3 | 64 | 29.32812 | 6.519944 | 0.8149930 | 49.56184 | 7.991786 | 0.9989733 |
| ∆PQCC | 1 | 73 | 16.41096 | 4.954896 | 0.5799267 | 52.33093 | 11.200530 | 1.3109229 |
| ∆PQCC | 2 | 74 | 18.44595 | 5.698214 | 0.6624039 | 54.32342 | 10.600985 | 1.2323395 |
| ∆PQCC | 3 | 58 | 18.29310 | 6.833890 | 0.8973338 | 54.40918 | 11.287895 | 1.4821732 |
| ede1∆ | 1 | 61 | 14.98361 | 4.801013 | 0.6147067 | 52.08469 | 7.516644 | 0.9624076 |
| ede1∆ | 2 | 65 | 15.98462 | 5.704966 | 0.7076139 | 56.48160 | 10.966704 | 1.3602522 |
| ede1∆ | 3 | 60 | 14.61667 | 5.249993 | 0.6777712 | 48.98833 | 8.084606 | 1.0437182 |
| mCherry | 4 | 54 | 17.70370 | 5.638913 | 0.7673589 | 59.99512 | 15.108871 | 2.0560569 |
| mCherry | 5 | 70 | 18.48571 | 4.760169 | 0.5689491 | 50.13624 | 7.121412 | 0.8511715 |
| mCherry | 6 | 78 | 14.21795 | 4.874123 | 0.5518858 | 49.94668 | 9.651742 | 1.0928446 |
| dTom | 4 | 68 | 15.82353 | 5.268723 | 0.6389265 | 48.63283 | 7.470632 | 0.9059472 |
| dTom | 5 | 59 | 15.71186 | 5.378921 | 0.7002758 | 53.89653 | 9.640562 | 1.2550942 |
| dTom | 6 | 42 | 18.92857 | 5.654236 | 0.8724675 | 55.65860 | 10.453794 | 1.6130555 |
| Khc | 4 | 61 | 13.62295 | 5.612973 | 0.7186675 | 47.46450 | 6.633740 | 0.8493634 |
| Khc | 5 | 64 | 14.31250 | 4.189462 | 0.5236827 | 49.50749 | 8.223165 | 1.0278956 |
| Khc | 6 | 54 | 16.62963 | 5.003004 | 0.6808226 | 53.91222 | 8.693893 | 1.1830890 |
| Eg5 | 4 | 60 | 18.81667 | 5.776902 | 0.7457949 | 50.86488 | 6.825151 | 0.8811232 |
| Eg5 | 5 | 78 | 16.06410 | 5.112602 | 0.5788881 | 50.41711 | 10.283088 | 1.1643305 |
| Eg5 | 6 | 60 | 16.26667 | 5.868233 | 0.7575856 | 51.62094 | 11.500195 | 1.4846688 |
| Snf5 | 1 | 90 | 19.34444 | 6.182907 | 0.6517356 | 45.21896 | 7.520545 | 0.7927350 |
| Snf5 | 2 | 79 | 20.75949 | 5.137133 | 0.5779726 | 56.24489 | 10.959846 | 1.2330790 |
| Snf5 | 3 | 62 | 18.59677 | 8.177257 | 1.0385127 | 48.89502 | 11.491233 | 1.4593880 |
| Sup35 | 1 | 77 | 22.02597 | 5.155411 | 0.5875136 | 51.53718 | 8.410510 | 0.9584666 |
| Sup35 | 2 | 61 | 21.21311 | 7.071338 | 0.9053921 | 55.34524 | 9.881855 | 1.2652419 |
| Sup35 | 3 | 54 | 22.11111 | 5.967417 | 0.8120626 | 52.71252 | 10.581480 | 1.4399571 |

### Sla1 density

We can combine the patch number and area into \(density = \frac{patches}{area}\), calculated individually for each cell. We can summarise the data for each Ede1 mutant in each dataset:

| ede1 | dataset | n | density\_mean | density\_sd | density\_se | density\_median | density\_mad |
| --- | --- | --- | --- | --- | --- | --- | --- |
| EDE1 | 1 | 63 | 0.5481701 | 0.1446301 | 0.0182217 | 0.5645806 | 0.1440384 |
| EDE1 | 2 | 58 | 0.5277718 | 0.1198626 | 0.0157387 | 0.5120066 | 0.1172980 |
| EDE1 | 3 | 64 | 0.5948730 | 0.1110449 | 0.0138806 | 0.5869729 | 0.1016109 |
| ∆PQCC | 1 | 73 | 0.3178281 | 0.0936706 | 0.0109633 | 0.3144940 | 0.0989302 |
| ∆PQCC | 2 | 74 | 0.3463387 | 0.1057420 | 0.0122923 | 0.3237286 | 0.0907352 |
| ∆PQCC | 3 | 58 | 0.3343710 | 0.1086607 | 0.0142679 | 0.3384717 | 0.0852853 |
| ede1∆ | 1 | 61 | 0.2905893 | 0.0926398 | 0.0118613 | 0.2893412 | 0.0784925 |
| ede1∆ | 2 | 65 | 0.2812039 | 0.0815060 | 0.0101096 | 0.2864278 | 0.0766339 |
| ede1∆ | 3 | 60 | 0.2988538 | 0.1021170 | 0.0131832 | 0.3085457 | 0.1075222 |
| mCherry | 4 | 54 | 0.2960346 | 0.0649451 | 0.0088379 | 0.2913582 | 0.0658805 |
| mCherry | 5 | 70 | 0.3720760 | 0.0922860 | 0.0110303 | 0.3616950 | 0.0877807 |
| mCherry | 6 | 78 | 0.2871355 | 0.0912629 | 0.0103335 | 0.3002458 | 0.0946702 |
| dTom | 4 | 68 | 0.3240302 | 0.0977476 | 0.0118536 | 0.3172533 | 0.0954928 |
| dTom | 5 | 59 | 0.2908285 | 0.0862263 | 0.0112257 | 0.2827884 | 0.0858295 |
| dTom | 6 | 42 | 0.3464929 | 0.1032743 | 0.0159356 | 0.3458182 | 0.0946929 |
| Khc | 4 | 61 | 0.2850212 | 0.1032656 | 0.0132218 | 0.2863498 | 0.1103514 |
| Khc | 5 | 64 | 0.2929173 | 0.0839598 | 0.0104950 | 0.2984537 | 0.0814191 |
| Khc | 6 | 54 | 0.3084453 | 0.0835851 | 0.0113745 | 0.2990882 | 0.0817657 |
| Eg5 | 4 | 60 | 0.3698412 | 0.1012370 | 0.0130696 | 0.3727275 | 0.1171072 |
| Eg5 | 5 | 78 | 0.3236706 | 0.0976318 | 0.0110546 | 0.3266622 | 0.0931995 |
| Eg5 | 6 | 60 | 0.3182508 | 0.1076020 | 0.0138914 | 0.3163651 | 0.1029646 |
| Snf5 | 1 | 90 | 0.4276246 | 0.1221145 | 0.0128720 | 0.4228668 | 0.1211030 |
| Snf5 | 2 | 79 | 0.3760389 | 0.0943727 | 0.0106178 | 0.3684693 | 0.1011870 |
| Snf5 | 3 | 62 | 0.3725138 | 0.1277070 | 0.0162188 | 0.3669131 | 0.1374652 |
| Sup35 | 1 | 77 | 0.4362761 | 0.1133273 | 0.0129148 | 0.4255176 | 0.1124559 |
| Sup35 | 2 | 61 | 0.3870802 | 0.1225512 | 0.0156911 | 0.3899448 | 0.1170396 |
| Sup35 | 3 | 54 | 0.4281938 | 0.1241646 | 0.0168967 | 0.4192432 | 0.1205993 |

## Plots

### SuperPlots

I have chosen to show this data using the SuperPlot style. Each point shows density of Sla1-EGFP patches in an individual cell.

Big colour points show mean measurements from three independent repeats.

Range is mean +/- SD, calculated based on the three independent repeat means.

#### Beeswarm

#### Violin

### With significance

There are 9 levels of Ede1, resulting in 36 possible pairwise comparisons. This is too unwieldy for any plot, at least a plot intended to also show the data.

As such, I propose to not even attempt this kind of illustration. An alternative is a compact letter display. In this view, groups sharing at least one letter are not significantly different at a chosen \(\alpha\) (here, 5%).

Pros:

- less cluttered and simpler to read
- focus away from p-values; provide a binary decision on the null hypothesis

Cons:

- cannot distinguish different confidence levels
- can get complicated just as well
- focuses on non-findings rather than findings.

As this is also complicated, I propose a final solution: indicate that all groups are significantly different from wild-type in the text, and mark those with a significant improvement from ede1∆PQCC with a star, all while providing a table of p-values for each pairwise comparison in the supplement.

### Pooled box plot

This might be useful in situations where this much data is too much data.

## Modeling

We want to consider:

1. What are the Sla1 densities in different Ede1 backgrounds?
2. Are the differences between strains statistically significant?

The design is not ideal here:

- we want to compare all mutants against each other
- we want to block for nuisance factors, which might come from co-culturing the cells or things related to the microscope such as lamp power hence the independently repeated experiments
- half the experiment was added *after* the reviewers request, so the design is disconnected. Datasets 1-3 have both controls and IDRs, datasets 4-6 have the globular / coiled-coil replacements. Finally, the mCherry strain was added after all of the others on separate dates.

Bad design cannot be solved in statistical analysis, but on the bright side, the data is nice and there is no reason for a systematic error to be associated with any particular dataset. Since we are measuring an absolute property (number of sites), and not something directly proportional to exposure settings like intensity, the influence of lamp power etc. should be limited

If the experiment was not disconnected, we could try to see if the effects of date / dataset can be modeled.

Because it *is* disconnected, it’s a bit more tricky. For example we cannot really get useful information from trying to model `date` as a fixed effect after `ede1`. What we could do for starters is to make a model which disregards the dataset information, and look if the residuals are clustered by date or dataset, informing us about whether this actually matters.

```
pooled_lm <- lm(density ~ ede1, data = sla1_density)
plot(as.factor(sla1_density$date), resid(pooled_lm),
     xlab = '', ylab = 'Residuals', las = 2,
     main = 'Univariate model residuals by date')
```

These seem to be very well distributed around zero. The exception is the single mCherry acquisition on 10/02/2022, which, to be honest, does seem like an outlier.

So what to do? The decision seems fairly arbitrary. I do not necessarily think that the univariate model on all cells would be *wrong*, but it does seem like a controversial issue. A model of replicate means (*means-pooled analysis*) will be conservative and lose power, but it seems appropriate for once, considering the segregated design.

### Means-pooled linear model ANOVA

We will test the null hypothesis that mean Sla1 density is the same across different Ede1 strains. We will use repeat-level data for the tests to account for experimental variability.

We can use ANOVA and a post-hoc test to find out how likely this data is to occur under the null hypothesis.

More importantly, we will try to find the effect sizes of different contrasts.

We start by making a model of `density_mean ~ ede1`:

```
## 
## Call:
## lm(formula = density_mean ~ ede1, data = sla1_density_stats)
## 
## Residuals:
##       Min        1Q    Median        3Q       Max 
## -0.031280 -0.017512 -0.002544  0.013238  0.053661 
## 
## Coefficients:
##             Estimate Std. Error t value Pr(>|t|)    
## (Intercept)  0.55694    0.01613  34.531  < 2e-16 ***
## ede1∆PQCC   -0.22409    0.02281  -9.825 1.17e-08 ***
## ede1ede1∆   -0.26672    0.02281 -11.694 7.64e-10 ***
## ede1mCherry -0.23852    0.02281 -10.457 4.47e-09 ***
## ede1dTom    -0.23649    0.02281 -10.368 5.11e-09 ***
## ede1Khc     -0.26148    0.02281 -11.464 1.05e-09 ***
## ede1Eg5     -0.21968    0.02281  -9.631 1.59e-08 ***
## ede1Snf5    -0.16488    0.02281  -7.229 1.01e-06 ***
## ede1Sup35   -0.13975    0.02281  -6.127 8.70e-06 ***
## ---
## Signif. codes:  0 '***' 0.001 '**' 0.01 '*' 0.05 '.' 0.1 ' ' 1
## 
## Residual standard error: 0.02794 on 18 degrees of freedom
## Multiple R-squared:  0.9236, Adjusted R-squared:  0.8897 
## F-statistic: 27.21 on 8 and 18 DF,  p-value: 1.573e-08
```

```
## Analysis of Variance Table
## 
## Response: density_mean
##           Df   Sum Sq   Mean Sq F value    Pr(>F)    
## ede1       8 0.169852 0.0212316  27.207 1.573e-08 ***
## Residuals 18 0.014047 0.0007804                      
## ---
## Signif. codes:  0 '***' 0.001 '**' 0.01 '*' 0.05 '.' 0.1 ' ' 1
```

One-way ANOVA on means-pooled data rejects the null with \(p = 1.5 \times 10^{-8}\).

#### Diagnostic plots

This is one problem with the small N of replicate means: there are definitely differences in variance for different levels, but we cannot tell whether they reflect a difference in populations or just noise. But it’s probably the lattter; if we go back to the cell-level observations modeled by `pooled_lm` we can see that the residuals are fine there, too:

### Estimated group means

The following are the model estimates of mean densities associates with each Ede1 level:

```
##  ede1    emmean     SE df lower.CL upper.CL
##  EDE1     0.557 0.0161 18    0.523    0.591
##  ∆PQCC    0.333 0.0161 18    0.299    0.367
##  ede1∆    0.290 0.0161 18    0.256    0.324
##  mCherry  0.318 0.0161 18    0.285    0.352
##  dTom     0.320 0.0161 18    0.287    0.354
##  Khc      0.295 0.0161 18    0.262    0.329
##  Eg5      0.337 0.0161 18    0.303    0.371
##  Snf5     0.392 0.0161 18    0.358    0.426
##  Sup35    0.417 0.0161 18    0.383    0.451
## 
## Confidence level used: 0.95
```

### Post-hoc test (Tukey)

We are not necessarily interested in every possible comparison, but we do want to obtain all the p-values at least against ∆PQCC (is there a rescue?) and wild-type (is it complete?). Tukey’s test for contrasts of all 9 estimates:

```
##  contrast        estimate     SE df t.ratio p.value
##  EDE1 - ∆PQCC     0.22409 0.0228 18   9.825  <.0001
##  EDE1 - ede1∆     0.26672 0.0228 18  11.694  <.0001
##  EDE1 - mCherry   0.23852 0.0228 18  10.457  <.0001
##  EDE1 - dTom      0.23649 0.0228 18  10.368  <.0001
##  EDE1 - Khc       0.26148 0.0228 18  11.464  <.0001
##  EDE1 - Eg5       0.21968 0.0228 18   9.631  <.0001
##  EDE1 - Snf5      0.16488 0.0228 18   7.229  <.0001
##  EDE1 - Sup35     0.13975 0.0228 18   6.127  0.0002
##  ∆PQCC - ede1∆    0.04263 0.0228 18   1.869  0.6407
##  ∆PQCC - mCherry  0.01443 0.0228 18   0.633  0.9991
##  ∆PQCC - dTom     0.01240 0.0228 18   0.543  0.9997
##  ∆PQCC - Khc      0.03738 0.0228 18   1.639  0.7727
##  ∆PQCC - Eg5     -0.00441 0.0228 18  -0.193  1.0000
##  ∆PQCC - Snf5    -0.05921 0.0228 18  -2.596  0.2537
##  ∆PQCC - Sup35   -0.08434 0.0228 18  -3.698  0.0341
##  ede1∆ - mCherry -0.02820 0.0228 18  -1.236  0.9371
##  ede1∆ - dTom    -0.03023 0.0228 18  -1.326  0.9105
##  ede1∆ - Khc     -0.00525 0.0228 18  -0.230  1.0000
##  ede1∆ - Eg5     -0.04704 0.0228 18  -2.062  0.5245
##  ede1∆ - Snf5    -0.10184 0.0228 18  -4.465  0.0071
##  ede1∆ - Sup35   -0.12697 0.0228 18  -5.567  0.0007
##  mCherry - dTom  -0.00204 0.0228 18  -0.089  1.0000
##  mCherry - Khc    0.02295 0.0228 18   1.006  0.9803
##  mCherry - Eg5   -0.01884 0.0228 18  -0.826  0.9943
##  mCherry - Snf5  -0.07364 0.0228 18  -3.229  0.0847
##  mCherry - Sup35 -0.09877 0.0228 18  -4.330  0.0094
##  dTom - Khc       0.02499 0.0228 18   1.096  0.9675
##  dTom - Eg5      -0.01680 0.0228 18  -0.737  0.9974
##  dTom - Snf5     -0.07161 0.0228 18  -3.139  0.1000
##  dTom - Sup35    -0.09673 0.0228 18  -4.241  0.0113
##  Khc - Eg5       -0.04179 0.0228 18  -1.832  0.6627
##  Khc - Snf5      -0.09660 0.0228 18  -4.235  0.0114
##  Khc - Sup35     -0.12172 0.0228 18  -5.337  0.0012
##  Eg5 - Snf5      -0.05480 0.0228 18  -2.403  0.3387
##  Eg5 - Sup35     -0.07993 0.0228 18  -3.504  0.0500
##  Snf5 - Sup35    -0.02512 0.0228 18  -1.102  0.9665
## 
## P value adjustment: tukey method for comparing a family of 9 estimates
```

This is a lot; perhaps we can put it in a graphical matrix.

#### p-value matrix

#### The bottom line

- every mutant is significantly different from wild-type at \(\alpha = 0.05\)
- only Sup35 replacement is a significant improvement over ∆PQCC

### Effect sizes

Effect sizes are perhaps more important than the p-values. Here I will calculate the classical Cohen’s d, defined as the mean differences over population standard deviation. Despite using means-pooled model to estimate contrast p-values and population means, I am confident that it is more appropriate to take the pooled observation-level SD for calculating the effect size.

Therefore \(\sigma\) will be a mean of SD for each Ede1 level, weighed by \(n-1\) (departing from `emmeans` default suggestion of residual SD). I am not quite sure what the appropriate degrees of freedom are, I used sample size minus Ede1 level count. In any case, it affects the 95% CI’s of effect size estimate, but not the estimate itself.

Below are effect sizes calculated for comparisons against wild-type and against ∆PQCC.

```
##  contrast       effect.size    SE df lower.CL upper.CL
##  ∆PQCC - EDE1         -2.11 0.217 18    -2.56   -1.651
##  ede1∆ - EDE1         -2.51 0.219 18    -2.97   -2.049
##  mCherry - EDE1       -2.24 0.218 18    -2.70   -1.786
##  dTom - EDE1          -2.22 0.218 18    -2.68   -1.767
##  Khc - EDE1           -2.46 0.219 18    -2.92   -2.000
##  Eg5 - EDE1           -2.07 0.217 18    -2.52   -1.609
##  Snf5 - EDE1          -1.55 0.216 18    -2.00   -1.097
##  Sup35 - EDE1         -1.31 0.216 18    -1.77   -0.861
## 
## sigma used for effect sizes: 0.1063 
## Confidence level used: 0.95
```

```
##  contrast        effect.size    SE df lower.CL upper.CL
##  EDE1 - ∆PQCC         2.1076 0.217 18    1.651   2.5645
##  ede1∆ - ∆PQCC       -0.4009 0.215 18   -0.852   0.0500
##  mCherry - ∆PQCC     -0.1357 0.215 18   -0.586   0.3150
##  dTom - ∆PQCC        -0.1166 0.215 18   -0.567   0.3341
##  Khc - ∆PQCC         -0.3516 0.215 18   -0.802   0.0993
##  Eg5 - ∆PQCC          0.0415 0.215 18   -0.409   0.4921
##  Snf5 - ∆PQCC         0.5569 0.215 18    0.106   1.0080
##  Sup35 - ∆PQCC        0.7932 0.215 18    0.342   1.2448
## 
## sigma used for effect sizes: 0.1063 
## Confidence level used: 0.95
```

### Addendum: mixed model analysis

The p-values for different contrasts above are a result of a quite conservative model, which itself is a result of suboptimal design. In a pooled observation model, for example, we would find a couple more significant effects due to larger sample size:

```
##            EDE1   ∆PQCC   ede1∆ mCherry    dTom     Khc     Eg5    Snf5   Sup35
## EDE1    [0.558]  <.0001  <.0001  <.0001  <.0001  <.0001  <.0001  <.0001  <.0001
## ∆PQCC           [0.333]  0.0024  0.9274  0.9199  0.0149  1.0000  <.0001  <.0001
## ede1∆                   [0.290]  0.1551  0.2413  1.0000  0.0008  <.0001  <.0001
## mCherry                         [0.319]  1.0000  0.4044  0.8021  <.0001  <.0001
## dTom                                    [0.318]  0.5249  0.7960  <.0001  <.0001
## Khc                                             [0.295]  0.0057  <.0001  <.0001
## Eg5                                                     [0.336]  <.0001  <.0001
## Snf5                                                            [0.395]  0.3849
## Sup35                                                                   [0.418]
## 
## Row and column labels: ede1
## Upper triangle: P values   adjust = "tukey"
## Diagonal: [Estimates] (emmean)
```

Here we get low p-values for the differences between Snf5 and everything else, as well as ∆PQCC and actually *less* dense Khc/ede1∆.

This model would generate group means and effect sizes pretty much the same as the means-pooled model, but drastically different p-values. This serves to underscore that p-values should be treated with healthy skepticism, and play a minor role in the interpretation of the results.

Some would say that this model is an example of *pseudoreplication*, but this is a bit of a grey area. Yeast cells from different datasets grow in the same medium, incubator and so on, but they are individual organisms. Even if a ‘date effect’ exists beyond a simple sampling variation, there is no reason to think that the effect is consistent across mutants: after all, each mutant grows in a separate tube. If we try to look at an interaction plot, we cannot see a consistent date effect:

Of course this is undermined by the disconnect in date / mutant combinations.

Can we generate a linear mixed model for this, using `date` as a random effect? Because there does not seem to be any consistent effect of date in itself, let’s add an interaction term.

```
date_lmm1 <- lmer(density ~ ede1 + (1|date), data = sla1_density)
date_lmm2 <- lmer(density ~ ede1 + (1|ede1:date), data = sla1_density)
date_lmm3 <- lmer(density ~ ede1 + (1|date) + (1|ede1:date), data = sla1_density)
```

```
## Data: sla1_density
## Models:
## date_lmm1: density ~ ede1 + (1 | date)
## date_lmm2: density ~ ede1 + (1 | ede1:date)
## date_lmm3: density ~ ede1 + (1 | date) + (1 | ede1:date)
##           npar     AIC     BIC logLik deviance  Chisq Df Pr(>Chisq)
## date_lmm1   11 -2879.1 -2818.9 1450.5  -2901.1                     
## date_lmm2   11 -2887.4 -2827.2 1454.7  -2909.4 8.2988  0           
## date_lmm3   12 -2885.4 -2819.8 1454.7  -2909.4 0.0017  1      0.967
```

Using the `anova` table for mixed models from `lmerTest` we actually find the interaction-only model `date_lmm2` has the lowest AIC.

Single-term deletions of effects from the main + interaction model `date_lmm3` suggest that the interaction model is significant and the main date effect is not.

```
## ANOVA-like table for random-effects: Single term deletions
## 
## Model:
## density ~ ede1 + (1 | date) + (1 | ede1:date)
##                 npar logLik     AIC     LRT Df Pr(>Chisq)    
## <none>            12 1424.9 -2825.8                          
## (1 | date)        11 1424.9 -2827.8  0.0007  1  0.9796497    
## (1 | ede1:date)   11 1417.8 -2813.6 14.1967  1  0.0001647 ***
## ---
## Signif. codes:  0 '***' 0.001 '**' 0.01 '*' 0.05 '.' 0.1 ' ' 1
```

What does this mean? I am getting out of my depth here, but I think that we simply cannot assign a meaningful change of intercept based on `date` because the effect is different for every Ede1 level (hence the interaction). Again, this makes sense because while theoretically there could be some issues affecting all strains on a given day (say, incubator failure), this just did not happen and so it’s not reflected in the data beyond a simple sampling error and that is going to be unique to each sample.

Note that this is very unlike the dataset in Figure 3, where any change in the microscope will affect intensity readings.

Let’s look at what the interaction-only model says about `ede1` effects on `density`. First, how do model residuals look? Pretty good, but it’s not like the pooled observation-only model had any problems.

Even if we accept the interaction-only model, the random factor does not explain much of the variance anyway:

```
## Linear mixed model fit by REML. t-tests use Satterthwaite's method [
## lmerModLmerTest]
## Formula: density ~ ede1 + (1 | ede1:date)
##    Data: sla1_density
## 
## REML criterion at convergence: -2849.8
## 
## Scaled residuals: 
##     Min      1Q  Median      3Q     Max 
## -3.6089 -0.6576 -0.0241  0.6473  3.8921 
## 
## Random effects:
##  Groups    Name        Variance  Std.Dev.
##  ede1:date (Intercept) 0.0006184 0.02487 
##  Residual              0.0108813 0.10431 
## Number of obs: 1747, groups:  ede1:date, 27
## 
## Fixed effects:
##             Estimate Std. Error       df t value Pr(>|t|)    
## (Intercept)  0.55716    0.01628 18.50247  34.224  < 2e-16 ***
## ede1∆PQCC   -0.22433    0.02291 18.12580  -9.793 1.15e-08 ***
## ede1ede1∆   -0.26700    0.02302 18.47895 -11.601 6.37e-10 ***
## ede1mCherry -0.23854    0.02294 18.20417 -10.401 4.33e-09 ***
## ede1dTom    -0.23743    0.02318 18.98038 -10.241 3.62e-09 ***
## ede1Khc     -0.26183    0.02307 18.64776 -11.350 8.20e-10 ***
## ede1Eg5     -0.22015    0.02295 18.26477  -9.592 1.48e-08 ***
## ede1Snf5    -0.16452    0.02279 17.73636  -7.220 1.12e-06 ***
## ede1Sup35   -0.13977    0.02300 18.39854  -6.078 8.74e-06 ***
## ---
## Signif. codes:  0 '***' 0.001 '**' 0.01 '*' 0.05 '.' 0.1 ' ' 1
## 
## Correlation of Fixed Effects:
##             (Intr) e1∆PQC ed1d1∆ ed1mCh ed1dTm ed1Khc ed1Eg5 ed1Sn5
## ede1∆PQCC   -0.711                                                 
## ede1ede1∆   -0.707  0.503                                          
## ede1mCherry -0.710  0.504  0.502                                   
## ede1dTom    -0.702  0.499  0.497  0.498                            
## ede1Khc     -0.706  0.502  0.499  0.501  0.496                     
## ede1Eg5     -0.709  0.504  0.502  0.503  0.498  0.501              
## ede1Snf5    -0.714  0.508  0.505  0.507  0.502  0.504  0.507       
## ede1Sup35   -0.708  0.503  0.501  0.503  0.497  0.500  0.502  0.506
```

We have 5% of variance explained by the `1|ede1:date` term in the model.

Let’s quickly generate group means (diagonal) and p-values for contrasts, with Tukey adjustment:

```
##            EDE1   ∆PQCC   ede1∆ mCherry    dTom     Khc     Eg5    Snf5   Sup35
## EDE1    [0.557]  <.0001  <.0001  <.0001  <.0001  <.0001  <.0001  <.0001  0.0003
## ∆PQCC           [0.333]  0.6443  0.9992  0.9996  0.7755  1.0000  0.2402  0.0347
## ede1∆                   [0.290]  0.9359  0.9265  1.0000  0.5368  0.0070  0.0008
## mCherry                         [0.319]  1.0000  0.9795  0.9952  0.0820  0.0099
## dTom                                    [0.320]  0.9748  0.9971  0.0935  0.0113
## Khc                                             [0.295]  0.6746  0.0112  0.0012
## Eg5                                                     [0.337]  0.3189  0.0500
## Snf5                                                            [0.393]  0.9687
## Sup35                                                                   [0.417]
## 
## Row and column labels: ede1
## Upper triangle: P values   adjust = "tukey"
## Diagonal: [Estimates] (emmean)
```

The values are strikingly similar to what we saw with the means-pooled model. Why? I suspect that these are actually equivalent because the mixed model we ended up with creates 27 unique groups with no shared information by `ede1` / `date` combination just as we have 27 `ede1` / `dataset` unique group means in the `replicate_lm` model.

## Conclusions

### Actual conclusions

1. All mutations cause a significant reduction in patch density from wild type
2. While Ede1∆PQCC is not *significantly* different from ede1∆, it has to be noted that the estimate is higher (~40% reduction instead of ~50%).
3. Snf5 and Sup35 substitutions have significantly higher Sla1 densities than ede1∆; Sup35 is also significantly different from ∆PQCC.
4. All the other mutants are similar across the board.
5. Stepping away from p-values: IDRs rescue Sla1 density only somewhat, the others not at all. That is the take-home message.

### Final estimates

Final estimates with lower / upper 95% confidence intervals, and a common-sense comparison to wild type (in %). `half_ci` is half of the confidence interval, for writing CI ranges in the format mean +/- error.

| ede1 | mean | lower | upper | proc\_wt | half\_ci |
| --- | --- | --- | --- | --- | --- |
| EDE1 | 0.557 | 0.471 | 0.642 | 100 | 0.085 |
| ∆PQCC | 0.333 | 0.297 | 0.368 | 60 | 0.036 |
| ede1∆ | 0.290 | 0.268 | 0.312 | 52 | 0.022 |
| mCherry | 0.318 | 0.202 | 0.434 | 57 | 0.116 |
| dTom | 0.320 | 0.251 | 0.390 | 58 | 0.070 |
| Khc | 0.295 | 0.266 | 0.325 | 53 | 0.030 |
| Eg5 | 0.337 | 0.267 | 0.408 | 61 | 0.070 |
| Snf5 | 0.392 | 0.315 | 0.469 | 70 | 0.077 |
| Sup35 | 0.417 | 0.352 | 0.483 | 75 | 0.066 |

## Source data

### .csv

Download idr\_replacement\_sla1.csv

### .RData

Download idr\_replacement\_sla1.RData

## Session info

```
## R version 4.1.0 (2021-05-18)
## Platform: x86_64-apple-darwin17.0 (64-bit)
## Running under: macOS Big Sur 10.16
## 
## Matrix products: default
## BLAS:   /Library/Frameworks/R.framework/Versions/4.1/Resources/lib/libRblas.dylib
## LAPACK: /Library/Frameworks/R.framework/Versions/4.1/Resources/lib/libRlapack.dylib
## 
## locale:
## [1] en_US.UTF-8/en_US.UTF-8/en_US.UTF-8/C/en_US.UTF-8/en_US.UTF-8
## 
## attached base packages:
## [1] stats     graphics  grDevices utils     datasets  methods   base     
## 
## other attached packages:
##  [1] emmeans_1.7.2      lmerTest_3.1-3     lme4_1.1-27.1      Matrix_1.3-3      
##  [5] multcompView_0.1-8 ggsignif_0.6.2     ggbeeswarm_0.6.0   knitr_1.36        
##  [9] broom_0.7.9        forcats_0.5.1      stringr_1.4.0      dplyr_1.0.7       
## [13] purrr_0.3.4        readr_2.0.1        tidyr_1.1.3        tibble_3.1.3      
## [17] ggplot2_3.3.5      tidyverse_1.3.1   
## 
## loaded via a namespace (and not attached):
##  [1] TH.data_1.1-0       minqa_1.2.4         colorspace_2.0-2   
##  [4] ellipsis_0.3.2      estimability_1.3    htmlTable_2.2.1    
##  [7] base64enc_0.1-3     fs_1.5.0            rstudioapi_0.13    
## [10] farver_2.1.0        fansi_0.5.0         mvtnorm_1.1-3      
## [13] lubridate_1.7.10    xml2_1.3.2          codetools_0.2-18   
## [16] splines_4.1.0       Formula_1.2-4       jsonlite_1.7.2     
## [19] nloptr_1.2.2.2      pbkrtest_0.5.1      cluster_2.1.2      
## [22] dbplyr_2.1.1        png_0.1-7           compiler_4.1.0     
## [25] httr_1.4.2          backports_1.2.1     assertthat_0.2.1   
## [28] cli_3.1.0           htmltools_0.5.1.1   tools_4.1.0        
## [31] gtable_0.3.0        glue_1.4.2          Rcpp_1.0.7         
## [34] cellranger_1.1.0    jquerylib_0.1.4     vctrs_0.3.8        
## [37] nlme_3.1-152        xfun_0.25           rvest_1.0.1        
## [40] mime_0.11           lifecycle_1.0.0     MASS_7.3-54        
## [43] zoo_1.8-9           scales_1.1.1        hms_1.1.0          
## [46] parallel_4.1.0      sandwich_3.0-1      RColorBrewer_1.1-2 
## [49] yaml_2.2.1          gridExtra_2.3       rpart_4.1-15       
## [52] latticeExtra_0.6-29 stringi_1.7.3       highr_0.9          
## [55] checkmate_2.0.0     boot_1.3-28         rlang_0.4.11       
## [58] pkgconfig_2.0.3     evaluate_0.14       lattice_0.20-44    
## [61] htmlwidgets_1.5.3   labeling_0.4.2      tidyselect_1.1.1   
## [64] magrittr_2.0.1      R6_2.5.1            generics_0.1.0     
## [67] Hmisc_4.5-0         multcomp_1.4-18     DBI_1.1.1          
## [70] pillar_1.6.2        haven_2.4.3         foreign_0.8-81     
## [73] withr_2.4.2         survival_3.2-11     nnet_7.3-16        
## [76] modelr_0.1.8        crayon_1.4.1        utf8_1.2.2         
## [79] tzdb_0.1.2          rmarkdown_2.11      jpeg_0.1-9         
## [82] grid_4.1.0          readxl_1.3.1        data.table_1.14.0  
## [85] reprex_2.0.1        digest_0.6.27       xtable_1.8-4       
## [88] numDeriv_2016.8-1.1 munsell_0.5.0       beeswarm_0.4.0     
## [91] vipor_0.4.5
```

## WIP

LS0tCnRpdGxlOiAiU2xhMSBwYXRjaCBkZW5zaXR5IGluIEVkZTEgaW50ZXJuYWwgcmVwbGFjZW1lbnQgbXV0YW50cyIKZGF0ZTogIkxhc3QgY29tcGlsZWQgb24gYHIgZm9ybWF0KFN5cy50aW1lKCksICclQiAlZCwgJVknKWAiCm91dHB1dDoKICBodG1sX2RvY3VtZW50OgogICAgY29kZV9kb3dubG9hZDogdHJ1ZQotLS0KCmBgYHtyIHNldHVwLCBpbmNsdWRlPUZBTFNFfQprbml0cjo6b3B0c19jaHVuayRzZXQoZWNobyA9IEZBTFNFLCBtZXNzYWdlPUZBTFNFLCB3YXJuaW5nPUZBTFNFLAogICAgICAgICAgICAgICAgICAgICAgZHBpID0gOTYsIGZpZy53aWR0aCA9IDYsIGZpZy5oZWlnaHQgPSA0KQpgYGAKCmBgYHtyIGxpYnN9CiMgdGlkeSBzdHVmZgpsaWJyYXJ5KHRpZHl2ZXJzZSkgCmxpYnJhcnkoYnJvb20pIApsaWJyYXJ5KGtuaXRyKQoKIyBnZ3Bsb3QyICsgZXh0cmFzCmxpYnJhcnkoZ2diZWVzd2FybSkKbGlicmFyeShnZ3NpZ25pZikKCiMgRXh0cmEgdGVzdHMgYW5kIHBhaXJ3aXNlIGNvbXBhcmlzb25zCmxpYnJhcnkobXVsdGNvbXBWaWV3KQoKIyBNaXhlZCBtb2RlbHMgYW5kIGVzdGltYXRlZCBtYXJnaW5hbCBtZWFucwpsaWJyYXJ5KGxtZXJUZXN0KQpsaWJyYXJ5KGVtbWVhbnMpCmBgYAoKYGBge3IgbG9hZH0KIyBMb2FkIGRhdGEgZ2VuZXJhdGVkIGJ5IGNsZWFudXAgbm90ZWJvb2sKcm0obGlzdCA9IGxzKCkpCmxvYWQoJ2RhdGEvaWRyX3JlcGxhY2VtZW50X3NsYTEuUkRhdGEnKQpgYGAKCmBgYHtyIHRoZW1lfQojIEN1c3RvbSBnZ3Bsb3QyIHRoZW1lCiMgLS0tLS0tLS0tLS0tLS0tLS0tLS0KCiMgbWluaW1hbCB0aGVtZSB3aXRoIGJvcmRlcgojIGJhc2VkIG9uIHRoZW1lX2xpbmVkcmF3IHdpdGhvdXQgdGhlIGdyaWQgbGluZXMKIyBhbHNvIHRyeWluZyB0byByZW1vdmUgYWxsIGJhY2tncm91bmRzIGFuZCBtYXJnaW5zCiMgdGhlIGFpbSBpcyB0byBtYWtlIGl0IGFzIGVhc3kgYXMgcG9zc2libGUgdG8gZWRpdCBpbiBpbGx1c3RyYXRvcgoKdGhlbWVfY2xlYW4gPC0gZnVuY3Rpb24oYmFzZV9zaXplID0gMTEsIGJhc2VfZmFtaWx5ID0gIiIsCiAgICAgICAgICAgICAgICAgICAgICAgIGJhc2VfbGluZV9zaXplID0gYmFzZV9zaXplIC8gMjIsCiAgICAgICAgICAgICAgICAgICAgICAgIGJhc2VfcmVjdF9zaXplID0gYmFzZV9zaXplIC8gMjIpIHsKICB0aGVtZV9saW5lZHJhdygKICAgIGJhc2Vfc2l6ZSA9IGJhc2Vfc2l6ZSwKICAgIGJhc2VfZmFtaWx5ID0gYmFzZV9mYW1pbHksCiAgICBiYXNlX2xpbmVfc2l6ZSA9IGJhc2VfbGluZV9zaXplLAogICAgYmFzZV9yZWN0X3NpemUgPSBiYXNlX3JlY3Rfc2l6ZQogICkgJStyZXBsYWNlJQogICAgdGhlbWUoCiAgICAgICMgbm8gZ3JpZCBhbmQgbm8gYmFja2dyb3VuZHMgaWYgSSBjYW4gaGVscCBpdAogICAgICBsZWdlbmQuYmFja2dyb3VuZCA9ICBlbGVtZW50X2JsYW5rKCksCiAgICAgIHBhbmVsLmJhY2tncm91bmQgPSBlbGVtZW50X2JsYW5rKCksCiAgICAgIHBhbmVsLmdyaWQgPSBlbGVtZW50X2JsYW5rKCksCiAgICAgIHBsb3QuYmFja2dyb3VuZCA9IGVsZW1lbnRfYmxhbmsoKSwKICAgICAgcGxvdC5tYXJnaW4gPSBtYXJnaW4oMCwgMCwgMCwgMCksCiAgICAgIGNvbXBsZXRlID0gVFJVRQogICAgKQp9CgojIFNldCBkZWZhdWx0IHRoZW1lCiMgLS0tLS0tLS0tLS0tLS0tLS0KdGhlbWVfc2V0KHRoZW1lX2NsZWFuKGJhc2Vfc2l6ZSA9IDE0LCBiYXNlX2ZhbWlseSA9ICJNeXJpYWQgUHJvIikpCgojIENyZWF0ZSBhIGdnc2F2ZSB3cmFwcGVyCiMgLS0tLS0tLS0tLS0tLS0tLS0tLS0tLS0KCiMgVGhpcyB3YXkgd2UgY2FuIHNldCBhIGRlZmF1bHQgc2l6ZSBhbmQgZGV2aWNlIGZvciBhbGwgcGxvdHMKbXlfZ2dzYXZlIDwtIGZ1bmN0aW9uKGZpbGVuYW1lLCBwbG90ID0gbGFzdF9wbG90KCksCiAgICAgICAgICAgICAgICAgICAgICBkZXZpY2UgPSBjYWlyb19wZGYsIHVuaXRzID0gIm1tIiwKICAgICAgICAgICAgICAgICAgICAgIHdpZHRoID0gMTAwLCBoZWlnaHQgPSA4MCwgLi4uKXsKICBnZ3NhdmUoZmlsZW5hbWUgPSBmaWxlbmFtZSwgcGxvdCA9IHBsb3QsCiAgICAgICAgIGRldmljZSA9IGRldmljZSwgdW5pdHMgPSB1bml0cywKICAgICAgICAgaGVpZ2h0ID0gaGVpZ2h0LCB3aWR0aCA9IHdpZHRoLCAgLi4uKQogIH0KYGBgCgpgYGB7ciBmdW5jdGlvbnN9CiMgY291cGxlIG9mIHNtYWxsIGZ1bmN0aW9ucyBmb3IgZXh0cmFjdGluZyBjb21wYXJpc29ucwojIGludG8gYSBmb3JtYXQgdW5kZXJzdGFuZGFibGUgYnkgZ2dwbG90CgojJyBBZGQgbGV0dGVyIGdyb3VwIGxhYmVscyBnZW5lcmF0ZWQgYnkgVHVrZXkncyBIU0QKIycKIycgVGhpcyBmdW5jdGlvbiBwZXJmb3JtcyBBTk9WQSBhbmQgVHVrZXkncyBIU0QsCiMnIGV4dHJhY3RzIHRoZSBsZXR0ZXIgbGFiZWxzIGFuZCBhdHRhY2hlcyB0aGVtCiMnIHRvIHRoZSBvcmlnaW5hbCBkYXRhLgojJwojJyBAcGFyYW0geDogZGYgb3IgdGliYmxlLCB0aGUgZGF0YSAobG9uZyBmb3JtYXQhKQojJyBAcGFyYW0geXZhcjogY2hyLCBuYW1lIG9mIHRoZSBkZXBlbmRlbnQgdmFyaWFibGUKIycgQHBhcmFtIHh2YXI6IGNociwgbmFtZSBvZiB0aGUgaW5kZXBlbmRlbnQgdmFyaWFibGUKIycgQHBhcmFtIGFscGhhOiBkYmwsIGNvbmZpZGVuY2UgbGV2ZWwgcGFzc2VkIG9uIHRvIFR1a2V5J3MgdGVzdAojJwphZGRfdHVrZXlfbGFiZWxzIDwtIGZ1bmN0aW9uKHgsIHl2YXIsIHh2YXIsIGFscGhhID0gMC45NSl7CiAgYW92X2Zvcm0gPC0gZm9ybXVsYShwYXN0ZSh5dmFyLCAnficsIHh2YXIpKQogIGFub3ZhIDwtIGFvdihmb3JtdWxhID0gYW92X2Zvcm0sIGRhdGEgPSB4KQogIHR1a2V5IDwtIFR1a2V5SFNEKGFub3ZhLCB3aGljaCA9IHh2YXIsIGNvbmYubGV2ZWwgPSBhbHBoYSkKICAjIEV4dHJhY3QgbGFiZWxzIGFuZCBmYWN0b3IgbGV2ZWxzIGZyb20gVHVrZXkgcG9zdC1ob2MgCiAgeF9sYWJlbHMgPC0gYXNfdGliYmxlKAogICAgbXVsdGNvbXBMZXR0ZXJzNChhbm92YSwgdHVrZXkpW1t4dmFyXV0kTGV0dGVycywKICAgIHJvd25hbWVzID0geHZhcgogICAgKQogIAogIGlmIChpcy5mYWN0b3IoeFtbeHZhcl1dKSkgewogICAgeF9sYWJlbHNbW3h2YXJdXSA8LSBmYWN0b3IoCiAgICAgIHhfbGFiZWxzW1t4dmFyXV0sIGxldmVscyA9IGxldmVscyh4W1t4dmFyXV0pCiAgICApCiAgfQogIAogIHhfbGFiZWxzIDwtIHJlbmFtZSh4X2xhYmVscywgdHVrZXlfZ3JvdXAgPSB2YWx1ZSkKICB4IDwtIGxlZnRfam9pbih4LCB4X2xhYmVscywgYnkgPSB4dmFyKQogIAogIHJldHVybih4KQogIH0KCgojJyBFeHRyYWN0IGNvbXBhcmlzb25zIGZyb20gcnN0YXRpeCB0aWR5IHRlc3RzCiMnIAojJyBUaGlzIGZ1bmN0aW9uIHN1YnNldHMgdGhlIHNlbGVjdGVkIGNvbXBhcmlzb25zIGluIGEgVHVrZXksCiMnIER1bm4gb3Igc2ltaWxhciB0ZXN0IGRvbmUgYnkgcnN0YXRpeC4KIycgSXQgY29udmVydHMgZ3JvdXBzIHRvIGEgbGlzdCBvZiB2ZWN0b3JzIHRoYXQgY2FuIGJlIHBhc3NlZCB0byBnZW9tX3NpZ25pZgojJwojJwojJyBAcGFyYW0geDogZGYgb3IgdGliYmxlLCB0aGUgY29tcGFyaXNvbiByZXN1bHRzCiMnIEBwYXJhbSByb3dzOiBpbnRlZ2VyIHZlY3RvciwgdGhlIHJvd3Mgd2l0aCBkZXNpcmVkIGNvbXBhcmlzb25zCmV4dHJhY3RfY29tcGFyaXNvbnMgPC0gZnVuY3Rpb24oeCwgcm93cyl7CiAgeF9zdWJzZXQgPC0geFtyb3dzLF0gJT4lCiAgICAuW25yb3coLik6MSxdCiAgCiAgeF9jb21wYXJpc29ucyA8LSB4X3N1YnNldCAlPiUKICAgIHNlbGVjdChncm91cDEsIGdyb3VwMikgJT4lCiAgICB0KCkgJT4lCiAgICBhcy5kYXRhLmZyYW1lKCkgJT4lCiAgICBhcy5saXN0CiAgeF9hbm5vdGF0aW9ucyA8LSB4X3N1YnNldCRwLmFkai5zaWduaWYgJT4lCiAgICBhcy52ZWN0b3IoKQogIAogIHNpZ25pZmljYW5jZSA8LSBsaXN0KAogICAgY29tcGFyaXNvbnMgPSB4X2NvbXBhcmlzb25zLAogICAgYW5ub3RhdGlvbnMgPSB4X2Fubm90YXRpb25zCiAgKQogIAogIHJldHVybihzaWduaWZpY2FuY2UpCn0KYGBgCgojIHsudGFic2V0IC50YWJzZXQtcGlsbHN9CgojIyBFeHBlcmltZW50CgpUaGUgYWltIG9mIHRoZSBleHBlcmltZW50IGlzIHRvIGRldGVybWluZSB0aGUgZGVuc2l0eSBvZiBTbGExLUVHRlAgcGF0Y2hlcwppbiB5ZWFzdCBtdXRhbnRzIHdoZXJlIHRoZSBjZW50cmFsIHJlZ2lvbiBvZiBFZGUxIHByb3RlaW4KKGFtaW5vIGFjaWRzIDM2Ni05MDAsIFBRLXJpY2ggYW5kIGNvaWxlZC1jb2lsIGRvbWFpbnMpIAp3YXMgcmVwbGFjZWQgYnkgYSBoZXRlcm9sb2dvdXMgcHJvdGVpbiBkb21haW4sIG9uZSBvZjoKIAogLSB5ZWFzdCBTbmY1IHByaW9uLWxpa2UgSURSCiAtIHllYXN0IFN1cDM1IHByaW9uLWxpa2UgSURSCiAtIG1DaGVycnkgKG1vbm9tZXJpYyBmbHVvcmVzY2VudCBwcm90ZWluKQogLSBkVG9tYXRvIChkaW1lcmljIGZsdW9yZXNjZW50IHByb3RlaW4pCiAtIGtpbmVzaW4tMSBjb2lsZWQtY29pbCBmcm9tICpEcm9zb3BoaWxhIG1lbGFub2dhc3RlciogKGRpbWVyaWMpCiAtIGtpbmVzaW4tNSBjb2lsZWQtY29pbCBmcm9tICpIb21vIHNhcGllbnMqICh0ZXRyYW1lcmljKQoKIyMjIEltYWdpbmcKCkkgYWNxdWlyZWQgYWxsIGRhdGEgb24gdGhlIE9seW1wdXMgSVg4MQplcXVpcHBlZCB3aXRoIGEgMTAweC8xLjQ5IG9iamVjdGl2ZSwKdXNpbmcgdGhlIFgtQ2l0ZSAxMjBQQyBsYW1wIGF0IDUwJSBpbnRlbnNpdHkKYW5kIDQwMCBtcyBleHBvc3VyZSBmb3IgaWxsdW1pbmF0aW9uLgpMaWdodCB3YXMgZmlsdGVyZWQgdGhyb3VnaCBhIFUtTUdGUEhRIGZpbHRlciBjdWJlLgpJIGFjcXVpcmVkIHN0YWNrcyBvZiAyNiBwbGFuZXMgCndpdGggYSBzdGVwIHNpemUgb2YgMC4yIG1pY3JvbnMuCgojIyMgSW1hZ2UgcHJvY2Vzc2luZwoKSW5kaXZpZHVhbCBub24tYnVkZGluZyBjZWxscyB3ZXJlIGNyb3BwZWQgZnJvbSBmaWVsZHMgb2Ygdmlldy4KUGF0Y2ggbnVtYmVycyB3ZXJlIGV4dHJhY3RlZCB1c2luZyBQeXRob24gZnVuY3Rpb24gYGNvdW50X3BhdGNoZXNgCmZyb20gbXkgcGVyc29uYWwgcGFja2FnZSBgbWtpbWFnZWAKY29udGFpbmluZyBhIHNldCBvZiB3cmFwcGVycyBmb3IgYHNjaWtpdC1pbWFnZWAgZnVuY3Rpb25zLgpCcmllZmx5LCB0aGUgaW1hZ2VzIHdlcmUgbWVkaWFuLWZpbHRlcmVkIHdpdGggYSA1IHB4IGRpc2sgYnJ1c2gsCmFuZCB0aGUgZmlsdGVyZWQgaW1hZ2VzIHdlcmUgc3VidHJhY3RlZCBmcm9tIHRoZSBvcmlnaW5hbHMKdG8gc3VidHJhY3QgbG9jYWwgYmFja2dyb3VuZC4KVGhlIGJhY2tncm91bmQtc3VidHJhY3RlZCBpbWFnZXMgd2VyZSB0aHJlc2hvbGRlZCB1c2luZyB0aGUgWWVuIG1ldGhvZC4KVGhlIHRocmVzaG9sZGVkIGltYWdlcyB3ZXJlIGVyb2RlZCB1c2luZyB0aGUgbnVtYmVyIG9mIG5vbi16ZXJvCm5laWdoYm91cmluZyBwaXhlbHMgaW4gM0QgYXMgdGhlIGVyb3Npb24gY3JpdGVyaW9uLgpUaGUgc3BvdHMgd2VyZSBjb3VudGVkIHVzaW5nIHNraW1hZ2UubWVhc3VyZS5sYWJlbCgpIGZ1bmN0aW9uIHdpdGggMi1jb25uZWN0aXZpdHkuCgpDcm9zcy1zZWN0aW9uIGFyZWEgd2FzIG9idGFpbmVkIGJ5IAptZWRpYW4tZmlsdGVyaW5nIG9mIHRoZSBzdGFjayB3aXRoIDEwcHggZGlzayBicnVzaCwgCmNhbGN1bGF0aW5nIHRoZSBtYXhpbXVtIHByb2plY3Rpb24gaW1hZ2UsIAp0aHJlc2hvbGRpbmcgdXNpbmcgT3RzdSdzIGFsZ29yaXRobSwKYW5kIHVzaW5nIHNraW1hZ2UubWVhc3VyZS5yZWdpb25wcm9wcygpIHRvIG1lYXN1cmUgYXJlYS4KTm90ZSB0aGF0IHRoZXNlIGFyZSBwaXhlbCBjb3VudHMgb2YgKmNyb3NzLXNlY3Rpb24qIGFyZWEuIApUbyBkZXRlcm1pbmUgdGhlIHRvdGFsIHN1cmZhY2UgYXJlYSwgCkkgYXNzdW1lZCB0aGF0IGFuIHVuYnVkZGVkIGNlbGwgaXMgc3BoZXJpY2FsCihzdWZhY2UgYXJlYSBpcyBmb3VyIHRpbWVzIHRoZSBjcm9zcy1zZWN0aW9uIGFyZWEpLgoKVGhpcyBjYWxsIHRvIGBzaXRlX2NvdW50ZXJgIHdhcyB1c2VkIHRvIHByb2Nlc3MgYWxsIGRhdGFzZXRzOgoKYGBgCnByb2Nlc3NfZm9sZGVyKHBhdGgsIG1lZGlhbl9yYWRpdXMgPSA1LCBlcm9zaW9uX24gPSAxLCBjb24gPSAyLAogICAgICAgICAgICAgICAgICAgbWV0aG9kID0gWWVuLCBtYXNrID0gRmFsc2UsIGxvb3AgPSBGYWxzZSwgc2F2ZV9pbWFnZXMgPSBUcnVlKQpgYGAKCkFub3RoZXIgbm90ZWJvb2sgd2FzIHVzZWQgdG8gZ2F0aGVyIGFsbCBvdXRwdXQgaW50byB0aWR5IGRhdGEgZnJhbWVzCmF2YWlsYWJsZSBoZXJlLCB3aXRoIG5vIGZ1cnRoZXIgbW9kaWZpY2F0aW9ucy4KCiMjIyBTdHJhaW4gbGlzdAoKYGBge3J9CmthYmxlKHN0cmFpbnMpCmBgYAoKIyMgUGVyLWRhdGFzZXQgc3VtbWFyeSB7LnRhYnNldH0KCiMjIyBQYXRjaCBudW1iZXIgYW5kIGFyZWEKCmBgYHtyIG51bXNfZGF0YXNldH0KIyBtZWFucyBhbmQgU0RzIG9mIG51bWJlciBvZiBwYXRjaGVzIGFuZCBhcmVhCnNsYTFfZGVuc2l0eSAlPiUKICBncm91cF9ieShlZGUxLCBkYXRhc2V0KSAlPiUKICBzdW1tYXJpc2UobiA9IG4oKSwKICAgICAgICAgICAgYWNyb3NzKGMocGF0Y2hlcywgYXJlYSksIGxpc3QobWVhbiA9IG1lYW4sIHNkID0gc2QsCiAgICAgICAgICAgICAgICAgICAgICAgICAgICAgICAgICAgICAgICAgIHNlID0gfnNkKC54KSAvIHNxcnQobigpKSkpKSAlPiUKICBrYWJsZSgpCmBgYAoKIyMjIFNsYTEgZGVuc2l0eQoKV2UgY2FuIGNvbWJpbmUgdGhlIHBhdGNoIG51bWJlciBhbmQgYXJlYSBpbnRvCiRkZW5zaXR5ID0gXGZyYWN7cGF0Y2hlc317YXJlYX0kLApjYWxjdWxhdGVkIGluZGl2aWR1YWxseSBmb3IgZWFjaCBjZWxsLgpXZSBjYW4gc3VtbWFyaXNlIHRoZSBkYXRhIApmb3IgZWFjaCBFZGUxIG11dGFudCBpbiBlYWNoIGRhdGFzZXQ6CgpgYGB7ciBkZW5zaXR5fQpzbGExX2RlbnNpdHlfc3RhdHMgPC0gc2xhMV9kZW5zaXR5ICU+JQogIGdyb3VwX2J5KGVkZTEsIGRhdGFzZXQpICU+JQogIHN1bW1hcmlzZShuID0gbigpLAogICAgICAgICAgICBhY3Jvc3MoZGVuc2l0eSwKICAgICAgICAgICAgICAgICAgIGxpc3QobWVhbiA9IG1lYW4sIHNkID0gc2QsIAogICAgICAgICAgICAgICAgICAgICAgICBzZSA9IH4gc2QoLngpIC8gc3FydChuKSwKICAgICAgICAgICAgICAgICAgICAgICAgbWVkaWFuID0gbWVkaWFuLCBtYWQgPSBtYWQpKSwKICAgICAgICAgICAgLmdyb3VwcyA9ICdkcm9wJykKCnNsYTFfZGVuc2l0eV9zdGF0cyAlPiUga2FibGUoKQpgYGAKCiMjIFBsb3RzIHsudGFic2V0fQoKYGBge3IgZGVuc2l0eS5zY2F0dGVyfQpwbG90X2JsYW5rIDwtIGdncGxvdChzbGExX2RlbnNpdHlfc3RhdHMsCiAgICAgICAgICAgICAgICAgICAgIGFlcyh4ID0gZWRlMSwgeSA9IGRlbnNpdHlfbWVhbikpICsKICAgICAgICAgICAgICAgICAgICAgICAgICNzaGFwZSA9IGRhdGFzZXQsIGZpbGwgPSBkYXRhc2V0KSkrCiAgbGFicyh0aXRsZSA9IE5VTEwsIHggPSAnRWRlMScsIHkgPSBleHByZXNzaW9uKCAiU2xhMSBwYXRjaGVzL8K1bSJeMikpICsKICBzY2FsZV95X2NvbnRpbnVvdXMoYnJlYWtzID0gc2VxKDAuMCwgMS4wLCAwLjEpKSArCiAgc2NhbGVfc2hhcGVfbWFudWFsKHZhbHVlcyA9IGMoMjE6MjUsIDgpKSArCiAgc2NhbGVfY29sb3JfYnJld2VyKHBhbGV0dGUgPSAnU2V0MicpICsKICBzY2FsZV9maWxsX2JyZXdlcihwYWxldHRlID0gJ1NldDInKQoKcGxvdF9zY2F0dGVyIDwtIHBsb3RfYmxhbmsgKwogIGdlb21fcXVhc2lyYW5kb20oaW5oZXJpdC5hZXMgPSBGLCBkYXRhID0gc2xhMV9kZW5zaXR5LAogICAgICAgICAgICAgICAgICAgYWVzKHggPSBlZGUxLCB5ID0gZGVuc2l0eSwKICAgICAgICAgICAgICAgICAgICAgICBzaGFwZSA9IGRhdGFzZXQsIyBjb2xvdXIgPSBkYXRhc2V0CiAgICAgICAgICAgICAgICAgICAgICAgKSwKICAgICAgICAgICAgICAgICAgIGNvbG91ciA9ICdncmV5NzUnLCMgc2hhcGUgPSAxLAogICAgICAgICAgICAgICAgICAgc2hvdy5sZWdlbmQgPSBGLCBzaXplID0gMC44CiAgICAgICAgICAgICAgICAgICApCgpwbG90X3Zpb2xpbiA8LSBwbG90X2JsYW5rICsgCiAgZ2VvbV92aW9saW4oaW5oZXJpdC5hZXMgPSBGLAogICAgICAgICAgICAgIGRhdGEgPSBzbGExX2RlbnNpdHksIGFlcyh4ID0gZWRlMSwgeSA9IGRlbnNpdHkpLAogICAgICAgICAgICAgIGNvbG91ciA9ICdncmV5NzUnLCBmaWxsID0gJ3RyYW5zcGFyZW50JwogICAgICAgICAgICAgICkKYGBgCgojIyMgU3VwZXJQbG90cwoKSSBoYXZlIGNob3NlbiB0byBzaG93IHRoaXMgZGF0YSB1c2luZyB0aGUgCltTdXBlclBsb3RdKGh0dHBzOi8vZG9pLm9yZy8xMC4xMDgzL2pjYi4yMDIwMDEwNjQpIHN0eWxlLgpFYWNoIHBvaW50IHNob3dzIGRlbnNpdHkgb2YgU2xhMS1FR0ZQIHBhdGNoZXMgaW4gYW4gaW5kaXZpZHVhbCBjZWxsLgoKQmlnIGNvbG91ciBwb2ludHMgc2hvdyBtZWFuIG1lYXN1cmVtZW50cyBmcm9tIHRocmVlIGluZGVwZW5kZW50IHJlcGVhdHMuCgpSYW5nZSBpcyBtZWFuICsvLSBTRCwgY2FsY3VsYXRlZCBiYXNlZCBvbiB0aGUgdGhyZWUgaW5kZXBlbmRlbnQgcmVwZWF0IG1lYW5zLgoKIyMjIyBCZWVzd2FybQoKYGBge3J9CnBsb3Rfc3VwZXIgPC0gcGxvdF9zY2F0dGVyICsKICBnZW9tX3F1YXNpcmFuZG9tKGFlcyhzaGFwZSA9IGRhdGFzZXQsIGZpbGwgPSBkYXRhc2V0KSwKICAgICAgICAgICAgICAgICAgIHNob3cubGVnZW5kID0gRiwKICAgICAgICAgICAgICAgICAgIHdpZHRoID0gMC4zLCBzaXplID0gMikrCiAgc3RhdF9zdW1tYXJ5KGZ1biA9IG1lYW4sIGdlb20gPSAnY3Jvc3NiYXInLAogICAgICAgICAgICAgICB3aWR0aCA9IDAuNSwgZmF0dGVuID0gMSkrCiAgc3RhdF9zdW1tYXJ5KGZ1bi5kYXRhID0gJ21lYW5fc2RsJywKICAgICAgICAgICAgICAgZnVuLmFyZ3MgPSBsaXN0KG11bHQgPSAxKSwgCiAgICAgICAgICAgICAgIGdlb20gPSAnZXJyb3JiYXInLCB3aWR0aCA9IDAuMikrCiAgZ3VpZGVzKHggPSBndWlkZV9heGlzKGFuZ2xlID0gLTQ1KSkKICAKcHJpbnQocGxvdF9zdXBlcikKbXlfZ2dzYXZlKCdmaWd1cmVzL2RlbnNpdHlfc3VwZXIucGRmJykKCnBsb3Rfc3VwZXJfZmxpcCA8LSBwbG90X3N1cGVyICsKICBjb29yZF9mbGlwKCkrCiAgc2NhbGVfeF9kaXNjcmV0ZShsaW1pdHMgPSByZXYobGV2ZWxzKHNsYTFfZGVuc2l0eSRlZGUxKSkpKwogIHRoZW1lKHBhbmVsLmJvcmRlciA9IGVsZW1lbnRfYmxhbmsoKSwKICAgICAgICBheGlzLmxpbmUueSA9IGVsZW1lbnRfYmxhbmsoKSwKICAgICAgICBheGlzLnRpY2tzLnkgPSBlbGVtZW50X2JsYW5rKCksCiAgICAgICAgYXhpcy5saW5lLnggPSBlbGVtZW50X2xpbmUoKSkKCm15X2dnc2F2ZSgnZmlndXJlcy9kZW5zaXR5X3N1cGVyX2ZsaXAucGRmJywgcGxvdF9zdXBlcl9mbGlwLAogICAgICAgICAgaGVpZ2h0ID0gMTAwLCB3aWR0aCA9IDgwKQpgYGAKCiMjIyMgVmlvbGluCgpgYGB7cn0KcGxvdF9zdXBlcl92aW9saW4gPC0gcGxvdF92aW9saW4gKwogIGdlb21fcXVhc2lyYW5kb20oYWVzKHNoYXBlID0gZGF0YXNldCwgZmlsbCA9IGRhdGFzZXQpLAogICAgICAgICAgICAgICAgICAgc2hvdy5sZWdlbmQgPSBGLAogICAgICAgICAgICAgICAgICAgd2lkdGggPSAwLjMsIHNpemUgPSAyKSsKICBzdGF0X3N1bW1hcnkoZnVuID0gbWVhbiwgZ2VvbSA9ICdjcm9zc2JhcicsCiAgICAgICAgICAgICAgIHdpZHRoID0gMC41LCBmYXR0ZW4gPSAxKSsKICBzdGF0X3N1bW1hcnkoZnVuLmRhdGEgPSAnbWVhbl9zZGwnLAogICAgICAgICAgICAgICBmdW4uYXJncyA9IGxpc3QobXVsdCA9IDEpLAogICAgICAgICAgICAgICBnZW9tID0gJ2Vycm9yYmFyJywgd2lkdGggPSAwLjIpCgpwcmludChwbG90X3N1cGVyX3Zpb2xpbikKbXlfZ2dzYXZlKCdmaWd1cmVzL2RlbnNpdHlfc3VwZXJfdmlvbGluLnBkZicpCmBgYAoKIyMjIFdpdGggc2lnbmlmaWNhbmNlCgpUaGVyZSBhcmUgOSBsZXZlbHMgb2YgRWRlMSwKcmVzdWx0aW5nIGluIDM2IHBvc3NpYmxlIHBhaXJ3aXNlIGNvbXBhcmlzb25zLgpUaGlzIGlzIHRvbyB1bndpZWxkeSBmb3IgYW55IHBsb3QsCmF0IGxlYXN0IGEgcGxvdCBpbnRlbmRlZCB0byBhbHNvIHNob3cgdGhlIGRhdGEuCgpBcyBzdWNoLCBJIHByb3Bvc2UgdG8gbm90IGV2ZW4gYXR0ZW1wdAp0aGlzIGtpbmQgb2YgaWxsdXN0cmF0aW9uLgpBbiBhbHRlcm5hdGl2ZSBpcyBhIGNvbXBhY3QgbGV0dGVyIGRpc3BsYXkuCkluIHRoaXMgdmlldywgZ3JvdXBzIHNoYXJpbmcgYXQgbGVhc3Qgb25lIGxldHRlciAKYXJlIG5vdCBzaWduaWZpY2FudGx5IGRpZmZlcmVudAphdCBhIGNob3NlbiAkXGFscGhhJCAoaGVyZSwgNSUpLgoKUHJvczoKCiAgKiBsZXNzIGNsdXR0ZXJlZCBhbmQgc2ltcGxlciB0byByZWFkCiAgKiBmb2N1cyBhd2F5IGZyb20gcC12YWx1ZXM7IHByb3ZpZGUgYSBiaW5hcnkgZGVjaXNpb24gb24gdGhlIG51bGwgaHlwb3RoZXNpcwogIApDb25zOgoKICAqIGNhbm5vdCBkaXN0aW5ndWlzaCBkaWZmZXJlbnQgY29uZmlkZW5jZSBsZXZlbHMKICAqIGNhbiBnZXQgY29tcGxpY2F0ZWQganVzdCBhcyB3ZWxsCiAgKiBmb2N1c2VzIG9uIG5vbi1maW5kaW5ncyByYXRoZXIgdGhhbiBmaW5kaW5ncy4KCmBgYHtyfQpzbGExX2RlbnNpdHlfc3RhdHMgPC0gc2xhMV9kZW5zaXR5X3N0YXRzICU+JQogIGFkZF90dWtleV9sYWJlbHMoJ2RlbnNpdHlfbWVhbicsICdlZGUxJykKYGBgCgpgYGB7cn0KcGxvdF9zdXBlcl9sZXR0ZXJzIDwtIHBsb3Rfc3VwZXIgKwogIGdlb21fdGV4dChkYXRhID0gc2xhMV9kZW5zaXR5X3N0YXRzLAogICAgICAgICAgICBhZXMobGFiZWwgPSB0dWtleV9ncm91cCksIHkgPSBJbmYsIHZqdXN0ID0gMS4yKQogICNzdGF0X3N1bW1hcnkoYWVzKGxhYmVsID0gdHVrZXlfZ3JvdXApLAogICMgICAgICAgICAgICAgZnVuLnkgPSBJbmYsCiAgIyAgICAgICAgICAgICBnZW9tID0gJ3RleHQnLCBuYS5ybSA9IFQsIHZqdXN0ID0gLTAuNSwKICAjICAgICAgICAgICAgICNmdW4uYXJncyA9IGxpc3QoZXJyID0gZXJyb3JzLCBtdWx0ID0gZXJyb3JfcmFuZ2UpCiAgIyAgICAgICAgICAgICApCnByaW50KHBsb3Rfc3VwZXJfbGV0dGVycykKbXlfZ2dzYXZlKCdmaWd1cmVzL2RlbnNpdHlfc3VwZXJfbGV0dGVycy5wZGYnLCB3aWR0aCA9IDE1MCkKYGBgCgpBcyB0aGlzIGlzIGFsc28gY29tcGxpY2F0ZWQsCkkgcHJvcG9zZSBhIGZpbmFsIHNvbHV0aW9uOgppbmRpY2F0ZSB0aGF0IGFsbCBncm91cHMgYXJlIHNpZ25pZmljYW50bHkgZGlmZmVyZW50CmZyb20gd2lsZC10eXBlIGluIHRoZSB0ZXh0LAphbmQgbWFyayB0aG9zZSB3aXRoIGEgc2lnbmlmaWNhbnQgaW1wcm92ZW1lbnQgZnJvbSAKZWRlMeKIhlBRQ0Mgd2l0aCBhIHN0YXIsCmFsbCB3aGlsZSBwcm92aWRpbmcgYSB0YWJsZSBvZiBwLXZhbHVlcwpmb3IgZWFjaCBwYWlyd2lzZSBjb21wYXJpc29uIGluIHRoZSBzdXBwbGVtZW50LgoKIyMjIFBvb2xlZCBib3ggcGxvdAoKVGhpcyBtaWdodCBiZSB1c2VmdWwgaW4gc2l0dWF0aW9ucwp3aGVyZSB0aGlzIG11Y2ggZGF0YSBpcyB0b28gbXVjaCBkYXRhLgoKYGBge3J9CnBsb3RfYm94IDwtIHBsb3RfYmxhbmsgKyAKICBnZW9tX2JveHBsb3QoaW5oZXJpdC5hZXMgPSBGLCBkYXRhID0gc2xhMV9kZW5zaXR5LAogICAgICAgICAgICAgICBhZXMoeCA9IGVkZTEsIHkgPSBkZW5zaXR5KSwKICAgICAgICAgICAgICAgb3V0bGllci5zaGFwZSA9IDIxLCB3aWR0aCA9IDAuNSwgbm90Y2ggPSBUKSsKICBndWlkZXMoeCA9IGd1aWRlX2F4aXMoYW5nbGUgPSAtNDUpKQoKcHJpbnQocGxvdF9ib3gpCmBgYAoKIyMgTW9kZWxpbmcKCldlIHdhbnQgdG8gY29uc2lkZXI6CgogIDEuIFdoYXQgYXJlIHRoZSBTbGExIGRlbnNpdGllcyBpbiBkaWZmZXJlbnQgRWRlMSBiYWNrZ3JvdW5kcz8KICAyLiBBcmUgdGhlIGRpZmZlcmVuY2VzIGJldHdlZW4gc3RyYWlucyBzdGF0aXN0aWNhbGx5IHNpZ25pZmljYW50PwoKVGhlIGRlc2lnbiBpcyBub3QgaWRlYWwgaGVyZToKCiAgLSB3ZSB3YW50IHRvIGNvbXBhcmUgYWxsIG11dGFudHMgYWdhaW5zdCBlYWNoIG90aGVyCiAgLSB3ZSB3YW50IHRvIGJsb2NrIGZvciBudWlzYW5jZSBmYWN0b3JzLAogIHdoaWNoIG1pZ2h0IGNvbWUgZnJvbSBjby1jdWx0dXJpbmcgdGhlIGNlbGxzCiAgb3IgdGhpbmdzIHJlbGF0ZWQgdG8gdGhlIG1pY3Jvc2NvcGUgc3VjaCBhcyBsYW1wIHBvd2VyCiAgaGVuY2UgdGhlIGluZGVwZW5kZW50bHkgcmVwZWF0ZWQgZXhwZXJpbWVudHMKICAtIGhhbGYgdGhlIGV4cGVyaW1lbnQKICB3YXMgYWRkZWQgKmFmdGVyKiB0aGUgcmV2aWV3ZXJzIHJlcXVlc3QsCiAgc28gdGhlIGRlc2lnbiBpcyBkaXNjb25uZWN0ZWQuIAogIERhdGFzZXRzIDEtMyBoYXZlIGJvdGggY29udHJvbHMgYW5kIElEUnMsCiAgZGF0YXNldHMgNC02IGhhdmUgdGhlIGdsb2J1bGFyIC8gY29pbGVkLWNvaWwgcmVwbGFjZW1lbnRzLgogIEZpbmFsbHksIHRoZSBtQ2hlcnJ5IHN0cmFpbiB3YXMgYWRkZWQgCiAgYWZ0ZXIgYWxsIG9mIHRoZSBvdGhlcnMgb24gc2VwYXJhdGUgZGF0ZXMuCiAgCkJhZCBkZXNpZ24gY2Fubm90IGJlIHNvbHZlZAppbiBzdGF0aXN0aWNhbCBhbmFseXNpcywKYnV0IG9uIHRoZSBicmlnaHQgc2lkZSwgdGhlIGRhdGEgaXMgbmljZQphbmQgdGhlcmUgaXMgbm8gcmVhc29uIGZvciBhIHN5c3RlbWF0aWMgZXJyb3IgdG8gYmUKYXNzb2NpYXRlZCB3aXRoIGFueSBwYXJ0aWN1bGFyIGRhdGFzZXQuClNpbmNlIHdlIGFyZSBtZWFzdXJpbmcgYW4gYWJzb2x1dGUgcHJvcGVydHkgKG51bWJlciBvZiBzaXRlcyksCmFuZCBub3Qgc29tZXRoaW5nIGRpcmVjdGx5IHByb3BvcnRpb25hbAp0byBleHBvc3VyZSBzZXR0aW5ncyBsaWtlIGludGVuc2l0eSwKdGhlIGluZmx1ZW5jZSBvZiBsYW1wIHBvd2VyIGV0Yy4gc2hvdWxkIGJlIGxpbWl0ZWQKCklmIHRoZSBleHBlcmltZW50IHdhcyBub3QgZGlzY29ubmVjdGVkLAp3ZSBjb3VsZCB0cnkgdG8gc2VlIGlmIHRoZSBlZmZlY3RzIG9mIGRhdGUgLyBkYXRhc2V0CmNhbiBiZSBtb2RlbGVkLgoKQmVjYXVzZSBpdCAqaXMqIGRpc2Nvbm5lY3RlZCwgaXQncyBhIGJpdCBtb3JlIHRyaWNreS4KRm9yIGV4YW1wbGUgd2UgY2Fubm90IHJlYWxseSBnZXQgdXNlZnVsIGluZm9ybWF0aW9uCmZyb20gdHJ5aW5nIHRvIG1vZGVsIGBkYXRlYCBhcyBhIGZpeGVkIGVmZmVjdCBhZnRlciBgZWRlMWAuCldoYXQgd2UgY291bGQgZG8gZm9yIHN0YXJ0ZXJzIGlzIHRvIG1ha2UgYSBtb2RlbAp3aGljaCBkaXNyZWdhcmRzIHRoZSBkYXRhc2V0IGluZm9ybWF0aW9uLCAKYW5kIGxvb2sgaWYgdGhlIHJlc2lkdWFscyBhcmUgY2x1c3RlcmVkIGJ5IGRhdGUgb3IgZGF0YXNldCwKaW5mb3JtaW5nIHVzIGFib3V0IHdoZXRoZXIgdGhpcyBhY3R1YWxseSBtYXR0ZXJzLgoKYGBge3IgZWNobyA9IFRSVUV9CnBvb2xlZF9sbSA8LSBsbShkZW5zaXR5IH4gZWRlMSwgZGF0YSA9IHNsYTFfZGVuc2l0eSkKcGxvdChhcy5mYWN0b3Ioc2xhMV9kZW5zaXR5JGRhdGUpLCByZXNpZChwb29sZWRfbG0pLAogICAgIHhsYWIgPSAnJywgeWxhYiA9ICdSZXNpZHVhbHMnLCBsYXMgPSAyLAogICAgIG1haW4gPSAnVW5pdmFyaWF0ZSBtb2RlbCByZXNpZHVhbHMgYnkgZGF0ZScpCmBgYAoKVGhlc2Ugc2VlbSB0byBiZSB2ZXJ5IHdlbGwgZGlzdHJpYnV0ZWQgYXJvdW5kIHplcm8uClRoZSBleGNlcHRpb24gaXMgdGhlIHNpbmdsZSBtQ2hlcnJ5IGFjcXVpc2l0aW9uCm9uIDEwLzAyLzIwMjIsIHdoaWNoLCB0byBiZSBob25lc3QsCmRvZXMgc2VlbSBsaWtlIGFuIG91dGxpZXIuCgpTbyB3aGF0IHRvIGRvPyBUaGUgZGVjaXNpb24gc2VlbXMgZmFpcmx5IGFyYml0cmFyeS4KSSBkbyBub3QgbmVjZXNzYXJpbHkgdGhpbmsgdGhhdCB0aGUgdW5pdmFyaWF0ZQptb2RlbCBvbiBhbGwgY2VsbHMgd291bGQgYmUgKndyb25nKiwKYnV0IGl0IGRvZXMgc2VlbSBsaWtlIGEgY29udHJvdmVyc2lhbCBpc3N1ZS4KQSBtb2RlbCBvZiByZXBsaWNhdGUgbWVhbnMgKCptZWFucy1wb29sZWQgYW5hbHlzaXMqKQp3aWxsIGJlIGNvbnNlcnZhdGl2ZSBhbmQgbG9zZSBwb3dlciwKYnV0IGl0IHNlZW1zIGFwcHJvcHJpYXRlIGZvciBvbmNlLCAKY29uc2lkZXJpbmcgdGhlIHNlZ3JlZ2F0ZWQgZGVzaWduLgoKIyMjIE1lYW5zLXBvb2xlZCBsaW5lYXIgbW9kZWwgQU5PVkEKCldlIHdpbGwgdGVzdCB0aGUgbnVsbCBoeXBvdGhlc2lzIAp0aGF0IG1lYW4gU2xhMSBkZW5zaXR5IGlzIHRoZSBzYW1lCmFjcm9zcyBkaWZmZXJlbnQgRWRlMSBzdHJhaW5zLgpXZSB3aWxsIHVzZSByZXBlYXQtbGV2ZWwgZGF0YSBmb3IgdGhlIHRlc3RzCnRvIGFjY291bnQgZm9yIGV4cGVyaW1lbnRhbCB2YXJpYWJpbGl0eS4KCldlIGNhbiB1c2UgQU5PVkEgYW5kIGEgcG9zdC1ob2MgdGVzdCAKdG8gZmluZCBvdXQgaG93IGxpa2VseSB0aGlzIGRhdGEgaXMgdG8gb2NjdXIKdW5kZXIgdGhlIG51bGwgaHlwb3RoZXNpcy4KCk1vcmUgaW1wb3J0YW50bHksCndlIHdpbGwgdHJ5IHRvIGZpbmQgdGhlIGVmZmVjdCBzaXplcwpvZiBkaWZmZXJlbnQgY29udHJhc3RzLgoKV2Ugc3RhcnQgYnkgbWFraW5nIGEgbW9kZWwKb2YgYGRlbnNpdHlfbWVhbiB+IGVkZTFgOgoKYGBge3J9CnJlcGxpY2F0ZV9sbSA8LSBsbShkZW5zaXR5X21lYW4gfiBlZGUxLCBkYXRhID0gc2xhMV9kZW5zaXR5X3N0YXRzKQpzdW1tYXJ5KHJlcGxpY2F0ZV9sbSkKYW5vdmEocmVwbGljYXRlX2xtKQpgYGAKCk9uZS13YXkgQU5PVkEgb24gbWVhbnMtcG9vbGVkIGRhdGEgCnJlamVjdHMgdGhlIG51bGwgd2l0aCAkcCA9IDEuNSBcdGltZXMgMTBeey04fSQuCgojIyMjIERpYWdub3N0aWMgcGxvdHMKCmBgYHtyfQpwbG90KHJlcGxpY2F0ZV9sbSkKYGBgCgpUaGlzIGlzIG9uZSBwcm9ibGVtIHdpdGggdGhlIHNtYWxsIE4gb2YgcmVwbGljYXRlIG1lYW5zOgp0aGVyZSBhcmUgZGVmaW5pdGVseSBkaWZmZXJlbmNlcyBpbiB2YXJpYW5jZQpmb3IgZGlmZmVyZW50IGxldmVscywgYnV0IHdlIGNhbm5vdCB0ZWxsCndoZXRoZXIgdGhleSByZWZsZWN0IGEgZGlmZmVyZW5jZSBpbiBwb3B1bGF0aW9ucwpvciBqdXN0IG5vaXNlLiBCdXQgaXQncyBwcm9iYWJseSB0aGUgbGF0dHRlcjsKaWYgd2UgZ28gYmFjayB0byB0aGUgY2VsbC1sZXZlbCBvYnNlcnZhdGlvbnMKbW9kZWxlZCBieSBgcG9vbGVkX2xtYCB3ZSBjYW4gc2VlIHRoYXQgdGhlIHJlc2lkdWFscwphcmUgZmluZSB0aGVyZSwgdG9vOgoKYGBge3J9CnBsb3QocG9vbGVkX2xtKQpgYGAKCiMjIyBFc3RpbWF0ZWQgZ3JvdXAgbWVhbnMKClRoZSBmb2xsb3dpbmcgYXJlIHRoZSBtb2RlbCBlc3RpbWF0ZXMgCm9mIG1lYW4gZGVuc2l0aWVzIGFzc29jaWF0ZXMgd2l0aCBlYWNoIEVkZTEgbGV2ZWw6CgpgYGB7cn0KcmVwbGljYXRlX2VtbSA8LSBlbW1lYW5zKHJlcGxpY2F0ZV9sbSwgc3BlYyA9ICdlZGUxJykKc3VtbWFyeShyZXBsaWNhdGVfZW1tKQpgYGAKCiMjIyBQb3N0LWhvYyB0ZXN0IChUdWtleSkKCldlIGFyZSBub3QgbmVjZXNzYXJpbHkgaW50ZXJlc3RlZCBpbiBldmVyeSBwb3NzaWJsZSBjb21wYXJpc29uLApidXQgd2UgZG8gd2FudCB0byBvYnRhaW4gYWxsIHRoZSBwLXZhbHVlcyAKYXQgbGVhc3QgYWdhaW5zdCDiiIZQUUNDIChpcyB0aGVyZSBhIHJlc2N1ZT8pIGFuZCB3aWxkLXR5cGUgKGlzIGl0IGNvbXBsZXRlPykuClR1a2V5J3MgdGVzdCBmb3IgY29udHJhc3RzIG9mIGFsbCA5IGVzdGltYXRlczoKCmBgYHtyfQpjb250cmFzdChyZXBsaWNhdGVfZW1tLCBtZXRob2QgPSAncGFpcndpc2UnKQpgYGAKClRoaXMgaXMgYSBsb3Q7IHBlcmhhcHMgd2UgY2FuIHB1dCBpdCBpbiBhIGdyYXBoaWNhbCBtYXRyaXguCgojIyMjIHAtdmFsdWUgbWF0cml4CgpgYGB7ciBmaWcud2lkdGg9OH0KcF9tYXRyaXggPC0gdGlkeShjb250cmFzdChyZXBsaWNhdGVfZW1tLCBtZXRob2QgPSAncGFpcndpc2UnKSklPiUKICBzZXBhcmF0ZShjb250cmFzdCwgYygnZ3IxJywgJ2dyMicpLCAnIC0gJykgJT4lCiAgc2VsZWN0KGdyMSwgZ3IyLCBhZGoucC52YWx1ZSkgJT4lCiAgbXV0YXRlKGdyMSA9IGZjdF9pbmZyZXEoZ3IxKSwgZ3IyID0gZmN0X2luZnJlcShncjIpKQoKcF9tYXRyaXhfcGxvdCA8LSBnZ3Bsb3QocF9tYXRyaXgsIGFlcyhncjEsIGdyMikpKwogIGdlb21fdGlsZShhZXMoZmlsbCA9IGFkai5wLnZhbHVlIDwgMC4wNSksIHNob3cubGVnZW5kID0gRikrCiAgZ2VvbV90ZXh0KGFlcyhsYWJlbCA9IGlmX2Vsc2UoYWRqLnAudmFsdWUgPCAwLjAwMSwKICAgICAgICAgICAgICAgICAgICAgICAgICAgICAgICAnPDAuMDAxJywKICAgICAgICAgICAgICAgICAgICAgICAgICAgICAgICBhcy5jaGFyYWN0ZXIocm91bmQoYWRqLnAudmFsdWUsIDMpKSkpKSsKICBsYWJzKHggPSBOVUxMLCB5ID0gTlVMTCkrCiAgI3RoZW1lX2NsZWFuKCkrCiAgdGhlbWUocGFuZWwuYm9yZGVyID0gZWxlbWVudF9ibGFuaygpLAogICAgICAgIGF4aXMudGlja3MgPSBlbGVtZW50X2JsYW5rKCksCiAgICAgICAgbGVnZW5kLnRpdGxlID0gZWxlbWVudF90ZXh0KGZhY2UgPSAnYm9sZCcpKSsKICBzY2FsZV9maWxsX2JyZXdlcigpCnByaW50KHBfbWF0cml4X3Bsb3QpCm15X2dnc2F2ZSgnZmlndXJlcy9wX21hdHJpeF9wbG90LnBkZicsIHdpZHRoID0gMTUwKQpgYGAKCiMjIyMgVGhlIGJvdHRvbSBsaW5lCgoqIGV2ZXJ5IG11dGFudCBpcyBzaWduaWZpY2FudGx5IGRpZmZlcmVudCBmcm9tIHdpbGQtdHlwZSBhdCAkXGFscGhhID0gMC4wNSQKKiBvbmx5IFN1cDM1IHJlcGxhY2VtZW50IGlzIGEgc2lnbmlmaWNhbnQgaW1wcm92ZW1lbnQgb3ZlciDiiIZQUUNDCgojIyMgRWZmZWN0IHNpemVzCgpFZmZlY3Qgc2l6ZXMgYXJlIHBlcmhhcHMgbW9yZSBpbXBvcnRhbnQgdGhhbiB0aGUgcC12YWx1ZXMuCkhlcmUgSSB3aWxsIGNhbGN1bGF0ZSB0aGUgY2xhc3NpY2FsIENvaGVuJ3MgZCwKZGVmaW5lZCBhcyB0aGUgbWVhbiBkaWZmZXJlbmNlcyBvdmVyIHBvcHVsYXRpb24gc3RhbmRhcmQgZGV2aWF0aW9uLgpEZXNwaXRlIHVzaW5nIG1lYW5zLXBvb2xlZCBtb2RlbAp0byBlc3RpbWF0ZSBjb250cmFzdCBwLXZhbHVlcyBhbmQgcG9wdWxhdGlvbiBtZWFucywKSSBhbSBjb25maWRlbnQgdGhhdCBpdCBpcyBtb3JlIGFwcHJvcHJpYXRlCnRvIHRha2UgdGhlIHBvb2xlZCBvYnNlcnZhdGlvbi1sZXZlbCBTRApmb3IgY2FsY3VsYXRpbmcgdGhlIGVmZmVjdCBzaXplLgoKVGhlcmVmb3JlICRcc2lnbWEkIHdpbGwgYmUgYSBtZWFuIG9mIFNEIGZvciBlYWNoIEVkZTEgbGV2ZWwsCndlaWdoZWQgYnkgJG4tMSQgCihkZXBhcnRpbmcgZnJvbSBgZW1tZWFuc2AgZGVmYXVsdCBzdWdnZXN0aW9uIG9mIHJlc2lkdWFsIFNEKS4KSSBhbSBub3QgcXVpdGUgc3VyZSB3aGF0IHRoZSBhcHByb3ByaWF0ZSBkZWdyZWVzIG9mIGZyZWVkb20gYXJlLApJIHVzZWQgc2FtcGxlIHNpemUgbWludXMgRWRlMSBsZXZlbCBjb3VudC4gCkluIGFueSBjYXNlLCBpdCBhZmZlY3RzIHRoZSA5NSUgQ0kncyBvZiBlZmZlY3Qgc2l6ZSBlc3RpbWF0ZSwKYnV0IG5vdCB0aGUgZXN0aW1hdGUgaXRzZWxmLgoKQmVsb3cgYXJlIGVmZmVjdCBzaXplcyBjYWxjdWxhdGVkIGZvciBjb21wYXJpc29ucyBhZ2FpbnN0IHdpbGQtdHlwZQphbmQgYWdhaW5zdCDiiIZQUUNDLgoKYGBge3J9CnBvb2xlZF92YXIgPC0gd2l0aChzbGExX2RlbnNpdHksCiAgICAgc3FydCh3ZWlnaHRlZC5tZWFuKHRhcHBseShkZW5zaXR5LCBlZGUxLCB2YXIpLAogICAgICAgICAgICAgICAgICAgICAgICB0YXBwbHkoZGVuc2l0eSwgZWRlMSwgbGVuZ3RoKSAtIDEpKSkKCmVmZl9zaXplKHJlcGxpY2F0ZV9lbW0sIHNpZ21hID0gcG9vbGVkX3ZhciwKICAgICAgICAgZWRmID0gbGVuZ3RoKHNsYTFfZGVuc2l0eSRkZW5zaXR5KSAtIGxlbmd0aChsZXZlbHMoc2xhMV9kZW5zaXR5JGVkZTEpKSwKICAgICAgICAgbWV0aG9kID0gJ3RydC52cy5jdHJsJywgcmVmID0gMSkKCmVmZl9zaXplKHJlcGxpY2F0ZV9lbW0sIHNpZ21hID0gcG9vbGVkX3ZhciwKICAgICAgICAgZWRmID0gbGVuZ3RoKHNsYTFfZGVuc2l0eSRkZW5zaXR5KSAtIGxlbmd0aChsZXZlbHMoc2xhMV9kZW5zaXR5JGVkZTEpKSwKICAgICAgICAgbWV0aG9kID0gJ3RydC52cy5jdHJsJywgcmVmID0gMikKYGBgCgoKIyMjIEFkZGVuZHVtOiBtaXhlZCBtb2RlbCBhbmFseXNpcwoKVGhlIHAtdmFsdWVzIGZvciBkaWZmZXJlbnQgY29udHJhc3RzIGFib3ZlCmFyZSBhIHJlc3VsdCBvZiBhIHF1aXRlIGNvbnNlcnZhdGl2ZSBtb2RlbCwKd2hpY2ggaXRzZWxmIGlzIGEgcmVzdWx0IG9mIHN1Ym9wdGltYWwgZGVzaWduLiAKSW4gYSBwb29sZWQgb2JzZXJ2YXRpb24gbW9kZWwsIGZvciBleGFtcGxlLCB3ZSB3b3VsZCBmaW5kIAphIGNvdXBsZSBtb3JlIHNpZ25pZmljYW50IGVmZmVjdHMgZHVlIHRvIGxhcmdlciBzYW1wbGUgc2l6ZToKCmBgYHtyfQpwd3BtKGVtbWVhbnMocG9vbGVkX2xtLCAnZWRlMScpLCBkaWZmcyA9IEYpCmBgYAoKSGVyZSB3ZSBnZXQgbG93IHAtdmFsdWVzIGZvciB0aGUgZGlmZmVyZW5jZXMgYmV0d2VlbiBTbmY1IGFuZCBldmVyeXRoaW5nIGVsc2UsCmFzIHdlbGwgYXMg4oiGUFFDQyBhbmQgYWN0dWFsbHkgKmxlc3MqIGRlbnNlIEtoYy9lZGUx4oiGLgoKVGhpcyBtb2RlbCB3b3VsZCBnZW5lcmF0ZSBncm91cCBtZWFucyAKYW5kIGVmZmVjdCBzaXplcyBwcmV0dHkgbXVjaCB0aGUgc2FtZSBhcyB0aGUgbWVhbnMtcG9vbGVkIG1vZGVsLApidXQgZHJhc3RpY2FsbHkgZGlmZmVyZW50IHAtdmFsdWVzLgpUaGlzIHNlcnZlcyB0byB1bmRlcnNjb3JlIHRoYXQgcC12YWx1ZXMgCnNob3VsZCBiZSB0cmVhdGVkIHdpdGggaGVhbHRoeSBza2VwdGljaXNtLAphbmQgcGxheSBhIG1pbm9yIHJvbGUgaW4gdGhlIGludGVycHJldGF0aW9uIG9mIHRoZSByZXN1bHRzLgoKU29tZSB3b3VsZCBzYXkgdGhhdCB0aGlzIG1vZGVsIGlzIGFuIGV4YW1wbGUgb2YgKnBzZXVkb3JlcGxpY2F0aW9uKiwKYnV0IHRoaXMgaXMgYSBiaXQgb2YgYSBncmV5IGFyZWEuClllYXN0IGNlbGxzIGZyb20gZGlmZmVyZW50IGRhdGFzZXRzCmdyb3cgaW4gdGhlIHNhbWUgbWVkaXVtLCBpbmN1YmF0b3IgYW5kIHNvIG9uLApidXQgdGhleSBhcmUgaW5kaXZpZHVhbCBvcmdhbmlzbXMuCkV2ZW4gaWYgYSAnZGF0ZSBlZmZlY3QnIGV4aXN0cyBiZXlvbmQgYSBzaW1wbGUgc2FtcGxpbmcgdmFyaWF0aW9uLAp0aGVyZSBpcyBubyByZWFzb24gdG8gdGhpbmsgdGhhdCB0aGUgZWZmZWN0IGlzIGNvbnNpc3RlbnQKYWNyb3NzIG11dGFudHM6IGFmdGVyIGFsbCwgZWFjaCBtdXRhbnQgZ3Jvd3MgaW4gYSBzZXBhcmF0ZSB0dWJlLgpJZiB3ZSB0cnkgdG8gbG9vayBhdCBhbiBpbnRlcmFjdGlvbiBwbG90LAp3ZSBjYW5ub3Qgc2VlIGEgY29uc2lzdGVudCBkYXRlIGVmZmVjdDoKCmBgYHtyfQpnZ3Bsb3Qoc2xhMV9kZW5zaXR5LCBhZXMoeCA9IGFzX2ZhY3RvcihkYXRlKSwgeSA9IGRlbnNpdHksIGdyb3VwID0gZWRlMSwgY29sID0gZWRlMSkpICsgCiAgI3NjYWxlX2NvbG9yX2JyZXdlcihwYWxldHRlID0gIlNldDIiKSsKICBzdGF0X3N1bW1hcnkoZnVuID0gbWVhbiwgZ2VvbSA9ICJsaW5lIikrCiAgc3RhdF9zdW1tYXJ5KGZ1biA9IG1lYW4sIGdlb20gPSAicG9pbnQiKSsKICBndWlkZXMoeCA9IGd1aWRlX2F4aXMoYW5nbGUgPSAtNDUpKSsKICBsYWJzKHggPSAnRGF0ZScsIHkgPSAnU2xhMSBkZW5zaXR5JykKYGBgCgpPZiBjb3Vyc2UgdGhpcyBpcyB1bmRlcm1pbmVkCmJ5IHRoZSBkaXNjb25uZWN0IGluIGRhdGUgLyBtdXRhbnQgY29tYmluYXRpb25zLgoKQ2FuIHdlIGdlbmVyYXRlIGEgbGluZWFyIG1peGVkIG1vZGVsIGZvciB0aGlzLAp1c2luZyBgZGF0ZWAgYXMgYSByYW5kb20gZWZmZWN0PyAKQmVjYXVzZSB0aGVyZSBkb2VzIG5vdCBzZWVtIHRvIGJlIGFueSBjb25zaXN0ZW50IGVmZmVjdCBvZiBkYXRlIGluIGl0c2VsZiwKbGV0J3MgYWRkIGFuIGludGVyYWN0aW9uIHRlcm0uCgpgYGB7ciBlY2hvID0gVFJVRX0KZGF0ZV9sbW0xIDwtIGxtZXIoZGVuc2l0eSB+IGVkZTEgKyAoMXxkYXRlKSwgZGF0YSA9IHNsYTFfZGVuc2l0eSkKZGF0ZV9sbW0yIDwtIGxtZXIoZGVuc2l0eSB+IGVkZTEgKyAoMXxlZGUxOmRhdGUpLCBkYXRhID0gc2xhMV9kZW5zaXR5KQpkYXRlX2xtbTMgPC0gbG1lcihkZW5zaXR5IH4gZWRlMSArICgxfGRhdGUpICsgKDF8ZWRlMTpkYXRlKSwgZGF0YSA9IHNsYTFfZGVuc2l0eSkKYGBgCgpgYGB7cn0KYW5vdmEoZGF0ZV9sbW0xLCBkYXRlX2xtbTIsIGRhdGVfbG1tMykKYGBgCgpVc2luZyB0aGUgYGFub3ZhYCB0YWJsZSBmb3IgbWl4ZWQgbW9kZWxzIGZyb20gYGxtZXJUZXN0YAp3ZSBhY3R1YWxseSBmaW5kIHRoZSBpbnRlcmFjdGlvbi1vbmx5IG1vZGVsIGBkYXRlX2xtbTJgIGhhcyB0aGUgbG93ZXN0IEFJQy4KClNpbmdsZS10ZXJtIGRlbGV0aW9ucyBvZiBlZmZlY3RzIGZyb20gdGhlIG1haW4gKyBpbnRlcmFjdGlvbiBtb2RlbApgZGF0ZV9sbW0zYCBzdWdnZXN0IHRoYXQgdGhlIGludGVyYWN0aW9uIG1vZGVsIGlzIHNpZ25pZmljYW50CmFuZCB0aGUgbWFpbiBkYXRlIGVmZmVjdCBpcyBub3QuCgpgYGB7cn0KcmFub3ZhKGRhdGVfbG1tMykKYGBgCgpXaGF0IGRvZXMgdGhpcyBtZWFuPyBJIGFtIGdldHRpbmcgb3V0IG9mIG15IGRlcHRoIGhlcmUsCmJ1dCBJIHRoaW5rIHRoYXQgd2Ugc2ltcGx5IGNhbm5vdCBhc3NpZ24KYSBtZWFuaW5nZnVsIGNoYW5nZSBvZiBpbnRlcmNlcHQgYmFzZWQgb24gYGRhdGVgCmJlY2F1c2UgdGhlIGVmZmVjdCBpcyBkaWZmZXJlbnQgZm9yIGV2ZXJ5IEVkZTEgbGV2ZWwgKGhlbmNlIHRoZSBpbnRlcmFjdGlvbikuCkFnYWluLCB0aGlzIG1ha2VzIHNlbnNlIGJlY2F1c2Ugd2hpbGUgdGhlb3JldGljYWxseSB0aGVyZSBjb3VsZCBiZSBzb21lIGlzc3VlcwphZmZlY3RpbmcgYWxsIHN0cmFpbnMgb24gYSBnaXZlbiBkYXkgKHNheSwgaW5jdWJhdG9yIGZhaWx1cmUpLAp0aGlzIGp1c3QgZGlkIG5vdCBoYXBwZW4gYW5kIHNvIGl0J3Mgbm90IHJlZmxlY3RlZCBpbiB0aGUgZGF0YQpiZXlvbmQgYSBzaW1wbGUgc2FtcGxpbmcgZXJyb3IgYW5kIHRoYXQgaXMgZ29pbmcgdG8gYmUgdW5pcXVlIHRvIGVhY2ggc2FtcGxlLgoKTm90ZSB0aGF0IHRoaXMgaXMgdmVyeSB1bmxpa2UgdGhlIGRhdGFzZXQgaW4gRmlndXJlIDMsCndoZXJlIGFueSBjaGFuZ2UgaW4gdGhlIG1pY3Jvc2NvcGUgCndpbGwgYWZmZWN0IGludGVuc2l0eSByZWFkaW5ncy4KCkxldCdzIGxvb2sgYXQgd2hhdCB0aGUgaW50ZXJhY3Rpb24tb25seSBtb2RlbApzYXlzIGFib3V0IGBlZGUxYCBlZmZlY3RzIG9uIGBkZW5zaXR5YC4KRmlyc3QsIGhvdyBkbyBtb2RlbCByZXNpZHVhbHMgbG9vaz8KUHJldHR5IGdvb2QsIGJ1dCBpdCdzIG5vdCBsaWtlIHRoZSBwb29sZWQKb2JzZXJ2YXRpb24tb25seSBtb2RlbCBoYWQgYW55IHByb2JsZW1zLgoKYGBge3J9CnBsb3QoZGF0ZV9sbW0yKQoKcGFyKG1mcm93ID0gYygxLCAyKSkKCnFxbm9ybShyYW5lZihkYXRlX2xtbTIpJCJlZGUxOmRhdGUiWywgMV0sIAogICAgICAgbWFpbiA9ICJSYW5kb20gZWZmZWN0cyBvZiBpbnRlcmFjdGlvbiIpCnFxbm9ybShyZXNpZChkYXRlX2xtbTIpLCBtYWluID0gIlJlc2lkdWFscyIpCmBgYAoKRXZlbiBpZiB3ZSBhY2NlcHQgdGhlIGludGVyYWN0aW9uLW9ubHkgbW9kZWwsCnRoZSByYW5kb20gZmFjdG9yIGRvZXMgbm90IGV4cGxhaW4gbXVjaCBvZiB0aGUgdmFyaWFuY2UgYW55d2F5OgoKYGBge3J9CnN1bW1hcnkoZGF0ZV9sbW0yKQpgYGAKCldlIGhhdmUgYHIgcm91bmQoMTAwICogMC4wMDA2MTg0IC8gKDAuMDAwNjE4NCArIDAuMDEwODgxMykpYCUgb2YgdmFyaWFuY2UgCmV4cGxhaW5lZCBieSB0aGUgYDF8ZWRlMTpkYXRlYCB0ZXJtIGluIHRoZSBtb2RlbC4KCkxldCdzIHF1aWNrbHkgZ2VuZXJhdGUgZ3JvdXAgbWVhbnMgKGRpYWdvbmFsKQphbmQgcC12YWx1ZXMgZm9yIGNvbnRyYXN0cywgd2l0aCBUdWtleSBhZGp1c3RtZW50OgoKYGBge3J9CnB3cG0oZW1tZWFucyhkYXRlX2xtbTIsICdlZGUxJyksIGRpZmZzID0gRikKYGBgCgpUaGUgdmFsdWVzIGFyZSBzdHJpa2luZ2x5IHNpbWlsYXIKdG8gd2hhdCB3ZSBzYXcgd2l0aCB0aGUgbWVhbnMtcG9vbGVkIG1vZGVsLgpXaHk/IEkgc3VzcGVjdCB0aGF0IHRoZXNlIGFyZSBhY3R1YWxseSBlcXVpdmFsZW50CmJlY2F1c2UgdGhlIG1peGVkIG1vZGVsIHdlIGVuZGVkIHVwIHdpdGgKY3JlYXRlcyAyNyB1bmlxdWUgZ3JvdXBzIHdpdGggbm8gc2hhcmVkIGluZm9ybWF0aW9uCmJ5IGBlZGUxYCAvIGBkYXRlYCBjb21iaW5hdGlvbgpqdXN0IGFzIHdlIGhhdmUgMjcgYGVkZTFgIC8gYGRhdGFzZXRgIHVuaXF1ZSBncm91cCBtZWFucwppbiB0aGUgYHJlcGxpY2F0ZV9sbWAgbW9kZWwuCgojIyBDb25jbHVzaW9ucwoKIyMjIEFjdHVhbCBjb25jbHVzaW9ucwoKMS4gQWxsIG11dGF0aW9ucyBjYXVzZSBhIHNpZ25pZmljYW50IHJlZHVjdGlvbiBpbiBwYXRjaCBkZW5zaXR5IGZyb20gd2lsZCB0eXBlCjIuIFdoaWxlIEVkZTHiiIZQUUNDIGlzIG5vdCAqc2lnbmlmaWNhbnRseSogZGlmZmVyZW50IGZyb20gZWRlMeKIhiwgCiAgaXQgaGFzIHRvIGJlIG5vdGVkIHRoYXQgdGhlIGVzdGltYXRlIGlzIGhpZ2hlcgogICh+NDAlIHJlZHVjdGlvbiBpbnN0ZWFkIG9mIH41MCUpLgozLiBTbmY1IGFuZCBTdXAzNSBzdWJzdGl0dXRpb25zIGhhdmUgc2lnbmlmaWNhbnRseSBoaWdoZXIgU2xhMSBkZW5zaXRpZXMgdGhhbiBlZGUx4oiGOwogIFN1cDM1IGlzIGFsc28gc2lnbmlmaWNhbnRseSBkaWZmZXJlbnQgZnJvbSDiiIZQUUNDLgo0LiBBbGwgdGhlIG90aGVyIG11dGFudHMgYXJlIHNpbWlsYXIgYWNyb3NzIHRoZSBib2FyZC4KNS4gU3RlcHBpbmcgYXdheSBmcm9tIHAtdmFsdWVzOiBJRFJzIHJlc2N1ZSBTbGExIGRlbnNpdHkgb25seSBzb21ld2hhdCwgCiAgdGhlIG90aGVycyBub3QgYXQgYWxsLiBUaGF0IGlzIHRoZSB0YWtlLWhvbWUgbWVzc2FnZS4KCiMjIyBGaW5hbCBlc3RpbWF0ZXMKCkZpbmFsIGVzdGltYXRlcyB3aXRoIGxvd2VyIC8gdXBwZXIgOTUlIGNvbmZpZGVuY2UgaW50ZXJ2YWxzLAphbmQgYSBjb21tb24tc2Vuc2UgY29tcGFyaXNvbiB0byB3aWxkIHR5cGUgKGluICUpLiAKYGhhbGZfY2lgIGlzIGhhbGYgb2YgdGhlIGNvbmZpZGVuY2UgaW50ZXJ2YWwsCmZvciB3cml0aW5nIENJIHJhbmdlcyBpbiB0aGUgZm9ybWF0IG1lYW4gKy8tIGVycm9yLgoKYGBge3J9Cnd0X21lYW4gPC0gc2xhMV9kZW5zaXR5X3N0YXRzICU+JQogIGZpbHRlcihlZGUxID09ICdFREUxJykgJT4lCiAgcHVsbChkZW5zaXR5X21lYW4pICU+JQogIG1lYW4oKQoKZGVuc2l0eV9jaSA8LSBzbGExX2RlbnNpdHlfc3RhdHMgJT4lCiAgZ3JvdXBfYnkoZWRlMSklPiUKICBzdW1tYXJpc2UobWVhbl9jbF9ub3JtYWwoZGVuc2l0eV9tZWFuKSkgJT4lCiAgcmVuYW1lKG1lYW4gPSB5LCBsb3dlciA9IHltaW4sIHVwcGVyID0geW1heCkgJT4lCiAgbXV0YXRlKHByb2Nfd3QgPSByb3VuZCgxMDAgKiBtZWFuIC8gd3RfbWVhbiksCiAgICAgICAgIGhhbGZfY2kgPSAodXBwZXIgLSBsb3dlcikgLyAyKSAKCmthYmxlKGRlbnNpdHlfY2ksIGRpZ2l0cyA9IDMpCmBgYAoKIyMgU291cmNlIGRhdGEKCiMjIyAuY3N2CgpgYGB7ciBlY2hvPUZBTFNFfQp4ZnVuOjplbWJlZF9maWxlKCdkYXRhL2lkcl9yZXBsYWNlbWVudF9zbGExLmNzdicpCmBgYAoKIyMjIC5SRGF0YQoKYGBge3IgZWNobz1GQUxTRX0KeGZ1bjo6ZW1iZWRfZmlsZSgnZGF0YS9pZHJfcmVwbGFjZW1lbnRfc2xhMS5SRGF0YScpCmBgYAoKIyMgU2Vzc2lvbiBpbmZvCgpgYGB7ciBzZXNzaW9uLCBtZXNzYWdlPVRSVUV9CnNlc3Npb25JbmZvKCkKYGBgCgojIyBXSVAKCmBgYHtyfQp3aXRoKHNsYTFfZGVuc2l0eSwgaW50ZXJhY3Rpb24ucGxvdCh4LmZhY3RvciA9IGFzLmZhY3RvcihkYXRlKSwgdHJhY2UuZmFjdG9yID0gZWRlMSwgCiAgICAgICAgICAgICAgICAgICAgICAgICAgICAgICByZXNwb25zZSA9IGRlbnNpdHksIGxhcyA9IDIsIHR5cGUgPSAnYicpKQpgYGAK
